# Imputation method for single-cell RNA-seq data using neural topic model

**DOI:** 10.1093/gigascience/giad098

**Published:** 2023-11-24

**Authors:** Yueyang Qi, Shuangkai Han, Lin Tang, Lin Liu

**Affiliations:** Yunnan Normal University, School of Information, Kunming 650500, China; Yunnan Normal University, School of Information, Kunming 650500, China; Yunnan Normal University, Faculty of Education, Kunming 650500, China; Yunnan Normal University, School of Information, Kunming 650500, China

**Keywords:** Single-cell RNA sequencing, imputation, dropout, neural topic model

## Abstract

Single-cell RNA sequencing (scRNA-seq) technology studies transcriptome and cell-to-cell differences from higher single-cell resolution and different perspectives. Despite the advantage of high capture efficiency, downstream functional analysis of scRNA-seq data is made difficult by the excess of zero values (i.e., the dropout phenomenon). To effectively address this problem, we introduced scNTImpute, an imputation framework based on a neural topic model. A neural network encoder is used to extract underlying topic features of single-cell transcriptome data to infer high-quality cell similarity. At the same time, we determine which transcriptome data are affected by the dropout phenomenon according to the learning of the mixture model by the neural network. On the basis of stable cell similarity, the same gene information in other similar cells is borrowed to impute only the missing expression values. By evaluating the performance of real data, scNTImpute can accurately and efficiently identify the dropout values and imputes them accurately. In the meantime, the clustering of cell subsets is improved and the original biological information in cell clustering is solved, which is covered by technical noise. The source code for the scNTImpute module is available as open source at https://github.com/qiyueyang-7/scNTImpute.git.

## Introduction

Bulk-cell RNA sequencing (RNA-seq) techniques have been widely used for transcriptome analysis to study transcriptional structure, splicing patterns, and expression levels of genes and transcriptomes [1]. To address biological issues such as cell heterogeneity and gene expression randomness, it is particularly important to interpret cell-specific transcriptome landscapes [2]. Although the bulk-cell RNA-seq technique is popular, it measures the average expression level of genes in batch cells, and the expression of variable genes will be pulled to average. Therefore, it is not possible to study cell specificity based on transcriptomics. Fortunately, by studying gene expression status in single cells, scRNA-seq technology overcomes the shortcomings of traditional batch cell sequencing technology and is becoming a powerful tool to capture the intercell variability of the transcriptome. It has dramatically changed the study of transcriptomics, helping us to decode life from a higher-resolution and spatiotemporal structure, accurately reflecting the heterogeneity between cells. The study of scRNA-seq data has become a hot subject today.

Currently, we use multiple single-cell RNA-seq (scRNA-seq) platforms, the two most popular being Fluidigm and Drop-Seq. The Drop-Seq processes thousands of cells in a single run, which not just saves time and cost but also is simple to operate. Fluidigm, while it usually processes fewer cells, has higher coverage rates. So, an increasing number of studies are using these techniques to discover new cell types [3, 4], new markers for specific cell types [3, 5, 6], and cell heterogeneity [6–11].

However, scRNA-seq technology has its corresponding drawbacks. scRNA-seq data have a relatively higher noise level than batch cell RNA-seq data, resulting in a major problem that is the sparsity of the gene expression matrix (i.e., the data often exhibit a large number of zero values) [12]. Most of these zeros are artificially caused by defects in sequencing techniques, including, but not limited to, inadequate gene expression, low capture rates and sequencing depth, or other technical factors [13, 14]. As a result, the observed zero value does not reflect the underlying true expression level [15, 16]. This gene expression bias may be further increased during subsequent amplification steps. Thus, dropout events can significantly affect downstream bioinformatics analysis. At present, researchers have proposed a variety of imputation models through different principles and methods [17, 18]. These research results have a great guiding role in scRNA-seq data integration, enrichment analysis, and so on. According to the design characteristics of the imputation algorithm, as well as the data feature learning and processing methods, we roughly divide the RNA-seq data imputation methods into two categories: deep learning–based imputation method and non–deep learning imputation method [19].

In the traditional non–deep learning imputation algorithm, because of its simple idea, it is able to usually fit the corresponding statistical probability model or use the expression matrix for smoothing and diffusion. So, there are certain advantages in some specific types of samples. Wagner et al. [20] used the k-nearest neighbors (KNN) smoothing method by finding *k*-nearest neighbors between cells and aggregating gene-specific UMI (Unique Molecular Identifiers) counts to impute the gene expression matrix. In finding the number of nearest neighbors *k*, instead of using a way to fit a certain model, the data’s imputation is achieved stepwise by constructing a partially smoothed profile with a variance-stabilizing transformation. Li and Li [21] introduced a statistical method, scImpute, which uses a mixture model to learn the loss probability of each gene in each cell. By setting a loss probability threshold, the input data are divided into two parts: the set of genes severely affected by “dropout” *A_j_* and the set of unaffected genes *B_j_*. Eventually, the information on similar cells is learned from *B_j_* for imputation. scImpute automatically identifies possible dropout values and performs imputation only on these values without introducing new biases to the rest of the data. Huang et al. [22] proposed the SVAER algorithm, which is a method that uses information across genes and cells to impute zero values so as to optimize the expression of all genes. By looking for potential relationships between genes, the true expression level of each gene in each cell can be restored, eliminating technical differences. Nevertheless, SVAER alters all gene expression levels, including those not affected by dropout events, which could introduce new biases into the data and potentially eliminate biologically significant variation. For scRNA-seq data that are large, often high-dimensional, sparse, and complex, analysis using traditional computational methods becomes difficult and infeasible [23, 24].

As deep neural network algorithms have gained great application in biomedical fields in recent years, they mine complex relationships within single-cell data through a series of basic hierarchical operations. The typical deep learning algorithms applied to scRNA-seq data are autoencoders (AEs), variational autoencoders (VAEs), generative adversarial networks (GANs), and other models. Eraslan et al. [25] proposed the deep count autoencoder (DCA) network model by improving the conventional autoencoder. The reconstruction error is defined as the probability of the noise model distribution rather than the reconstruction of the input data themselves. Gene-specific distribution parameters are learned by minimizing reconstruction errors in an unsupervised manner. The noise model is eventually applied to sparse count data, giving it a loss function specifically for scRNA-seq data. Meanwhile, its deep learning framework is capable of capturing the complexity and nonlinearity of scRNA-seq data and is highly scalable. Arisdakessian et al. [26] proposed a deep neural network–based imputation algorithm (DeepImpute) by constructing multiple subneural networks, which impute genes in a divide-and-conquer manner, not only achieving the highest overall accuracy but also providing faster computing time and requiring less memory. Xu et al. [27] proposed a scRNA-seq data imputation method (scIGANs) founded on generative adversarial network. The method uses networks to generate cells rather than cells observed in the original matrix to balance the performance between dominant and rare cell populations, enabling it to learn nonlinear gene-to-gene dependencies from complex samples of multicellular types and train generative models to generate realistic expression profiles of defined cell types. After training, KNN is used to impute the same type of cells, thereby eliminating technical variations without damaging intercell biological variability. This method is robust to small data with low expression or intercell differences [27–29].

Most downstream analyses of scRNA-seq, such as differential gene expression analysis, cell-type specific gene identification, and new cell-type definition, rely on the accuracy of gene expression measurements. Therefore, it is particularly important to correct the expression of “false zero values” caused by dropout events in scRNA-seq data through accurate and robust imputation methods [21]. These imputation methods identify the dropout values in scRNA-seq data from different perspectives and impute them. However, for non–deep learning, it is impossible to effectively learn the feature relationship of some complex nonlinear data, and it does not have good flexibility and expansibility. The architecture of deep learning itself is a “black box,” with many learning layers and thousands of nodes, making the underlying features learned and the full rich potential of the single-cell dataset unleashed uninterpretable [30].

Although the study of RNA-seq data is an active area of research, accurate recovery of single-cell gene expression data remains a great challenge. Inspired by the neural topic, we have designed an accurate and stable imputation method, called scNTImpute, that can more precisely impute gene expression affected by dropout. Specifically, scNTImpute performs deep feature extraction and the construction of networks of encoders through the coding learning mechanism of transferable neural networks, learning network parameters and highly interpretable mixtures of cell topic from scRNA-seq data. Topic features can be used to learn the similarity of cells, and researchers are capable of performing topic pathway enrichment analysis on them at a later stage. This is used to explore whether they have relevance to currently known gene pathways, as well to uncover topics that may be condition specific or cell type specific to improve the interpretability of the deep feature from a biological perspective. Concurrently, we will get underlying connections such as cell to cell, cell to gene, or gene to gene in single-cell data. The flexibility of the neural topic model makes it excellent for processing scRNA-seq data. Besides, scNTImpute uses neural networks to learn the mixture model parameters of gene expression distribution, solving the dropout probability of each gene in each cell. This allows us to more directly understand the true state of the expression data of the scRNA-seq transcriptome and distinguish which gene transcripts are affected by dropout. Using information about the same gene in other similar cells allows us to impute the dropout value in a cell through underlying cell–gene connections. Prior to this, it has been important that the borrowed information is selected for genes that are as free as possible from dropout events.

## Results

### scNTImpute model overview

**Figure 1:**
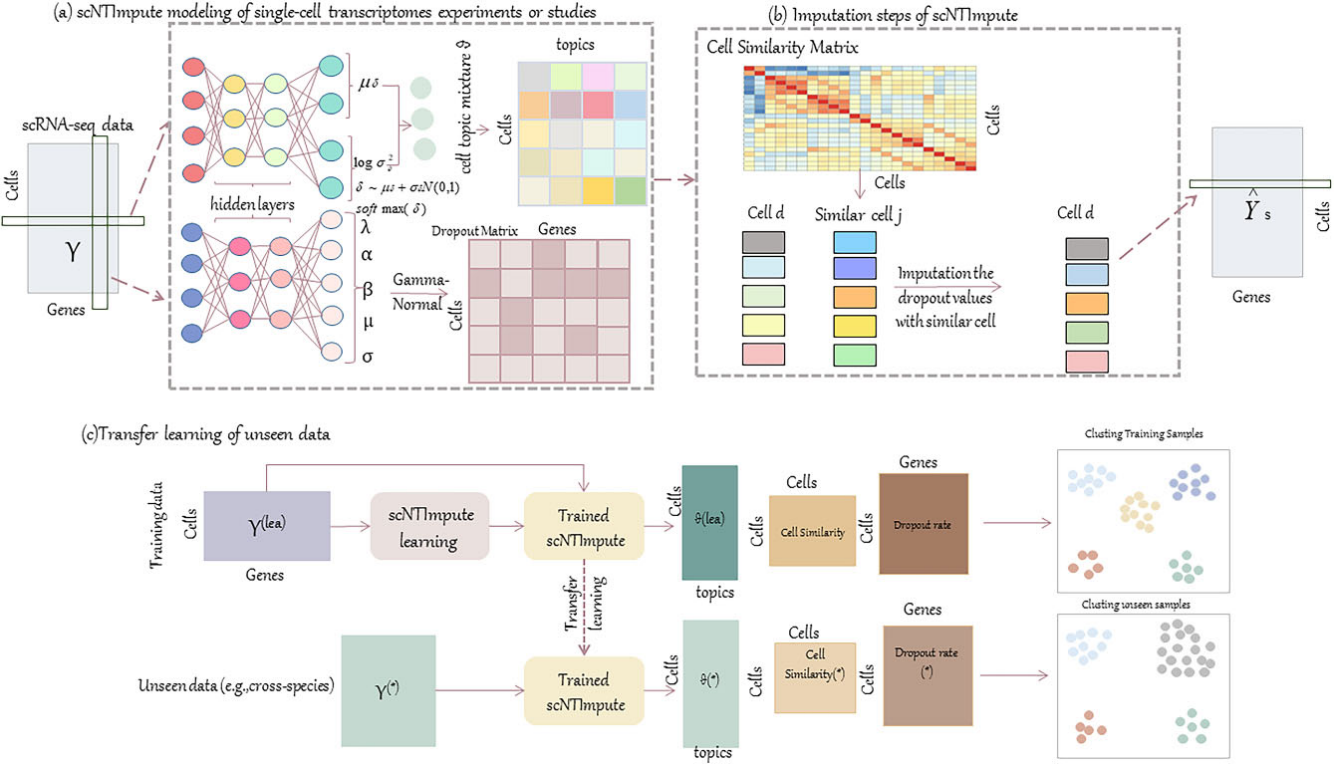
Workflow of scNTImpute. (A) scNTImpute uses a neural topic network architecture to model the single-cell transcriptome. Normalized counts of the gene expression data matrix and its transpose matrix for each single-cell dataset are used as input to the encoder. The encoder network generates random samples of potential cell-topic mixtures (*θ_d_*, cells *d* = 1, …, *N*) that can be used to compute intercell similarity. Neural networks learn the parameters of a mixture model of gene expression data and can be used to identify dropout values. (B) Imputation works using similar cell information. A cell similarity matrix is generated by calculating the intercellular similarity from the resulting mixture of the cell topic. In view of the learned parameters of the mixture model, the dropout value is identified and imputed with the information of similar cells (cell *j*) of the cell where the dropout value is located (cell *d*). (C) Transfer learning workflow. The scNTImpute model trained on the reference scRNA-seq dataset can infer the mixture of cell topic *θ* and the mixture model distributions from the unseen scRNA-seq dataset and perform accurate imputation on the unseen dataset. The scRNA-seq dataset is visualized by UMAP and evaluates by standard unsupervised clustering metrics using real cell types.

We propose a new scRNA-seq data imputation method on account of a neural topic model that is adapted from the single-cell embedded topic model (scETM), which inherits the advantages of topic modeling and is very effective in dealing with heavy-tailed and large distributions of word frequencies [31, 32]. For the analysis of the scRNA-seq data study, we pass the sampled cell and transcriptome expressions separately as vectors of normalized counts to two fully connected neural networks (i.e., tow-layer fully connected encoders). First, using a fully connected neural network encoder, we infer the topic mixing ratio of cells, namely, the cell-topic mixture (Fig. 1A). Second, we use the second neural network to infer the mixed distribution parameters of the transcriptome and obtain probability estimates of whether the gene expression value in each cell is a dropout value by using the mixed distribution model. Finally, the cell-topic mixture infers similar cells of the cell in which the dropout gene is located and is the same genetic information from similar cells for the imputation of dropout values (Fig. 1B).

### scNTImpute can efficiently impute scRNA-seq data

**Figure 2:**
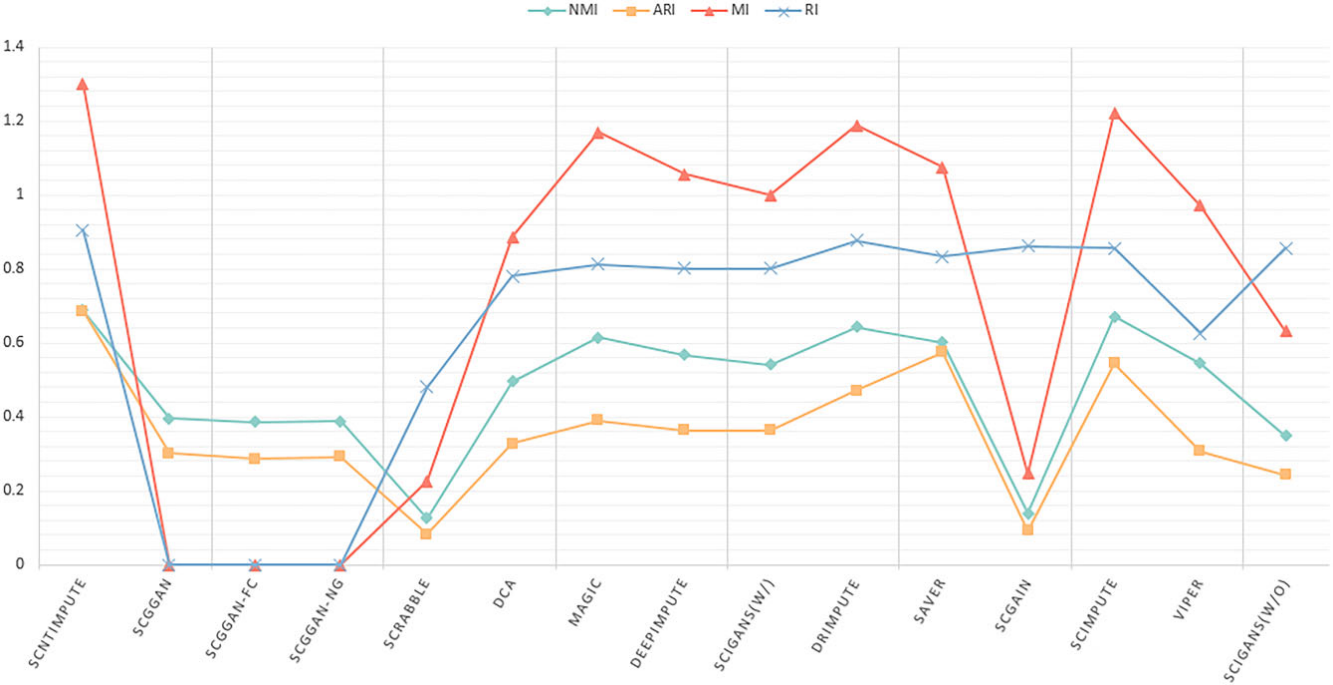
Visualization of human brain scRNA-seq data imputation metrics results.

**Figure 3:**
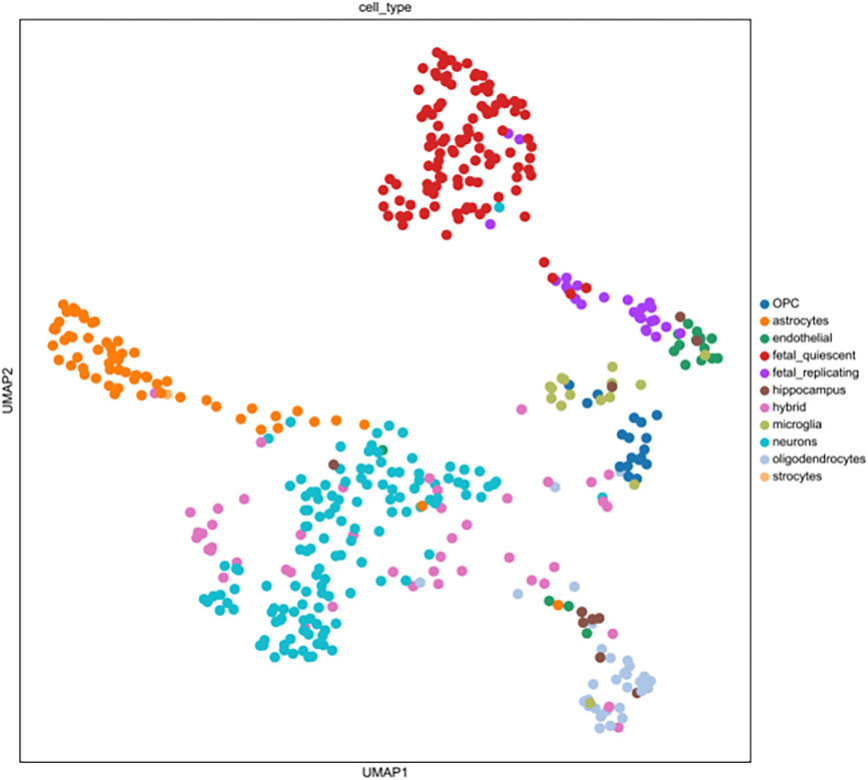
Clustering of human brain scRNA-seq data after imputation by scNTImpute.

Recovery of biologically significant gene expression from dropout events is the primary goal of scRNA-seq imputation, which can further reduce the impact on downstream analysis. In order to accurately evaluate the imputation performance of different models, we use published real datasets for experiments (including human brain single-cell datasets [33], Chung et al. [34]). scNTImpute stably provides competitive results. To intuitively see the imputation performance between models, 4 indexes are adopted as the benchmark: adjusted Rand index (ARI), Rand index (RI), normalized mutual information (NMI), and mutual information (MI). To be specific, we used scNTImpute and several other advanced imputation methods to evaluate real human brain scRNA-seq datasets: scGGAN (scGGAN-fc, scGGAN-ng) [35], SCRABBLE [36], DCA [25], MAGIC [37], DeepImpute [26], scIGANs(w/) [27], AutoImpute [38], DrImpute [39], ENHANCE [40], SAVER [22], scGAIN [41], scImpute [21], VIPER [42], and scIGANs(w/o) [27]. By visualizing the evaluation results (Fig. 2), we can intuitively see that the values of the 4 imputation evaluation indicators of scNTImpute are relatively high (specific imputation comparison results are shown in Table 1). After imputation, we used Leiden [43] clustering and UMAP visualization for the complete scRNA-seq data (Fig. 3). The results show that scNTImpute accurately and effectively recovers biologically significant gene expression from single-cell datasets.

**Table 1:** Multiple metrics used to measure the imputing output of multiple imputation methods on real scRNA-seq data from the human brain

	NMI	ARI	MI	RI
scNTImpute	0.6873	0.6857	1.3015	0.906
scGGAN	0.395	0.301	NA	NA
scGGAN-fc	0.386	0.286	NA	NA
scGGAN-ng	0.389	0.292	NA	NA
SCRABBLE	0.126	0.083	0.225	0.48
DCA	0.496	0.328	0.886	0.78
MAGIC	0.615	0.39	1.169	0.812
DeepImpute	0.568	0.364	1.057	0.802
scIGANs(w/)	0.54	0.364	0.999	0.802
DrImpute	0.642	0.471	1.189	0.876
SAVER	0.602	0.575	1.076	0.833
scGAIN	0.138	0.092	0.246	0.862
scImpute	0.672	0.545	1.223	0.856
VIPER	0.544	0.306	0.972	0.626
scIGANs(w/o)	0.349	0.243	0.631	0.631

**Figure 4:**
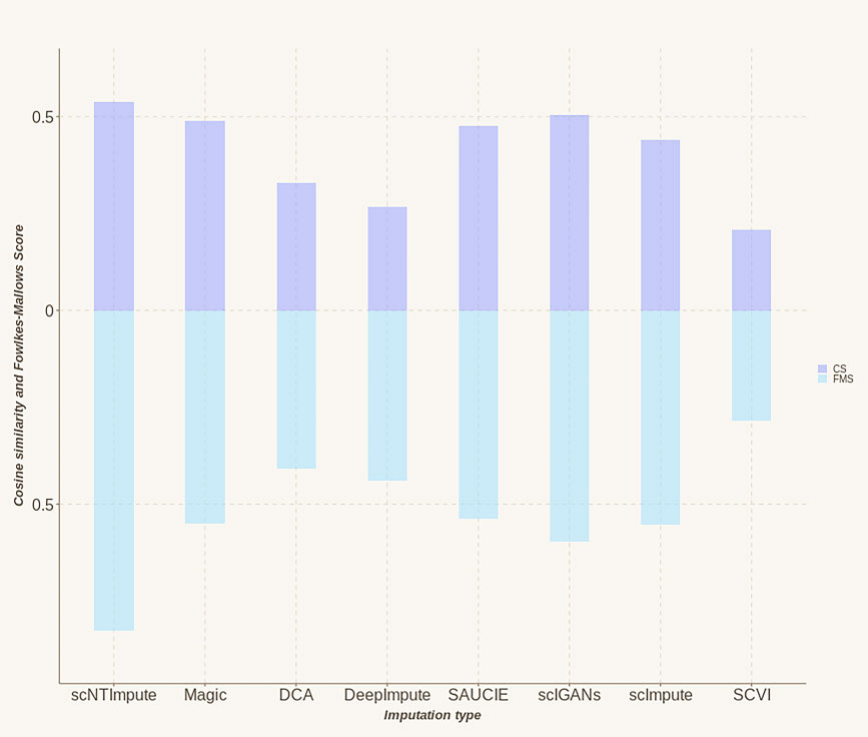
Comparison of imputation results of different methods for Chung et al. [34] data.

**Figure 5:**
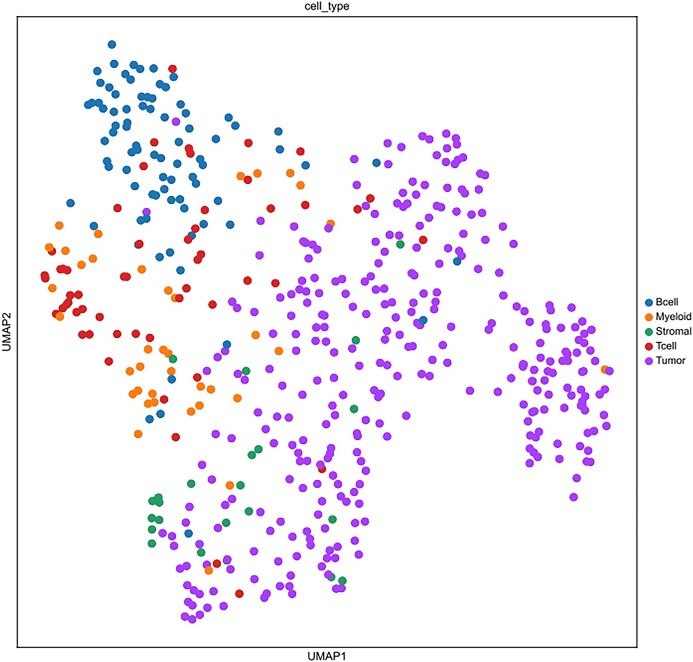
Clustering after imputation of Chung et al. [34] data using scNTImpute.

We performed imputation experiments on another published real dataset, Chung et al. [34]. The above imputation indexes are not the only criteria for evaluating the imputation of RNA-seq data. Different from the above, the other two imputation indexes have been used for evaluation (cosine similarity [CS], Fowlkes–Mallows score [FMS]). Similarly, we compared it with several other existing excellent imputation models. The evaluation results were visualized (Fig. 4), from which we could see that our model performed the best in both CS and FMS. Especially in FMS, a large gap was drawn with other imputation methods. (The specific imputation comparison results are shown in Table 2, and Fig. 5 shows the clustering effect on the complete Chung et al. [34] data set).

**Table 2:** Imputing output of multiple imputation methods on real Chung et al. [34] scRNA-seq data using different metrics

	CS	FMS
scNTImpute	0.5394	0.8264
Magic	0.4890	0.5493
DCA	0.3280	0.4080
DeepImpute	0.2668	0.4392
SAUCIE	0.4762	0.5372
scIGANs	0.5048	0.5961
scImpute	0.4413	0.5531
SCVI	0.2071	0.2833

**Figure 6:**
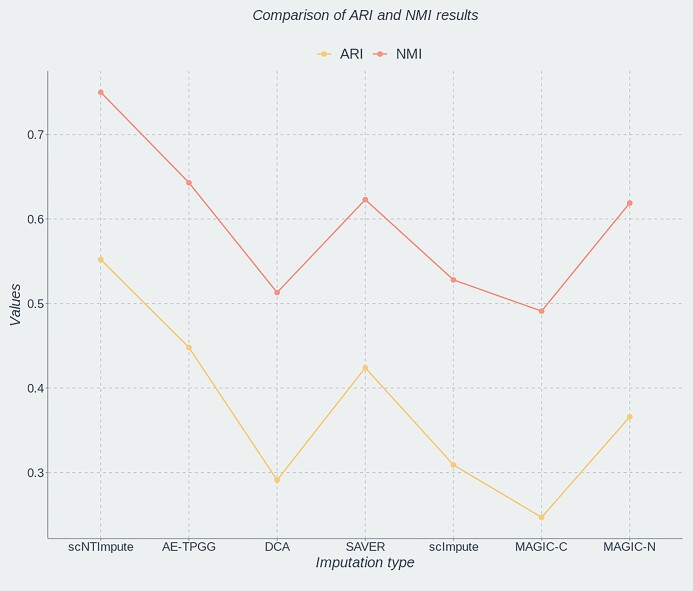
Comparison of results after imputation of Deng et al. [48] data by various methods.

**Figure 7:**
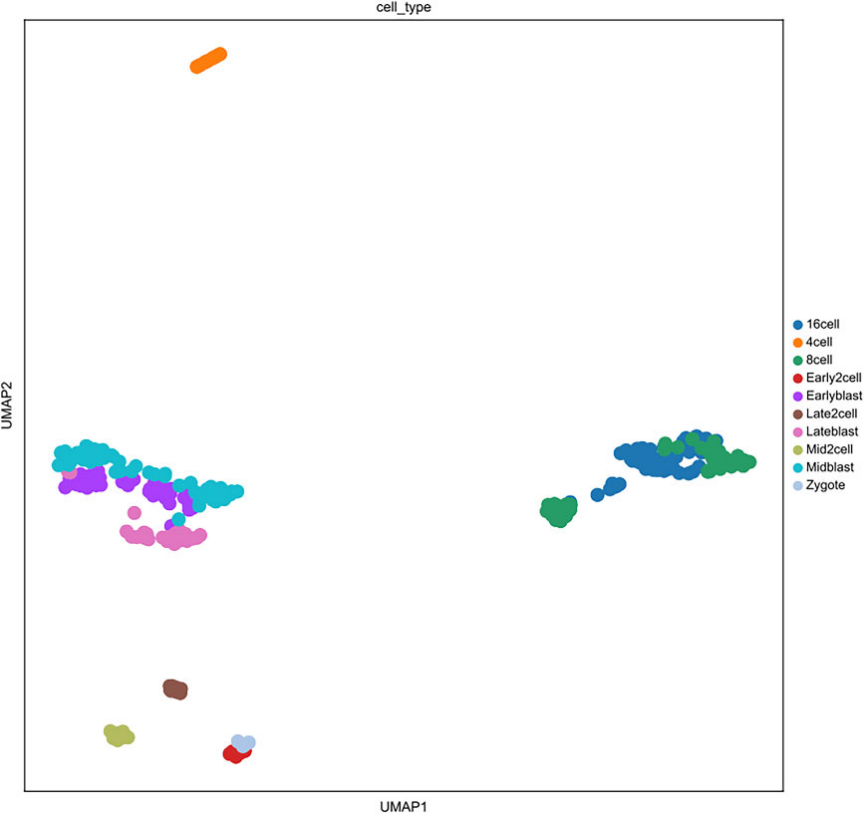
The clustering of the Deng et al. [48] dataset after imputation using scNTImpute.

To more effectively validate the robustness and stability of scNTImpute, as well as highlight the strengths of our model, we applied scNTImpute to more diverse real scRNA-seq datasets. In addition to the existing comparison methods mentioned above, we included several more advanced imputation methods for comparison (i.e., AE-TPGG [44], scGNN [45], scISR [46], scScope [47]). Besides, since the cell–cell distance matrix in MAGIC is based on Euclidean distances, the added MAGIC-C method is based on counting the data to understand how the form of the data affects the imputation. For the convenience of comparison and differentiation, the original MAGIC method based on normalized data is referred to as MAGIC-N [44]. First, we applied the model to a temporal scRNA-seq dataset involving mouse preimplantation embryonic development data (Deng et al. [48]). The Deng et al. [48] dataset includes single cells from 10 early mouse developmental stages, including zygotes; 2-, 4-, 8-, and 16-cell stages; and blastocysts [39]. We compared scNTImpute with a new imputation method called AE-TPGG on this dataset, using the aforementioned evaluation metrics (ARI, NMI). We visualized the imputation results (Fig. 6), from which we could observe that scNTImpute achieved the highest scores in both metrics, followed by our newly added AE-TPGG imputation method (Fig. 7 shows the clustering of the Deng dataset after imputation using scNTImpute). Specific imputation result data can be found in Table 3.

**Table 3:** Measurement of the imputation results of multiple methods on real Deng et al. [48] scRNA-seq data.

	ARI	NMI
scNTImpute	0.552	0.750
AE-TPGG	0.448	0.643
DCA	0.291	0.513
SAVER	0.424	0.623
scImpute	0.309	0.528
MAGIC-C	0.247	0.491
MAGIC-N	0.366	0.619

**Figure 8:**
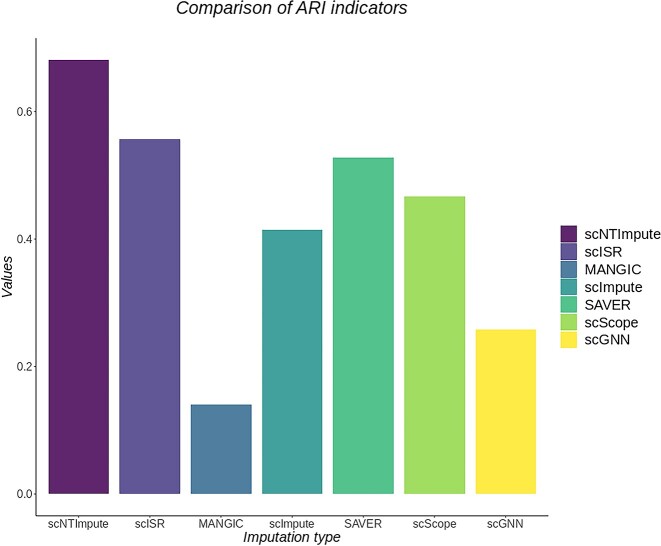
Results of different imputation methods on the human islet dataset [49].

**Figure 9:**
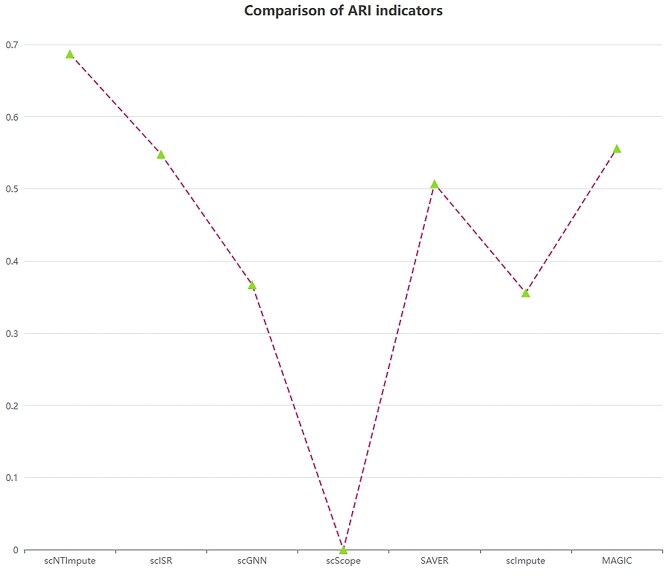
Results of different imputation methods in the Romanov et al. [50] dataset.

On two additional real datasets (human pancreatic islet [HP] [49], Romanov et al. [50]), we compared three new imputation methods. scScope is a scalable deep learning–based approach. The scGNN employs a graph neural network, which provides a hypothesis-free deep learning framework for scRNA-seq analysis. In contrast, scISR is a single-cell imputation method that utilizes subspace regression. We applied these three new methods alongside our model to these two real datasets. The evaluation was performed using the same metric, ARI. To visualize the results more intuitively, we plotted the imputation results of these methods (Figs. 8 and 9 represent the imputation results for the HP dataset [49] and Romanov et al. [50] dataset, respectively). From the figures, it is evident that scNTImpute achieved ARI scores close to 0.7 on both datasets, outperforming the other imputation methods (achieving the highest ARI). This further confirms scNTImpute’s effectiveness in recovering true biological information from sparse single-cell data.

### scNTImpute improves the clustering of cell subpopulations

**Figure 10:**
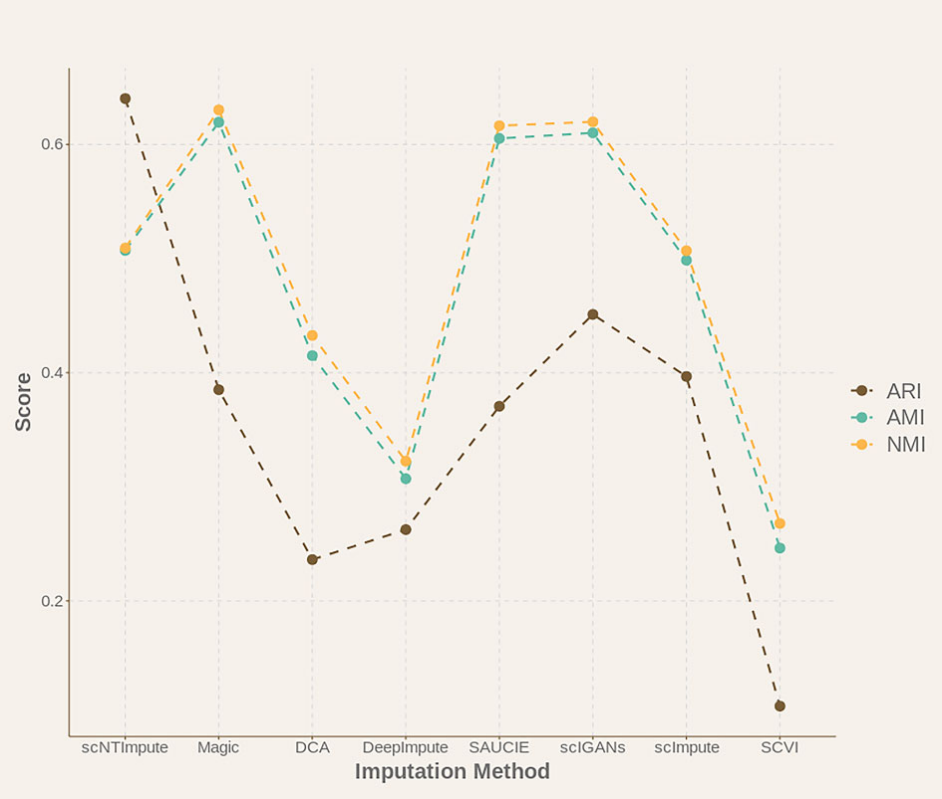
Effectiveness of different imputation methods in clustering Chung et al. [34] data.

**Figure 11:**
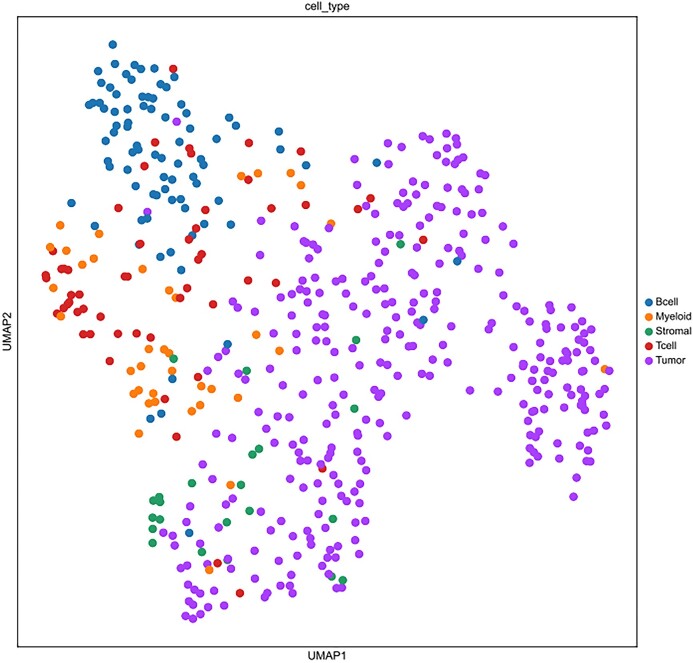
The clustering effect of using scNTImpute to impute Chung et al. [34] data.

To test the ability of scNTImpute to improve cell type or cell subgroup clustering, we applied scNTImpute to real scRNA-seq (i.e., also on the Chung et al. [34] dataset). In addition to reusing the above ARI and NMI evaluation indexes, we also adopted another commonly used clustering index, adjusted mutual information (AMI). We imputed scRNA-seq data with scNTImpute and other different imputation models, as well as compared cell clustering with complete imputation data. Through the comparison of evaluation data (refer to Table 4 for specific data and Fig. 10 for visualization of comparative data), our imputation method was the highest in the ARI index and had relatively significant and stable performance in the AMI and NMI clustering indexes (Fig. 11 shows the clustering effect after imputation).

**Table 4:** Clustering performance comparison of multiple methods in Chung et al. [34] data

	ARI	AMI	NMI
scNTImpute	0.6403	0.5071	0.5093
Magic	0.3851	0.6195	0.6304
DCA	0.2362	0.4150	0.4327
DeepImpute	0.2625	0.3071	0.3225
SAUCIE	0.3706	0.6053	0.6165
scIGANs	0.4511	0.6102	0.6199
scImpute	0.3967	0.4986	0.5069
SCVI	0.1078	0.2463	0.2679

**Figure 12:**
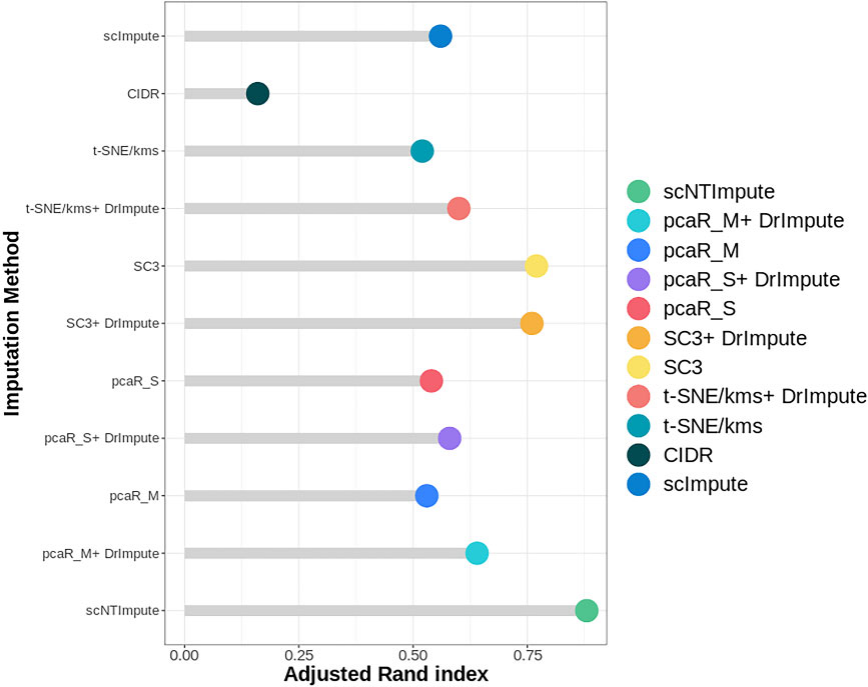
Clustering performance of multiple imputation methods on Hrvatin real data [3].

**Figure 13:**
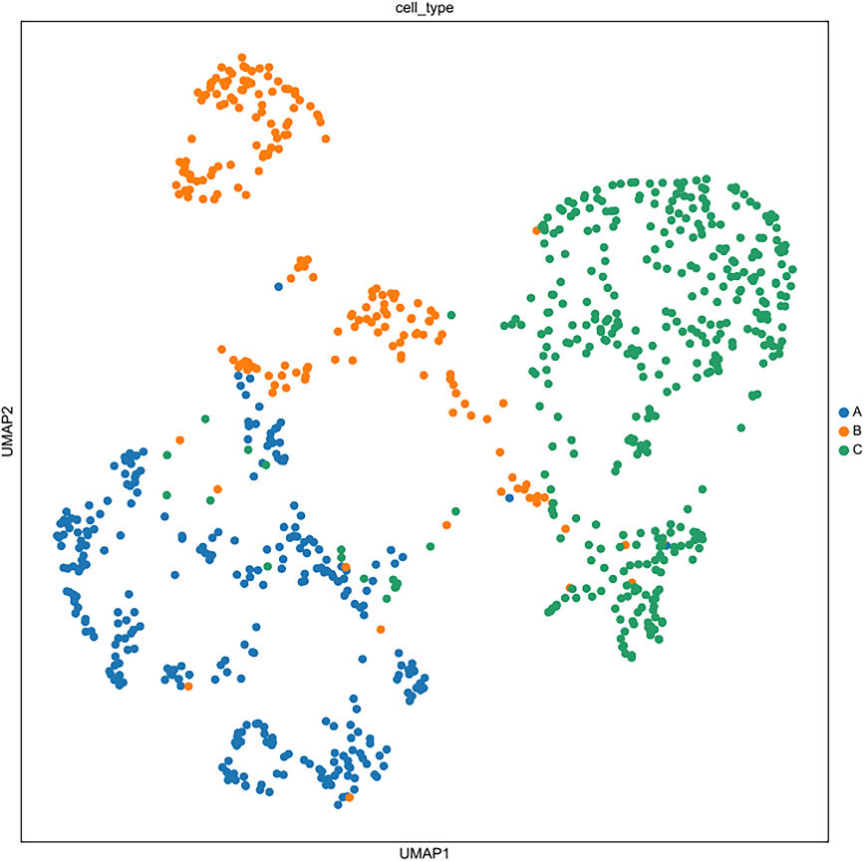
The clustering effect after imputing Hrvatin using scNTImpute.

Moreover, we evaluated the clustering effect of scRNA-seq data after imputation on another Hrvatin real data set [3]. We used the cell type stated in the original publication as the basic fact and ARI as a performance indicator. Unlike the previous comparison, here we combined the scNTImpute with other developed imputation models and clustering methods to evaluate; that is, before using the clustering algorithm, we used other imputation models to process and compare the results with our model. Several excellent clustering algorithms, such as pcaReduce [51], SC3 [52], and t-SNE [53], followed by k-means (t-SNE/kms), were used to cluster scRNA-seq data. These methods did not explicitly address the dropout events in scRNA-seq data. Therefore, in model comparison, there were two assumptions: (i) preprocessing of dropout event RNA-seq data by other imputation algorithms, which would improve the accuracy of these clustering methods, and (ii) comparison between scNTImpute and existing splendid imputation algorithms, such as DrImpute [39], CIDR [54], scImpute [21], and MAGIC [37]. scNTImpute performs better in handling dropout events to improve clustering performance (Fig. 12 shows the visualization of evaluation data; see Table 5 for specific data). We can clearly see the experimental comparison of 5 imputation methods and individual imputation methods combined with clustering algorithms. We found that the effect of scNTImpute was significantly better than the clustering enhancement performance of CIDR, followed by the SC3+ DrImpute (Fig. 13).

**Table 5:** Clustering output of multiple imputation methods on real Hrvatin data

	ARI
scNTImpute	0.88
pcaR_M+ DrImpute	0.64
pcaR_M	0.53
pcaR_S+ DrImpute	0.58
pcaR_S	0.54
SC3+ DrImpute	0.76
SC3	0.77
t-SNE/kms+ DrImpute	0.60
t-SNE/kms	0.52
CIDR	0.16
scImpute	0.56
MAGIC	0.45

### Transfer learning across single-cell datasets

**Figure 14:**
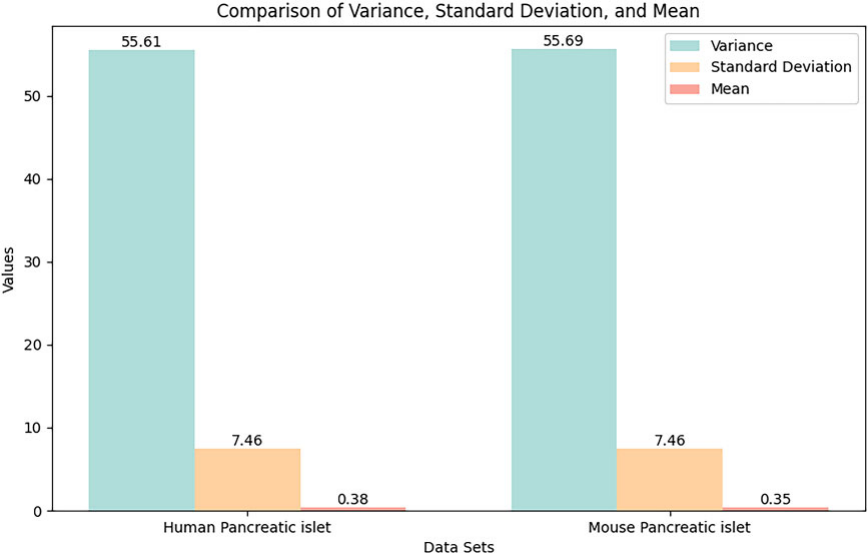
Comparison of similarity indexes (mean, variance, standard deviation) between mouse islet data and human islet data.

A prominent feature of scNTImpute is its parameters, so the knowledge of modeling scRNA-seq data can be transferred across datasets. As part of scNTImpute, the model trained on the reference scRNA-seq dataset can be applied to infer the cell-topic mixture and the parameters of the mixture model for the target scRNA-seq dataset, without ensuring that the two datasets share the same cell type. To illustrate, we employed two real RNA-seq datasets: HP datasets and mouse pancreatic islet (MP) datasets [32] were used to conduct cross-species transfer learning of scNTImpute models. Both datasets were obtained using the inDrop method (a droplet-based single-cell RNA-seq sequencing technique). The assumption of transfer learning is that the distribution of data in the source domain should be similar to the distribution of data in the target domain. Therefore, we primarily demonstrate the similarity of the datasets used in the transfer learning section from two perspectives. First, mean, variance, and standard deviation are commonly used statistical measures to analyze the characteristics and similarities of datasets. If their mean is close and their variance and standard deviation are similar, then their similarity is higher. Conversely, if these measures differ significantly, their similarity is lower. By analyzing the calculations, the results of the evaluation metrics for the two datasets are shown in Table 6. We also visualize the data from Table 6 (Fig. 14), which provides a more intuitive way of observation. We find that their values for all three statistical indicators are very close. Especially in terms of standard deviation, the two datasets have almost the same level of dispersion. Additionally, by comparing the mean, we can see that the central tendencies of the two datasets are also very similar.

**Table 6:** Three metrics used to measure the similarity of the data used for transfer learning

Evaluation index	Variance	Mean	Standard deviation
HP	55.61	0.38	7.46
MP	55.69	0.35	7.46

**Figure 15:**
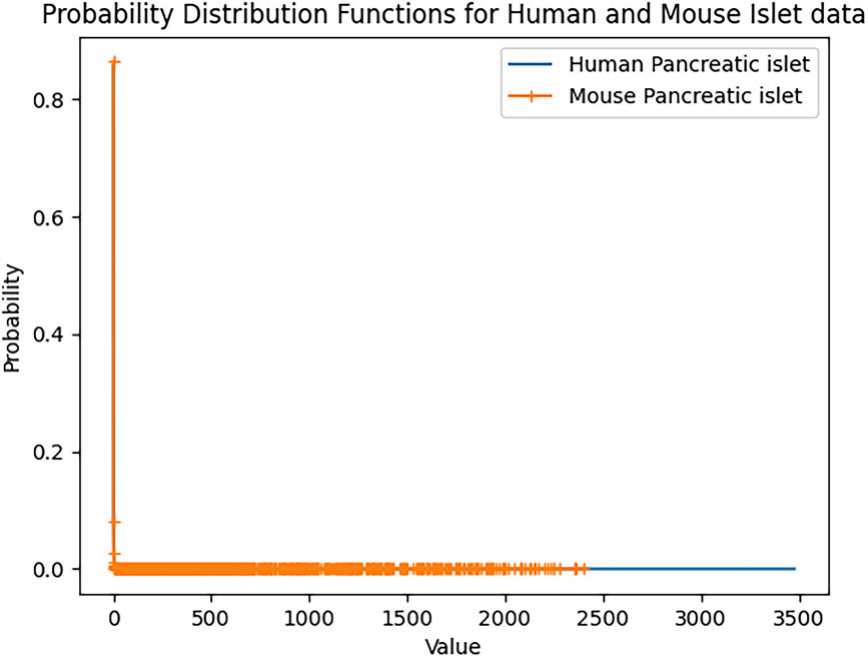
Probability distribution functions for mouse islet data and human islet data.

Second, the probability distribution function is a function used to describe the probability of the possible values of a random variable. It can also help us understand and analyze similarities between datasets. For discrete data such as scRNA-seq datasets, we can calculate the frequency of occurrence for each value and divide these frequencies by the total size of the dataset to obtain the probability of each value. To facilitate the intuitive analysis of the probability distribution functions of the two datasets, we visualized the two probability distributions (Fig. 15). Due to the sparsity of the original scRNA-seq datasets, the frequencies of zero values are relatively high in both datasets. By visualizing the probability distributions, we can easily observe that the distributions of the two datasets are very similar.

**Figure 16:**
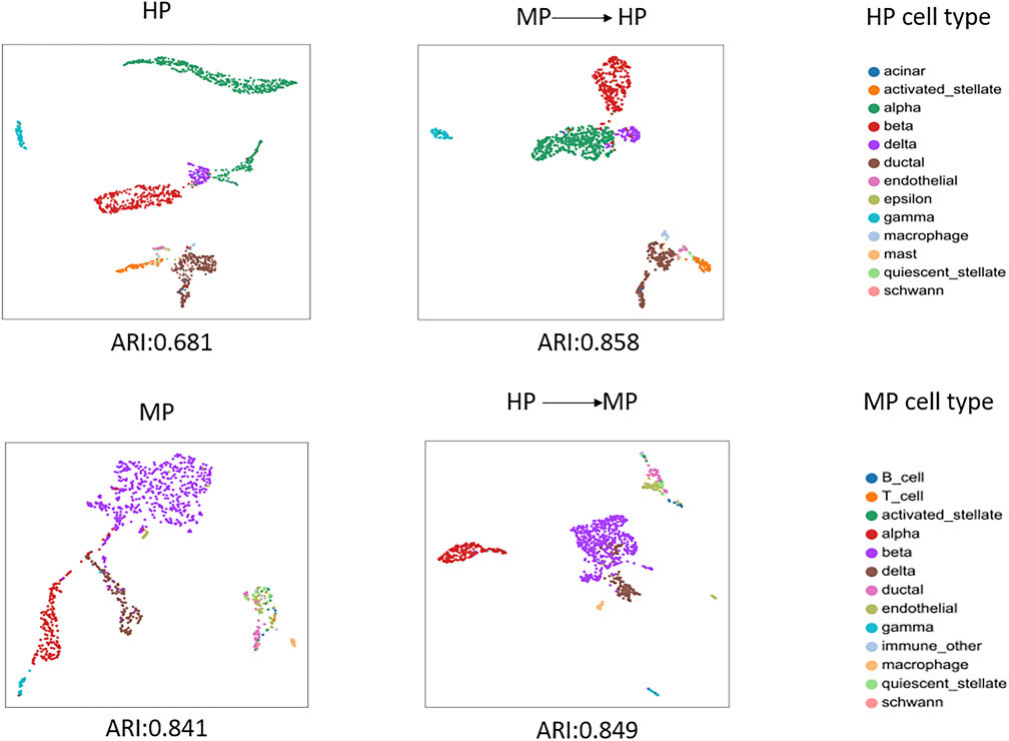
The transfer learning performance of scNTImpute: transfer learning from mouse pancreatic islet data to human pancreatic islet data (MP-HP) and human pancreatic islet data transfer learning to mouse pancreatic islet data (HP-MP).

Next we conducted research on transfer learning. First, if the HP dataset was directly trained on the model, the 4 imputation indicators were ARI, 0.681; NMI, 0.751; RI, 0.884; and MI, 1.429. Second, we trained a scNTImpute model on the MP dataset and used the trained model to impute and evaluate HP data. Ultimately, an exciting transfer learning effect was produced (ARI reached 0.858 in the HP dataset; refer to Table 7 for other specific results). In order to verify the stability of the model transfer learning, we conducted the transfer learning from the HP dataset to the MP dataset, and the results were also surprising. The results of direct imputation and transfer learning imputation of the HP data set were visualized by UMAP (Fig. 16). After transfer, scNTImpute improved many indicators and learned to be cell type-specific (Table 7, Fig. 16). To compare with other methods, we used scNTImpute, scVI-LD, and scVI to evaluate clustering performance in transfer learning tasks. Clustering performance was mainly measured by the ARI between real cell types and Leiden [43] clusters. Overall, scNTImpute obtained the best learning results in cross-species transfer learning between HP and MP (Table 8).

**Table 7:** Using multiple metrics to measure scNTImpute’s transfer learning performance

	HP	MP→HP	MP	HP→MP
ARI	0.681	0.858	0.841	0.849
NMI	0.751	0.821	0.758	0.769
MI	1.429	1.345	1.163	1.232
RI	0.884	0.946	0.931	0.933
CS	0.930	0.8378	0.901	0.863
FMS	0.757	0.894	0.892	0.901

**Table 8:** Transfer learning effectiveness output of different methods

Source dataset	MP	HP
Target dataset	HP	MP
scNTImpute	0.858	0.849
scVI-LD	0.690	0.478
scVI	0.524	0.425

### Path enrichment analysis and statistical significance test of scNTImpute topics

**Figure 17:**
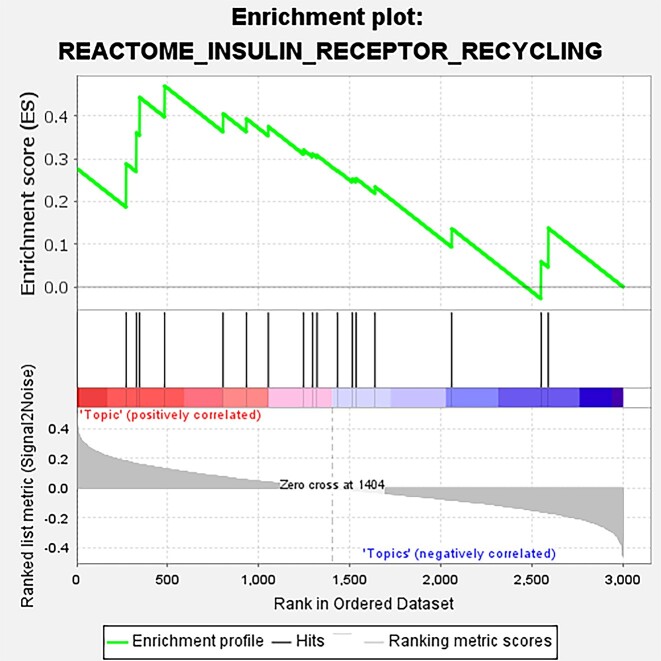
The topic of human islet data is enriched in the insulin receptor recycling pathway.

**Figure 18:**
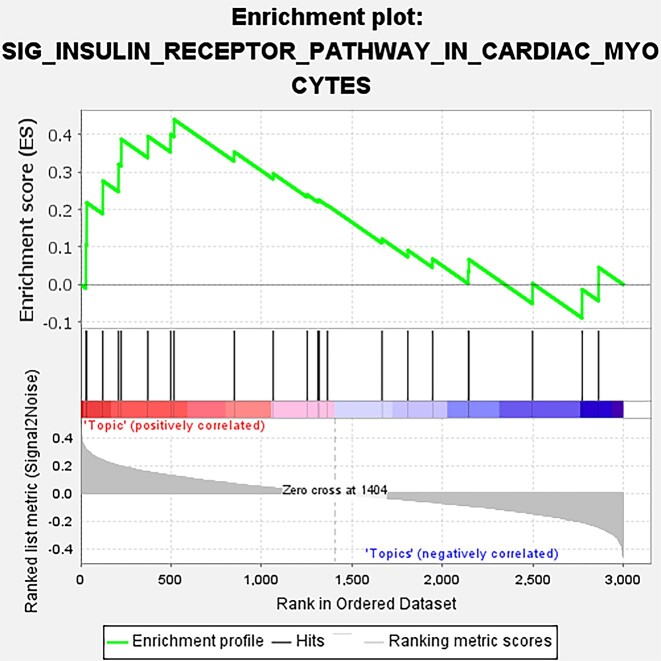
The topic of human islet data is enriched in the cardiac myocyte insulin receptor signaling pathway.

**Figure 19:**
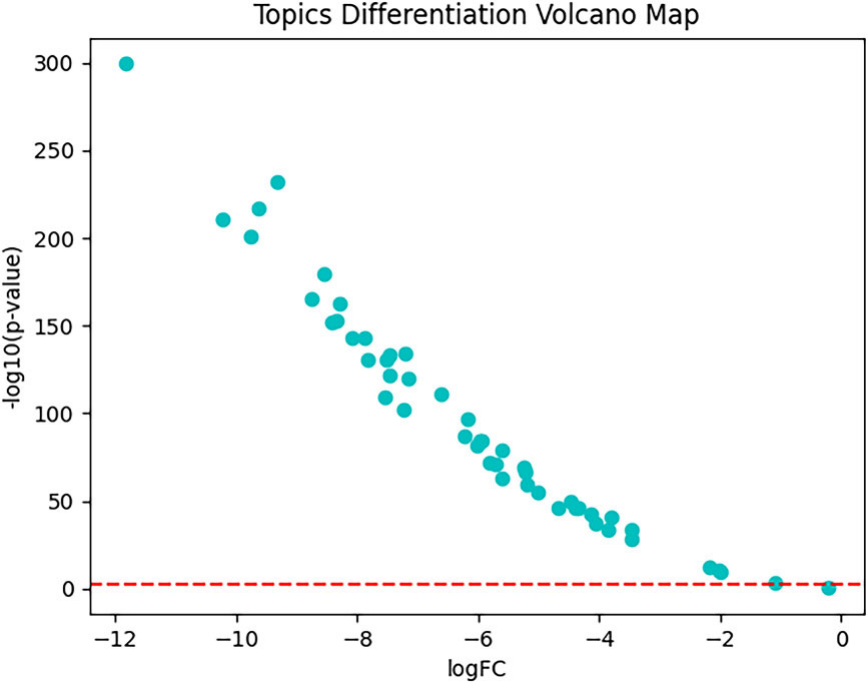
Differences in the topic matter of human islet data.

We next investigated separately whether the topics of scNTImpute were biologically relevant to known human genetic pathways and whether there were differences between topics. First, we employed gene set enrichment analysis [55] for exploration. We trained a scNTImpute with 50 topics using the HP dataset. For the obtained topics, we detected a number of significantly enriched pathways. Several of these were related to pancreatic function, including the insulin receptor recycling, cardiac myocyte insulin receptor signaling pathway, insulin signaling pathways, pancreatic cancer, and so on (Fig. 17, Fig. 18). The set of contained genes between the black bar in the middle and the highest point (ES value) is called the leader subset, which contributed to the upregulation of the entire pathway. Furthermore, based on the differences in the enrichment levels of topics in the pathway, there were also significant differences between topics. We calculated *P* value and fold change (FC), and converted *P* value as a negative logarithm to $- {\log }_{10}( {{\mathrm{p}} - {\mathrm{value}}} )$, while fold change was logarithmically converted to $\log ( {FC} )$. (The red line in Fig. 19 is the threshold line for $\langle i \rangle p\langle {/i} \rangle < 0.01$.) In general, by taking the negative logarithm of the *P* values, most of our topics are smaller than the set threshold, which shows that there are significant differences in our topics (Fig. 19).

### Scalability and efficiency

**Figure 20:**
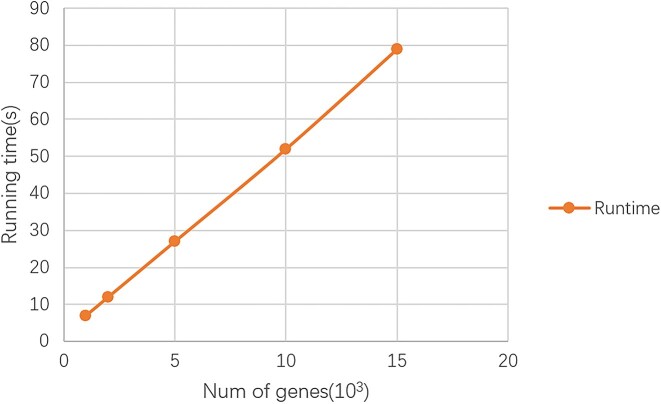
Time complexity analysis of scNTImpute transfer learning.

To validate the scalability and efficiency of the proposed scNTImpute for transferable learning, we tested it on datasets with a different number of genes and recorded the runtime. Specifically, we took the trained model directly for imputation on additional datasets. We performed imputation on datasets containing 1k, 2k, 5k, 10k, and 15k genes and investigated the relationship between running time and the number of genes (Fig. 20). The running time of imputation exhibited a linear increase relative to the number of genes. As scNTImpute is a neural topic-based imputation method, its runtime increases with the number of genes. In practical applications, the number of genes and cells is limited. So, scNTImpute is more suitable for scRNA-seq datasets than other imputation methods.

## Methods

### Workflow

We adopted an imputation workflow based on a neural topic model, implemented using the PyTorch (RRID:SCR_018536) dynamic framework on the backend. Our work is divided into the following steps.

### Data preprocessing

We took as input the scRNA-seq gene count expression matrix *X*, where the rows represented cells and the columns represented genes. Data filtering and quality control were performed as a previous step of data preprocessing, and we eventually wanted to get an imputation matrix with the same dimensionality as the original count matrix. To facilitate the subsequent work, we first normalized each sample (cell) and each gene in the matrix separately to obtain two normalized matrices, ${Y}^C$ (normalized by cell) and ${Y}^G$ (normalized by gene). Then, log10 transformed ${Y}^G$ and pseudo-count 1.01 was added to generate the *Y* matrix [56]:


\begin{eqnarray*}
{Y}_{ij} = lo{g}_{10}\left( {Y_{ij}^G + 1.01} \right);{\mathrm{i}} = 1,2, \ldots ,{\mathrm{I}};{\mathrm{j}} = 1,2, \ldots ,{\mathrm{J}}
\end{eqnarray*}

*I* denotes the number of cells and *J* denotes the number of genes. To avoid infinite values of the parameters in later model training, we added pseudo-counts to it. The advantage of logarithmic transformation is that it can prevent some large observations from having a significant impact, eliminate heteroscedasticity issues, and transform the values into continuity, providing greater flexibility for modeling.

### Topic generation process

Inspired by scETM’s research on single-cell transcriptomics [31], we adopted a neural topic model to model the scRNA-seq data distribution [57]. We treated each cell as a document, and each scRNA-seq read (or UMI) served as a marker in the document. The gene that generates the read count (or UMI) is thought of as a word in a vocabulary. We assumed that each cell could be represented as a mixture of underlying cell types, and they were often referred to as potential topics. In the original LDA model [57], a fixed set of *N*-independent Dirichlet distributions ${\mathrm{\beta }}$ was defined, distributed over a vocabulary of size *M*. Formally, the cell-topic mixture generation process is as follows.

Obtaining the potential topic proportion of cell *C* from a logical normal distribution:


\begin{eqnarray*}
{\delta }_C &\sim& {\boldsymbol{N}}\left( {0,I} \right),{\theta }_C = softmax\left( {{\delta }_C} \right) = \frac{{{\mathrm{exp}}\left( {{\delta }_{c,N}} \right)}}{{\mathop \sum \nolimits_{N = 1}^N {\mathrm{exp}}\left( {{\delta }_{c,N}} \right)}}\\ {\theta }_C &\sim& LN\left( {0,I} \right) \end{eqnarray*}

where $\ {\theta }_C$ is the $1 \times N$ cell-topic mix of cell *C*. To simulate the sparsity of gene expression in each cell, the softmax function was used to regulate the expression of all genes. For this purpose, we obtained a mixture of all the cell topics $\theta $ [31].

To enable the extraction of latent topics from the data, in scNTImpute, we consider the latent cell topic mixture ${\theta }_C$ as the unique latent variable for each cell C. We denoted the posterior distribution of latent variables as ${\mathrm{p}}({\mathrm{\delta }}|{Y}^C)({Y}^C$ (normalized gene expression matrix above). However, directly solving for the true posterior distribution in high-dimensional space is computationally challenging. Therefore, a variational inference method was employed to approximate the true posterior distribution by minimizing the difference between ${\mathrm{q}}( {{\delta }_c} )$ and ${\mathrm{p}}({\mathrm{\delta }}|{Y}^C)$, and the variational posterior was easier to compute compared to the true posterior. Specifically, we defined the following distribution: ${\mathrm{q}}({\mathrm{\delta }}|{\mathrm{y}})$=$\mathop \prod \limits_c q({\delta }_c|{y}_c)$, where $q( {{\delta }_c{\mathrm{|}}{y}_c} ) = {\mu }_c + dia( {{\sigma }_c} ){\boldsymbol{N}}( {0,I} )$. The parameters $[ {{\mu }_c,\log \sigma _c^2} ]$ were estimated by a two-layer feed-forward neural network ${\mathrm{NNET}}( {Y_c^C;{{\mathrm{W}}}_\theta } )$. This network approximated the complete statistics of the cellular topic mixture ${\delta }_c$. To learn the variational parameter ${{\mathrm{W}}}_\theta $ mentioned above, we optimized the evidence lower bound (ELBO) for the logarithmic likelihood [31]. This involved minimizing the Kullback–Leibler (KL) divergence, which measures the difference between the approximate posterior distribution and the true posterior distribution. The goal is to reduce this divergence and bring the approximate posterior closer to the true posterior:


\begin{eqnarray*}
ELBO = E\left[ {\log {\mathrm{p}}\left( {{\mathrm{Y|\theta }}} \right)} \right] - {D}_{KL}[{\mathrm{q}}({\mathrm{\theta }}|{\mathrm{Y}})||{\mathrm{p}}\left( {\mathrm{\theta }} \right)]
\end{eqnarray*}

The first term represents the likelihood function, which is measured using negative log-likelihood. The second term, the reconstruction likelihood, is a regularization term involving the KL divergence between the approximate distribution ($q( {{\delta }_c{\mathrm{|}}{y}_c} ) = N( {{\mu }_c,diag( {{\sigma }_c} )} )$) and the prior distribution (${\mathrm{p}}( {{\delta }_c} ) = N( {0,I} )$), which encourages the variational posterior distribution to approximate the prior distribution. We sampled a few latent variable samples from the reparameterized Gaussian distribution $q( {{\delta }_c{\mathrm{|}}{y}_c} )$, where their mean and variance were determined by the aforementioned NNET (Double layer feedforward neural network for estimating sufficient statistics of the proposed distribution of cell topic mixtures). These samples served as noise estimates for ELBO [31]. Ultimately, the gradients were backpropagated to optimize the weights of the encoder to achieve the goal of extracting topic features.

### Study dropout values

After acquiring the transformed gene expression matrix *Y*, we can infer which genes in the cell were affected by the dropout event. Instead of considering all zero values as dropout values, we used a neural network to systematically determine whether zero values were dropout values. First, the normal distribution described continuous data, while the reads count (gene expression) data were discrete. Second, the reads count data could only take values that were nonnegative integers, and for scRNA-seq data, the most commonly used normal distribution was not reasonable. Certainly, the zero-inflated negative binomial distribution has proven to be a good model for describing scRNA-seq data and serves as the basis for some outstanding models. With the presence of dropout events, most genes have bimodal expression patterns in similar cells. We adapted the mixture model used in scImpute [21]. The similar mixture models have been shown to effectively capture the bimodal features of single-cell gene expression data [32, 56, 45], where the Gamma distribution represents the dropout phenomenon, and the Normal distribution is used for indicating actual gene expression. It is important to note that the transformed gene expression levels are no longer integers, so the widely used read counts obeying a negative binomial distribution are not an appropriate choice. For each gene, the proportions and parameters of the two components may be distinctive in different cell types. As a result, we assume that the expression level of each gene *j* is a random variable ${Y}_j$ following a Gamma–Normal mixed distribution, with a density function of [21]:


(1) \begin{eqnarray*}
{f}_{{Y}_j}\left( y \right) = {{\mathrm{\lambda }}}_j \cdot {\mathrm{Gamma}}\left( {{\mathrm{y}};{{\mathrm{\alpha }}}_j,{{\mathrm{\beta }}}_j} \right) + \left( {1 - {{\mathrm{\lambda }}}_j} \right) \cdot {\mathrm{Normal}}\left( {{\mathrm{y}};{{\mathrm{\mu }}}_j,{{\mathrm{\sigma }}}_j} \right) \end{eqnarray*}

where ${{\mathrm{\lambda }}}_j$ is the dropout rate of genes, the ${{\mathrm{\alpha }}}_j$ and ${{\mathrm{\beta }}}_j$ are the shape and rate parameters in the Gamma distribution, and ${{\mathrm{\mu }}}_j$ and ${{\mathrm{\sigma }}}_j$ are the mean and standard deviation in the normal distribution, respectively. When a sequencing experiment fails to accurately capture the transcriptional expression of genes, the Gamma distribution models the observed gene expression, while the Normal distribution simulates the actual gene expression level. The intuition behind this mixture model is that if a gene has high expression and low variation in multiple cells, the “zero” count expression is more likely to be affected by dropout events; on the other hand, if a gene has consistently low or moderate expression and high variation, then the zero counts may reflect the true biological significance.

After a given distribution of the mixture model, the log-likelihood of each gene at all cell expression levels can be calculated as $l( {{{\mathrm{\lambda }}}_j,{{\mathrm{\alpha }}}_j,{{\mathrm{\beta }}}_j,{{\mathrm{\mu }}}_j,{{\mathrm{\sigma }}}_j} ) = \mathop \sum \limits_{i = 1}^n {\mathrm{log}}{f}_{{Y}_j}( {{y}_{ij};{{\mathrm{\lambda }}}_j,{{\mathrm{\alpha }}}_j,{{\mathrm{\beta }}}_j,{{\mathrm{\mu }}}_j,{{\mathrm{\sigma }}}_j} )$ [56]. The parameters in the model shown in Equation (1) are calculated by a neural network, and these estimates are denoted as ${{{\mathrm{\widetilde \lambda }}}_j}, {{{\mathrm{\widetilde \alpha }}}_j}, {{{\mathrm{\widetilde \beta }}}_j}, {{{\mathrm{\widetilde \mu }}}_j}, {{{\mathrm{\widetilde \sigma }}}_j}$. We can filter the gene expression values based on the undetected probability of the gene in the cell [56], and the dropout rate of gene *j* in cell *i* can be computed as:


(2) \begin{eqnarray*}
{d}_{ij} = \frac{{ {{{\mathrm{\widetilde \lambda }}}_j}{\mathrm{Gamma}}\left( {{Y}_{ij}; {{{\mathrm{\widetilde \alpha }}}_j}, {{{\mathrm{\widetilde \beta }}}_j}} \right)}}{{ {{{\mathrm{\widetilde \lambda }}}_j} \cdot {\mathrm{Gamma}}\left( {{Y}_{ij}; {{{\mathrm{\widetilde \alpha }}}_j}, {{{\mathrm{\widetilde \beta }}}_j}} \right) + \left( {1 - {{{\mathrm{\widetilde \lambda }}}_j}} \right) \cdot {\mathrm{Normal}}\left( {{Y}_{ij}; {{{\mathrm{\widetilde \mu }}}_j}, {{{\mathrm{\widetilde \sigma }}}_j}} \right)}}
\end{eqnarray*}

Because $\ {d}_{ij} \in ( {0,1} )$, a smaller ${d}_{ij}$ indicates that the observed gene expression ${Y}_{ij}$ has higher confidence. We set the threshold *t* by which the dropout rate ${d}_{ij} < t$ is considered an accurate measure with high confidence, and when the dropout rate ${d}_{ij} \ge t$, then gene expression ${Y}_{ij}$ is considered a dropout value.

### Imputation

To impute the dropout values accurately, we needed to borrow expression data of gene *j* in other similar cells that were not affected by dropout to fill in. Specifically, on the basis of the above obtained cell-topic mixture $\theta $, the essence of which was also the dimensionality reduction of scRNA-seq data while effectively reducing the impact of most dropouts in the data, we calculated the similarity matrix of cells, where each element means how similar the cell is to other cells. The degree of similarity of cell *i* and other cells is calculated as follows:


\begin{eqnarray*}
{Z}_{ii^{\prime}} = min\sqrt {\mathop \sum \limits_{i^{\prime} = 1,i = 1}^{I,I} {{\left( {{\theta }_i - {\theta }_{i^{\prime}}} \right)}}^2} {\mathrm{\ }}\left( {i^{\prime},i = 1,2,3 \ldots I} \right) \end{eqnarray*}

where *Z* indicates the degree of similarity between cell *i* and cell $i^{\prime}$. A larger value indicates that two cells are less likely to belong to the same type of cell and less similar, and a smaller value indicates greater similarity (excluding the degree of similarity with itself, i.e., 0 value). In compliance with the similarity between cells, we can borrow the same nondropout gene expression data from similar cell $i^{\prime}$ of cell *i* for the imputation of dropout genes in cell *i*.


\begin{eqnarray*}
{\mathop{X}^{\smallsmile}}_{ij} = {X}_{i^{\prime} j},({d}_{i^{\prime} j} < t) \end{eqnarray*}

### Imputation evaluation

To benchmark the imputation performance, we compared several scRNA-seq data imputation tools that were identical to scNTImpute. We utilized the original dataset for the evaluation experiments. After the imputation of the original data was completed, 4 leading evaluation indicators, ARI, RI, NMI, and MI, were utilized for the evaluation. ARI is interpreted as


\begin{eqnarray*}
ARI = \frac{{RI - E\left[ {RI} \right]}}{{MAX\left( {RI} \right) - E\left[ {RI} \right]}}
\end{eqnarray*}

RI is interpreted as


\begin{eqnarray*}
RI = \frac{{a + b}}{{C_n^2}}
\end{eqnarray*}

where *a* indicates the correct number of markers of cells that should have been of the identical type and after clustering were also in the same type, *b* represents the correct number of markers of cells that were not of the same type and did not cluster to the identical type after clustering, and $C_n^2$ represents the total number of possible pairs. $E[ {RI} ]$ is the expected RI of the random markers [45].

NMI is explained as


\begin{eqnarray*}
NMI\left( {Q,R} \right) = \frac{{2MI\left( {Q,R} \right)}}{{H\left( Q \right) - H\left( R \right)}}
\end{eqnarray*}

MI is interpreted as


\begin{eqnarray*}
MI\left( {Q,R} \right) = \mathop \sum \limits_{i = 1}^{\left| Q \right|} \mathop \sum \limits_{j = 1}^{\left| R \right|} P\left( {i,j} \right)\log \left( {\frac{{P\left( {i,j} \right)}}{{P\left( i \right)P\left( j \right)}}} \right) \end{eqnarray*}

In the above equation, *Q* means the original category of each cell, while *R* indicates the category to which the cells belong after clustering. $H( Q )$ expresses the entropy of *Q*.

## Availability of Source Code and Requirements

Project name: scNTImpute

Project homepage: https://github.com/qiyueyang-7/scNTImpute.git

Research Resource Identifier (#RRID): SCR_024395

BiotoolsID: scNTImpute

Operating system(s): Platform independent

Programming language: Python

Other requirements: conda, Python 3.7, numpy 1.21, pandas 1.3

License: MIT License

## Abbreviations

AE: autoencoder; AMI: adjusted mutual information; ARI: adjusted Rand index; CS: cosine similarity; DCA: deep count autoencoder; ELBO: evidence lower bound; FMS: Fowlkes–Mallows score; GAN: generative adversarial network; HP: human pancreatic islet; KL: Kullback–Leibler; MI: mutual information; MP: mouse pancreatic islet; NMI: normalized mutual information; RI: Rand index; RNA-seq: RNA sequencing; scRNA-seq: single-cell RNA sequencing; VAE: variational autoencoder.

## Authors’ Contributions

Y.Q. and L.L. conceived and developed the study. Y.Q. completed the scNTImput workflow and the writing of the main manuscript text. S.H. and L.T. reviewed and contributed to all versions of the manuscript text. All authors read and approved the final manuscript.

## Competing Interests

The authors declare that there no competitive interests.

## Supplementary Material

giad098_GIGA-D-23-00090_Original_Submission

giad098_GIGA-D-23-00090_Revision_1

giad098_Response_to_Reviewer_Comments_Original_Submission

giad098_Reviewer_1_Report_Original_Submission Ka-Chun Wong -- 6/26/2023 Reviewed

giad098_Reviewer_1_Report_Revision_1 Ka-Chun Wong -- 9/8/2023 Reviewed

giad098_Reviewer_2_Report_Original_Submission Xuejun Liu -- 7/9/2023 Reviewed

giad098_Reviewer_2_Report_Revision_1 Xuejun Liu -- 9/11/2023 Reviewed

## Data Availability

The scRNA-seq data used in this article are all publicly available. All data are available at GEO, with human brain data [33] access number: GSE67835; Chung et al. dataset [34] accession code: GSE75688; Hrvatin dataset [3] GEO accession code: GSE59739; Romanov et al. dataset [50] GEO accession code: GSE74672; Deng et al. dataset [48] GEO accession code: GSE45719; mouse pancreatic islet data [32] GEO accession code: GSE84133. Human pancreatic islet data [49] are available at GEO or EMBL-EBI database with accession codes GSE81076, GSE85241, GSE86469, E-MTAB-5061, and GSE84133. All additional supporting data are available in the *GigaScience* repository, GigaDB [58].
