# Supplementary material for: Imputation method for single-cell RNA-seq data using neural topic model
Source: Gigascience. 2023 Nov 24;12:giad098. doi: 10.1093/gigascience/giad098 (PMC10673642; doi:10.1093/gigascience/giad098)
Supplement: giad098_GIGA-D-23-00090_Original_Submission [file giad098_giga-d-23-00090_original_submission.pdf]

GigaScience
Imputation Methods for Single-Cell RNA-seq Data Using Neural Topic Models
--Manuscript Draft--

**Manuscript Number:**	GIGA-D-23-00090
**Full Title:**	Imputation Methods for Single-Cell RNA-seq Data Using Neural Topic Models
**Article Type:**	Technical Note
**Funding Information:**	
**Abstract:**	Single-cell RNA sequencing (scRNA-seq) technology studies transcriptome and cell-to-cell differences from higher single-cell resolution and different perspectives. Despite the advantage of high capture efficiency, downstream functional analysis of scRNA-seq data is made difficult by the excess of zero values, i.e., the dropout phenomenon. To effectively address this problem, we introduced scNTImpute, an imputation framework based on a neural topic model. A neural network encoder is used to extract underlying topic features of single-cell transcriptome data to infer high-quality cell similarity. At the same time, we determine which transcriptome data are affected by the dropout phenomenon according to the learning of the mixture model by the neural network. On the basis of stable cell similarity, the same gene information in other similar cells is borrowed to impute only the missing expression values. By evaluating the performance of real data, scNTImpute can accurately and efficiently identify the dropout values and imputes them accurately. In the meantime, the clustering of cell subsets is improved and the original biological information in cell clustering is solved which is covered by technical noise.Single-cell RNA sequencing (scRNA-seq) technology studies transcriptome and cell-to-cell differences from higher single-cell resolution and different perspectives. Despite the advantage of high capture efficiency, downstream functional analysis of scRNA-seq data is made difficult by the excess of zero values, i.e., the dropout phenomenon. To effectively address this problem, we introduced scNTImpute, an imputation framework based on a neural topic model. A neural network encoder is used to extract underlying topic features of single-cell transcriptome data to infer high-quality cell similarity. At the same time, we determine which transcriptome data are affected by the dropout phenomenon according to the learning of the mixture model by the neural network. On the basis of stable cell similarity, the same gene information in other similar cells is borrowed to impute only the missing expression values. By evaluating the performance of real data, scNTImpute can accurately and efficiently identify the dropout values and imputes them accurately. In the meantime, the clustering of cell subsets is improved and the original biological information in cell clustering is solved which is covered by technical noise.
**Corresponding Author:**	Lin Liu<br>Yunnan Normal University<br>Kunming, CHINA
**Corresponding Author Secondary Information:**	
**Corresponding Author's Institution:**	Yunnan Normal University
**Corresponding Author's Secondary Institution:**	
**First Author:**	Yueyang Qi
**First Author Secondary Information:**	
**Order of Authors:**	Yueyang Qi
	Shuangkai Han
	Ling Tang
	Lin Liu
**Order of Authors Secondary Information:**	

Powered by Editorial Manager® and ProduXion Manager® from Aries Systems Corporation

Additional Information:	
**Question**	**Response**
Are you submitting this manuscript to a special series or article collection?	No
**Experimental design and statistics**<br><br>Full details of the experimental design and statistical methods used should be given in the Methods section, as detailed in our Minimum Standards Reporting Checklist. Information essential to interpreting the data presented should be made available in the figure legends.<br><br>Have you included all the information requested in your manuscript?	Yes
**Resources**<br><br>A description of all resources used, including antibodies, cell lines, animals and software tools, with enough information to allow them to be uniquely identified, should be included in the Methods section. Authors are strongly encouraged to cite Research Resource Identifiers (RRIDs) for antibodies, model organisms and tools, where possible.<br><br>Have you included the information requested as detailed in our Minimum Standards Reporting Checklist?	Yes
**Availability of data and materials**<br><br>All datasets and code on which the conclusions of the paper rely must be either included in your submission or deposited in publicly available repositories (where available and ethically appropriate), referencing such data using a unique identifier in the references and in the "Availability of Data and Materials" section of your manuscript.	Yes

Powered by Editorial Manager® and ProduXion Manager® from Aries Systems Corporation

Have you have met the above requirement as detailed in our Minimum Standards Reporting Checklist?	

Powered by Editorial Manager® and ProduXion Manager® from Aries Systems Corporation

Manuscript

Click here to access/download;Manuscript;original manuscript.docx

Imputation Methods for Single-Cell RNA-seq Data Using Neural Topic Models

Yueyang Qi[1], Shuangkai Han[2], Ling Tang[3], Lin Liu[*]
[1]Yunnan Normal University, Kunming, 650500, China, E-mail: qyy18848869532@163.com.
[2]Yunnan Normal University, Kunming, 650500, China , E-mail: han_skai@163.com.
[3]Yunnan Normal University, Kunming, 650500, China , E-mail: maitanweng2@163.com.
[*]Correspondence address. Lin Liu is with the Yunnan Normal University, Kunming 650500. E-mail: liulinrachel@163.com.

Abstract

Single-cell RNA sequencing (scRNA-seq) technology studies transcriptome and cell-to-cell differences from higher single-cell resolution and different perspectives. Despite the advantage of high capture efficiency, downstream functional analysis of scRNA-seq data is made difficult by the excess of zero values, i.e., the dropout phenomenon. To effectively address this problem, we introduced scNTImpute, an imputation framework based on a neural topic model. A neural network encoder is used to extract underlying topic features of single-cell transcriptome data to infer high-quality cell similarity. At the same time, we determine which transcriptome data are affected by the dropout phenomenon according to the learning of the mixture model by the neural network. On the basis of stable cell similarity, the same gene information in other similar cells is borrowed to impute

only the missing expression values. By evaluating the performance of real data, scNTImpute can accurately and efficiently identify the dropout values and imputes them accurately. In the meantime, the clustering of cell subsets is improved and the original biological information in cell clustering is solved which is covered by technical noise.

Introduction

Bulk-cell RNA-seq techniques have been widely used for transcriptome analysis to study transcriptional structure, splicing patterns, and expression levels of genes and transcriptomes [1]. To address biological issues such as cell heterogeneity and gene expression randomness, it is particularly important to interpret cell-specific transcriptome landscapes [2]. Although the bulk-cell RNA-seq technique is popular, it measures the average expression level of genes in batch cells, and the expression of variable genes will be pulled to average. Therefore, it is not possible to study cell specificity based on transcriptomics. Fortunately, by studying gene expression status in single cells, scRNA-seq technology overcomes the shortcomings of traditional batch cell sequencing technology and is becoming a powerful tool to capture the inter-cell variability of the transcriptome. It has dramatically changed the study of transcriptomics, helping us to

decode life from a higher resolution and spatiotemporal structure, accurately reflecting the heterogeneity between cells. The study of scRNA-seq data has become a hot subject today.

Currently, we use multiple scRNA-seq platforms, the two most popular being Fluidigm and Drop-Seq. The Drop-Seq processes thousands of cells in a single run, which not just saves time and cost, but also is simple to operate. Fluidigm, while it usually processes fewer cells, has higher coverage rates. So, an increasing number of studies are using these techniques to discover new cell types [3,4], new markers for specific cell types [3,5,6], and cell heterogeneity [6,7,8,9,10,11].

However, scRNA-seq technology has its corresponding drawbacks. ScRNA-seq data have a relatively higher noise level than batch cell RNA-seq data, resulting in a major problem that is the sparsity of the gene expression matrix, i.e., the data often exhibits a large number of zero values. Most of these zeros are artificially caused by defects in sequencing techniques, including, but not limited to, inadequate gene expression, low capture rates and sequencing depth, or other technical factors. As a result, the observed zero value does not reflect the underlying true expression level [12]. This gene expression bias may be further increased during subsequent amplification steps. Thus, dropout events can significantly affect downstream bioinformatics analysis. At present, researchers have proposed a

variety of imputation models through different principles and methods. These research results have a great guiding role in scRNA-seq data integration, enrichment analysis, etc. According to the design characteristics of the imputation algorithm, the data feature learning and processing methods, we roughly divide the RNA-seq data imputation methods into two categories: deep learning-based imputation method and non-deep learning imputation method.

In the traditional non-deep learning interpolation algorithm, because of its simple idea, it is able to usually fit the corresponding statistical probability model or use the expression matrix for smoothing and diffusion. So， there are certain advantages in some specific types of samples. Florian Wagner et al. used the KNN-smoothing method by finding k-nearest neighbors between cells and aggregating gene-specific UMI counts to impute the gene expression matrix. In finding the number of nearest neighbors k, instead of using a way to fit a certain model, the data's imputation is achieved stepwise by constructing a partially smoothed profile with a variance-stabilizing transformation [13]. Li et al. introduced a statistical method, scImpute [14], which uses a mixture model to learn the loss probability of each gene in each cell. By setting a loss probability threshold, the input data is divided into two parts: the set of genes severely affected by ″dropout″ A_j and the set of unaffected genes B_j. Eventually,

the information on similar cells is learned from B_j for imputation. ScImpute automatically identifies possible dropout values and performs imputation only on these values without introducing new biases to the rest of the data. Nancy R. Zhang et al. proposed SVAER algorithm, which is a method that uses information across genes and cells to impute zero values so as to optimize the expression of all genes. By looking for potential relationships between genes, the true expression level of each gene in each cell can be restored, eliminating technical differences. Nevertheless, SVAER alters all gene expression levels, including those not affected by dropout events, which could introduce new biases into the data and potentially eliminate biologically significant variation [15]. For scRNA-seq data that are large, often high-dimensional, sparse, and complex, analysis using traditional computational methods becomes difficult and infeasible.

As deep neural network algorithms have gained great application in biomedical fields in recent years, they mine complex relationships within single-cell data through a series of basic hierarchical operations. The typical deep learning algorithms applied to scRNA-seq data are Autoencoders (AE), Variational Auto-Encoders (VAE), Generative Adversarial Networks (GANs), and other models. Fabian J.Theis et al. proposed the Deep Count Autoencoder Network (DCA) model by improving the conventional autoencoder. The reconstruction error is

defined as the probability of the noise model distribution rather than the reconstruction of the input data itself. Gene specific distribution parameters are learned by minimizing reconstruction errors in an unsupervised manner. The noise model is eventually applied to sparse count data, giving it a loss function specifically for scRNA-seq data. Meanwhile, its deep learning framework is capable of capturing the complexity and nonlinearity of scRNA-seq data and is highly scalable [16]. Lana X. Garmire et al. proposed a deep neural network based-imputation algorithm (DeepImputes) by constructing multiple sub neural-networks, which imputes genes in a divide-and-conquer manner, not only achieving the highest overall accuracy but also providing faster computing time and requiring less memory [17]. Xu et al. proposed a scRNA-seq data imputation method (scIGANs) founded on generative adversarial network. The method uses networks to generate cells rather than cells observed in the original matrix to balance the performance between dominant and rare cell populations. Enabling it to learn nonlinear gene-to-gene dependencies from complex samples of multicellular types and train generative models to generate realistic expression profiles of defined cell types. After training, K-Nearest Neighbors (KNN) is used to impute the same type of cells, thereby eliminating technical variations without damaging inter-cell biological variability. This method is robust to small data with low

expression or inter cell differences [18]

Because most downstream analyses of scRNA-seq, such as differential gene expression analysis, cell-type specific gene identification, and new cell type definition, rely on the accuracy of gene expression measurements. Therefore, it is particularly important to correct the expression of "false zero values" caused by dropout events in scRNA-seq data through accurate and robust imputation methods. [14]. These imputation methods identify the dropout values in scRNA-seq data from different perspectives and impute them. However, for non-deep learning, it is impossible to effectively learn the feature relationship of some complex nonlinear data, and it does not have good flexibility and expansibility. The architecture of deep learning itself is a 'black box', with many learning layers and thousands of nodes, making the underlying features learned and the full rich potential of the single-cell dataset unleashed uninterpretable.

Despite the study of RNA-Seq data is an active area of research, accurate recovery of single-cell gene expression data remains a great challenge. Inspired by neural topic, we design an accurate and stable imputation method, called scNTImpute, that can more precisely impute gene expression affected by dropout. Specifically, scNTImpute performs deep feature extraction and the construction of networks of encoders through the coding learning mechanism of transferable neural networks.

Learning network parameters and highly interpretable mixtures of cell-topic from scRNA-seq data. Topic features can be used to learn the similarity of cells, and researchers are capable of performing topic pathway enrichment analysis on them at a later stage. This is used to explore whether they have relevance to currently known gene pathways, as well to uncover topics that may be condition-specific or cell type-specific to improve the interpretability of deep feature from a biological perspective. Concurrently, we will get underlying connections such as cell-to-cell, cell-to-gene, or gene-to-gene in single-cell data. The flexibility of the neural topic model makes it excellent for processing scRNA-seq data. Besides, scNTImport uses neural networks to learn the mixture model parameters of gene expression distribution, solving the dropout probability of each gene in each cell. This allows us to more directly understand the true state of the expression data of the scRNA-seq transcriptome and distinguish which gene transcripts are affected by dropout. Using information about the same gene in other similar cells to impute the dropout value in a cell through underlying cell-gene connections. Prior to this, make sure that the borrowed information is selected for genes that are as free as possible from dropout events.

Results

scNTImpute model overview

We propose a new scRNA-seq data imputation method on account of a neural topic model. Adapted from the single-cell embedded topic model (scETM) and inherits the advantages of the topic model. It not only makes the features more robust but is very effective in handling heavy-tailed and large distributions of word frequencies [19,37]. For the analysis of the scRNA-seq data study, we pass the sampled cell and transcriptome expressions separately as vectors of normalized counts to two fully connected neural networks (i.e., two-layer fully connected encoders). Firstly, using a fully connected neural network encoder, we infer the topic mixing ratio of cells, namely, the cell-topic mixture (Figure 1a). Secondly, we use the second neural network to infer the mixed distribution parameters of the transcriptome and obtain probability estimates of whether the gene expression value in each cell is dropout value by using the mixed distribution model. Finally, the cell-topic mixture infers similar cells of the cell in which the dropout gene is located and use the same genetic information from similar cells for the imputation of dropout values (Figure 1b).

Figure 1: Overview of the scNTImpute workflow. **a** scNTImpute uses a neural-topic network architecture to model the single-cell

transcriptome. Normalized counts of the gene expression data matrix and its transpose matrix for each single-cell dataset are used as input to the encoder. The encoder network generates random samples of potential cell-topic mixtures (θ_d, cells d=1, ..., N) that can be used to compute inter-cell similarity. Neural networks learn the parameters of a mixture model of gene expression data and can be used to identify dropout values. **b**, Imputation works using similar cell information. A cell similarity matrix is generated by calculating the intercellular similarity from the resulting mixture of cell-topic. In view of the learned parameters of the mixture model, the dropout value is identified and imputed with the information of similar cells (cell j) of the cell where the dropout value is located (cell d). **c**, Transfer learning workflow. The scNTImpute model trained on the reference scRNA-seq dataset can infer the mixture of cell-topic θ and the mixture model distributions from the unseen scRNA-seq dataset and perform accurate imputation on the unseen dataset. The scRNA-seq dataset is visualized by UMAP and evaluates by standard unsupervised clustering metrics using real cell types.

scNTImpute can efficiently impute scRNA-seq data

Recovery of biologically significant gene expression from dropout events is the primary goal of scRNA-seq imputation, which can further

reduce the impact on downstream analysis. In order to accurately evaluate the imputation performance of different models, we use published real data sets for experiments (including human brain single-cell datasets [20], Chung [21]). scNTImpute stably provides competitive results. To intuitively see the imputation performance between models, four indexes are adopted as the benchmark (i.e., ARI-Adjusted Rand Index, RI-Rand Index, NMI-Normalized Mutual Information, and MI-Mutual Information). To be specific, we used scNTImpute and several other advanced imputation methods to evaluate real human brain scRNA-seq datasets (i.e., SCRABBLE [22], DCA [16], MAGIC [23], DeepImpute [17], scIGANs(w/) [18], AutoImpute [24], DrImpute [25], ENHANCE [26], SAVER [15], scGAIN [28], scImpute [14], VIPER [29], scIGANs(w/o) [18]). By visualizing the evaluation results (Figure 2), we can intuitively see that the values of the four imputation evaluation indicators of scNTImpute are relatively high (specific imputation comparison results are shown in Table 1). After imputation, we used Leiden [38] clustering and UMAP visualization for the complete scNA-Seq data （Figure 3）. The results show that scNTImport accurately and effectively recovers biologically significant gene expression from single-cell datasets.

	NMI	ARI	MI	RI
scNTImpute	0.722	0.709	1.313	0.906
SCRABBLE	0.126	0.083	0.225	0.480

DCA	0.496	0.328	0.886	0.780
MAGIC	0.615	0.390	1.169	0.812
DeepImpute	0.568	0.364	1.057	0.802
scIGANs(w/)	0.540	0.364	0.999	0.802
AutoImpute	0.666	0.559	1.366	0.876
DrImpute	0.642	0.471	1.189	0.833
ENHANCE	0.706	0.538	1.359	0.862
SAVER	0.602	0.575	1.076	0.856
scGAIN	0.138	0.092	0.246	0.626
scImpute	0.672	0.545	1.223	0.857
VIPER	0.544	0.306	0.972	0.972
scIGANs(w/o)	0.349	0.243	0.631	0.631

Table 1

We perform imputation experiments on another published real dataset, Chung [21]. The above imputation indexes are not the only criteria for evaluating the imputation of RNA-SEQ data. Different from the above, the other two imputation indexes are used for evaluation (Cosine Similarity (CS), Fowlkes-Mallows Score (FMS)). Similarly, we compare it with several other existing excellent imputation models. The evaluation results are visualized (Figure 4), from which we can see that our model performs the best in both cosine similarity and fowlkes-mallows scores. Especially in FMS, a large gap is drawn with other

imputation methods. (The specific imputation comparison results are shown in Table 2, and Figure 5 shows the clustering effect on the complete Chung data set).

	CS	FMS
scNTImpute	0.5394	0.8264
Magic	0.4890	0.5493
DCA	0.3280	0.4080
DeepImpute	0.2668	0.4392
SAUCIE	0.4762	0.5372
scIGANs	0.5048	0.5961
scImpute	0.4413	0.5531
SCVI	0.2071	0.2833

Table 2

scNTImpute improves the clustering of cell subpopulations

To test the ability of scNTImport to improve cell type or cell subgroup clustering, we applied scNTImport to real scRNA-seq datasets, i.e., also on the Chung [21] dataset. In addition to reusing the above ARI and NMI evaluation indexes, we also adopt another commonly used clustering index AMI (Adjusted Mutual Information). We impute scRNA-seq data with scNTImpute and other different imputation models, and compare cell clustering with complete imputation data. Through the

comparison of evaluation data (refer to Table 3 for specific data, Figure 6: visualization of comparative data), our imputation method is the highest in ARI index and has relatively significant and stable performance in AMI and NMI clustering index (Figure 7 shows the clustering effect after imputation).

	ARI	AMI	NMI
scNTImpute	0.6403	0.5071	0.5093
Magic	0.3851	0.6195	0.6304
DCA	0.2362	0.4150	0.4327
DeepImpute	0.2625	0.3071	0.3225
SAUCIE	0.3706	0.6053	0.6165
scIGANs	0.4511	0.6102	0.6199
scImpute	0.3967	0.4986	0.5069
SCVI	0.1078	0.2463	0.2679

Table 3

Moreover, we evaluated the clustering effect of scRNA-seq data after imputation on another Hrvatin real data set [30]. We use the cell type stated in the original publication as the basic fact and ARI as a performance indicator. Unlike the previous comparison, here we combine the scNTImport with other developed imputation models and clustering methods to evaluate; that is, before using the clustering algorithm,

use other imputation models to process and compare the results with our model. Several excellent clustering algorithms, such as pcaReduce [31], SC3 [32], and t-SNE [33] followed by k-means (t-SNE/kms), are used to cluster scRNA-seq data. These methods do not explicitly address the dropout events in scRNA-seq data. Therefore, in model comparison, there are two assumptions: (1) Preprocessing of dropout event RNA-Seq data by other imputation algorithms will improve the accuracy of these clustering methods, and (2) Comparison between scNTImport and existing splendid imputation algorithms. Existing scRNA-seq imputation tools such as DrImpute [25], CIDR [35], scImpute [14], and MAGIC [23]. ScNTImport performs better in handling dropout events to improve clustering performance (Figure 8 shows the visualization of evaluation data, see Table 4 for specific data). We can clearly see the experimental comparison of five imputation methods and individual imputation methods combined with clustering algorithms. We found that the effect of scNTImpute was significantly better than the clustering enhancement performance of CIDR, followed by the SC3+ DrImpute (Figure 9. Clustering after Hrvatin [30] imputation using scNTImport).

	ARI
scNTImpute	0.88
pcaR_M+ DrImpute	0.64
pcaR_M	0.53

pcaR_S+ DrImpute	0.58
pcaR_S	0.54
SC3+ DrImpute	0.76
SC3	0.77
t-SNE/kms+ DrImpute	0.60
t-SNE/kms	0.52
CIDR	0.16
scImpute	0.56
MAGIC	0.45

Table 4

Transfer learning across single-cell datasets

A prominent feature of scNTImport is its parameters, so the knowledge of modeling scRNA-seq data can be transferred across datasets. As part of scNTImport, the model trained on the reference scRNA-seq dataset can be applied to infer the cell-topic mixture and the parameters of the mixture model for the target scRNA-seq dataset, without ensuring that the two datasets share the same cell type. To illustrate, we employ two real RNA-Seq data sets: Human Pancreatic islet data sets (HP) [39] and Mouse Pancreatic islet data sets (MP) [40] were used to conduct cross-species transfer learning of scNTImpute models. Both datasets were obtained using the inDrop method (a droplet-based single

cell RNA-Seq sequencing technique). Firstly, if the HP dataset is directly trained on the model, the four imputation indicators are ARI: 0.681, NMI: 0.751, RI: 0.884, and MI: 1.429. Secondly, we train a scNTImport model on the MP dataset and use the trained model to impute and evaluate HP data. Ultimately, an exciting transfer learning effect was produced (ARI reached 0.858 in the HP dataset, refer to Table 5 for other specific results). In order to verify the stability of the model transfer learning, we conducted the transfer learning from the HP dataset to the MP dataset, and the results were also surprising. The results of direct imputation and transfer learning imputation of the HP data set were visualized by UMAP (Figure 10. Clustering of four imputation results: direct imputation on HP, scNTInput trained on MP to impute HP, direct imputation on MP, and scNTInput trained on HP to impute MP). After transfer, scNTImpute improved many indicators and learned cell type-specific (Table 5, Figure 10). To compare with other methods, we use scNTImport, scVI-LD, and scVI to evaluate clustering performance in transfer learning tasks. Clustering performance is mainly measured by the Adjusted Rand index (ARI) between real cell types and Leiden [38] clusters. Overall, scNTImpute obtains the best learning results in cross-species transfer learning between HP and MP (Table 6).

	HP	MP→HP	MP	HP→MP

ARI	0.681	0.858	0.841	0.849
NMI	0.751	0.821	0.758	0.769
MI	1.429	1.345	1.163	1.232
RI	0.884	0.946	0.931	0.933
CS	0.930	0.8378	0.901	0.863
FMS	0.757	0.894	0.892	0.901

Table 5

Source dataset	MP	HP
Target dataset	HP	MP
scNTImpute	0.858	0.849
scVI-LD	0.690	0.478
scVI	0.524	0.425

Table 6

Methods

Workflow

We adopt an imputation workflow based on a neural topic model, implemented using the PyTorch dynamic framework on the backend. Our work is divided into the following steps.

Data preprocessing

We take as input the scRNA-seq gene count expression matrix X, where the rows represent cells and the columns represent genes. Data filtering and quality control are performed as a previous step of data preprocessing, and we eventually want to get an imputation matrix with the same dimensionality as the original count matrix. To facilitate the subsequent work, we first normalize each sample (cell) and each gene in the matrix separately to obtain two normalized matrices, Y^C (normalized by cell) and Y^G (normalized by gene). Then log10 transforms Y^G and add pseudo-count 1.01 to generate the Y matrix [41]:

$$Y_{ij} = log_{10}\left(Y_{ij}^G + 1.01\right); \ \mathrm{i} = 1,2, \ldots, \mathrm{I}; \mathrm{j} = 1,2, \ldots, \mathrm{J}$$

I denotes the number of cells and J denotes the number of genes. To avoid infinite values of the parameters in later model training, we added pseudo-counts to it. The advantage of logarithmic transformation is that it can prevent some large observations from having a significant impact, eliminate heteroscedasticity issues, and transform the values into continuity, providing greater flexibility for modeling.

Topic generation process

We adopted a neural topic model to model the scRNA-seq data distribution [36,19]. We treat each cell as a document, and each scRNA-seq read (or UMI) serves as a marker in the document. The gene that

generates the read count (or UMI) is thought of as a word in a vocabulary [19]. We assume that each cell can be represented as a mixture of underlying cell types, and they are often referred to as potential topics. The original LDA model [36] a fixed set of N-independent Dirichlet distributions β is defined, distributed over a vocabulary of size M. Formally, the cell-topic mixture generation process is as follows.

Obtaining the potential topic proportion of cell C from a logical normal distribution:

$$\delta_C \sim N(0, I), \theta_C = softmax(\delta_C) = \frac{\exp(\delta_C, N)}{\sum_{N=1}^{N} \exp(\delta_C, N)}$$

$$\theta_C \sim LN(0, I)$$

Where θ_C is the $1 \times N$ cell-topic mix of cell C. To simulate the sparsity of gene expression in each cell, the softmax function is used to regulate the expression of all genes. For this purpose, we obtain a mixture of all the cell topics θ [19].

Study dropout values

After acquiring the transformed gene expression matrix Y, we can infer which genes in the cell are affected by the dropout event. Instead of considering all zero values as dropout values, we use a neural network to systematically determine whether zero values are dropout values. Firstly, the normal distribution describes continuous data, while the

reads count (gene expression) data is discrete. Secondly, the reads count data can only take values that are non-negative integers, and for scRNA-seq data, the most commonly used normal distribution is not reasonable. Certainly, the zero-inflated negative binomial (ZINB) distribution has proven to be a good model for describing scRNA-seq data, and serves as the basis for some outstanding models. With the presence of dropout events, most genes have bimodal expression patterns in similar cells. We adapted the mixture model used in scImpute [14]. The similar mixture models have been shown to effectively capture the bimodal features of single-cell gene expression data [39,40,41]. Where the Gamma distribution represents the dropout phenomenon, and the Normal distribution is used for indicating actual gene expression. It is important to note that the transformed gene expression levels are no longer integers, so the widely used read counts obeying a negative binomial distribution is not an appropriate choice. For each gene, the proportions and parameters of the two components may distinctive in different cell types. As a result, we assume that the expression level of each gene j is a random variable Y_j following a Gamma-Normal mixed distribution, with a density function of [14]:

$$f_{Y_j}(y) = \lambda_j \cdot \mathrm{Gamma}(y; \alpha_j, \beta_j) + (1 - \lambda_j) \cdot \mathrm{Normal}(y; \mu_j, \sigma_j) \quad (1)$$

Where λ_j is the dropout rate of genes, the α_j and β_j are the shape and rate parameters in the gamma distribution, and μ_j and σ_j are the mean

and standard deviation in the normal distribution， respectively. When a sequencing experiment fails to accurately capture the transcriptional expression of genes, the gamma distribution models the observed gene expression, while the normal distribution simulates the actual gene expression level. The intuition behind this mixture model is that if a gene has high expression and low variation in multiple cells, the ″zero″ count expression is more likely to be affected by dropout events; on the other hand, if a gene has consistently low or moderate expression and high variation, then the zero counts may reflect the true biological significance.

After a given distribution of the mixture model, the log-likelihood of each gene at all cell expression levels can be calculated as $l(\lambda_j, \alpha_j, \beta_j, \mu_j, \sigma_j) = \sum_{i=1}^{n} \log f_{Y_j}(y_{ij}; \lambda_j, \alpha_j, \beta_j, \mu_j, \sigma_j)$ [41]. The parameters in the model shown in Equation (1) are calculated by a neural network and these estimates are denoted as $\widetilde{\lambda_j}, \widetilde{\alpha_j}, \widetilde{\beta_j}, \widetilde{\mu_j}, \widetilde{\sigma_j}$. We can filter the gene expression values based on the undetected probability of the gene in the cell [41], and the dropout rate of gene j in cell i can be computed as:

$$d_{ij} = \frac{\widetilde{\lambda_j}\mathrm{Gamma}(Y_{ij}; \widetilde{\alpha_j}, \widetilde{\beta_j})}{\widetilde{\lambda_j} \cdot \mathrm{Gamma}(Y_{ij}; \widetilde{\alpha_j}, \widetilde{\beta_j}) + (1 - \widetilde{\lambda_j}) \cdot \mathrm{Normal}(Y_{ij}; \widetilde{\mu_j}, \widetilde{\sigma_j})} \quad (2)$$

Because $d_{ij} \in$ （0，1）， a smaller d_{ij} indicates that the observed gene expression Y_{ij} has higher confidence. We set the threshold t by which

the dropout rate $d_{ij} < t$ is considered to be an accurate measure with high confidence, and when the dropout rate $d_{ij} \geq t$, then gene expression Y_{ij} is considered a dropout value.

Imputation

To impute the dropout values accurately, we need to borrow expression data of gene j in other similar cells that are not affected by dropout to fill in. Specifically, on the basic of the above obtained cell-topic mixture θ, the essence of which is also the dimensionality reduction of scRNA-seq data while effectively reducing the impact of most dropouts in the data. We calculate the similarity matrix of cells, where each element means how similar the cell is to other cells. The degree of similarity of cell i and other cells is calculated as follows:

$$Z_{ii'} = min\sqrt{\sum_{i'=1,i=1}^{I,I} (\theta_i - \theta_{i'})^2} \qquad (i', i = 1,2,3 \dots I)$$

Where Z indicates the degree of similarity between cell i and cell i'. A larger value indicates that two cells are less likely to belong to the same type of cell and less similar, and a smaller value indicates greater similarity (excluding the degree of similarity with itself, i.e., 0 value). In compliance with the similarity between cells, we can borrow the same non-dropout gene expression data from similar cell i' of cell i for the imputation of dropout genes in cell i.

$$\check{X}_{ij} = X_{i'j}, (d_{i'j} < t)$$

Imputation evaluation

To benchmark the imputation performance, we compared several scRNA-seq data imputation tools that are identical to scNTImpute. We utilized the original data set for the evaluation experiments. After the imputation of the original data was completed, four leading evaluation indicators, ARI (Adjusted Rand Index), RI (Rand index), NMI (Normalized Mutual Information), and MI (Mutual Information), were utilized for the evaluation. Where ARI is interpreted as.

$$ARI = \frac{RI - E[RI]}{MAX(RI) - E[RI]}$$

The RI is interpreted as.

$$RI = \frac{a + b}{C_n^2}$$

Where, a indicates the correct number of markers that cells should have been of the identical type and after clustering are also in the same type; b represents the correct number of markers that cells are not of the same type and do not cluster to the identical type after clustering. C_n^2 represents the total number of possible pairs. And $E[RI]$ is the expected RI of the random markers [42].

NMI is explained as

$$NMI(Q, R) = \frac{2MI(Q, R)}{H(Q) - H(R)}$$

MI is interpreted as.

$$MI(Q,R)=\sum_{i=1}^{|Q|}\sum_{j=1}^{|R|}P(i,j)\log(\frac{P(i,j)}{P(i)P(j)})$$

In the above equation, Q means the original category of each cell, while R indicates the category to which the cells belong after clustering. $H(Q)$ expresses the entropy of Q.

Availability of Source Code and Requirements

Project name: scNTImpute

Project homepage: we open-source the code and host it in a GitHub repository (https://github.com/qiyueyang-7/scNTImpute.git)

Operating system(s): Platform independent

Programming language: Python

Other requirements: conda, Python 3.7, numpy 1.21, pandas 1.3

License: MIT License

Data Availability

The scRNA-seq data used in this manuscript are all publicly available. All data are available at GEO, with human brain data [20] access number: GSE67835; Chuang dataset [21] accession code: GSE75688, Hrvatin dataset [30] GEO accession code: GSE59739. Human Pancreatic Islet data [39] are available at GEO, or EMBL-EBI database with accession codes GSE81076, GSE85241, GSE86469, E-MTAB-5061 and GSE84133, Mouse Pancreatic Islet data [40] GEO accession codes are GSE84133.

Abbreviations

scRNA-seq: Single cell RNA sequencing; ARI: Adjusted Rand Index; RI: Rand Index; NMI: Normalized Mutual Information; MI; Mutual Information; CS: Cosine Similarity; FMS: Fowlkes-Mallows Score; AMI: Adjusted Mutual Information;

Competing Interests

The authors declare that there no competitive interests.

Authors' Contributions

Qi and Liu conceived and developed the study. Qi completed the scNTImput workflow and the writing of the main manuscript text. Han and Tang reviewed and contributed to all versions of the manuscript text. All authors read and approved the final manuscript.

Acknowledgement

This work was supported by the National Natural Science Foundation of China (No.61862067), the Applied Basic Research Project in Yunnan Province (No.202201AT070042) and the NSFC- Yunnan Union Key Grant (No.U1902201)

References

[1] Wang, Z., Gerstein, M. & Snyder, M. RNA-Seq: a revolutionary tool for transcriptomics. Nat Rev Genet 10, 57-63 (2009). https://doi.org/10.1038/nrg2484.

[2] Andrew McDavid, Greg Finak, Pratip K. Chattopadyay, Maria Dominguez, Laurie Lamoreaux, Steven S. Ma, Mario Roederer, Raphael Gottardo, Data exploration, quality control and testing in single-cell qPCR-based gene expression experiments, Bioinformatics, Volume 29, Issue 4, February 2013, Pages 461-467, https://doi.org/10.1093/bioinformatics/bts714.

[3] Usoskin, D., Furlan, A., Islam, S. et al. Unbias

ed classification of sensory neuron types by large-scale single-cell RNA sequencing. Nat Neurosci 18, 145-153 (2015). https://doi.org/10.1038/nn.3881.

[4] Villani AC, Satija R, Reynolds G, Sarkizova S, Shekhar K, Fletcher J, Griesbeck M, Butler A, Zheng S, Lazo S, Jardine L, Dixon D, Stephenson E, Nilsson E, Grundberg I, McDonald D, Filby A, Li W, De Jager PL, Rozenblatt-Rosen O, Lane AA, Haniffa M, Regev A, Hacohen N. Single-cell RNA-seq reveals new types of human blood dendritic cells, monocytes, and progenitors. science. 2017 Apr 21;356(6335):eaah4573. doi: 10.1126/science.aah4573. pmid: 28428369; PMCID: PMC5775029.

[5] Zeisel A, Muñoz-Manchado AB, Codeluppi S, Lönnerberg P, La Manno G, Juréus A, Marques S, Munguba H, He L, Betsholtz C, Rolny C, Castelo-Branco G, Hjerling-Leffler J, Linnarsson S. Brain structure. cell types in the mouse cortex and hippocampus revealed by single-cell RNA-seq. science. 2015 Mar 6; 347(6226):1138-42. doi: 10.1126/science.aaa1934. epub 2015 Feb 19. pmid: 25700174.

[6] Jaitin DA, Kenigsberg E, Keren-Shaul H, Elefant N, Paul F, Zaretsky I, Mildner A, Cohen N, Jung S, Tanay A, Amit I. Massively parallel single-cell RNA- seq for marker-free decomposition of tissues into cell types. science. 2014 Feb 14;343(6172):776-9. doi: 10.1126/science.1247651. pmid: 24531970; PMCID: PMC4412462.

[7] Pollen AA, Nowakowski TJ, Shuga J, Wang X, Leyrat AA, Lui JH, Li N, Szpankowski L, Fowler B, Chen P, Ramalingam N, Sun G, Thu M, Norris M, Lebofsky R, Toppani D, Kemp DW 2nd, Wong M, Clerkson B, Jones BN, Wu S, Knutsson L, Alvarado B, Wang J, Weaver LS, May AP, Jones RC, Unger MA, Kriegstein AR, West JA. Low- coverage single-cell mRNA sequencing reveals cellular heterogeneity and activated signaling pathways in developing cerebral cortex. Nat Biotechnol . 2014 Oct;32(10):1053-8. doi: 10.1038/nbt.2967. epub 2014 Aug 3. PMID: 25086649; PMCID: PMC4191988.

[8] Treutlein B, Brownfield DG, Wu AR, Neff NF, Mantalas GL, Espinoza FH, et al. Reconstructing lineage hierarchies of the distal lung epithelium using single-cell RNA-seq. Nature. 2014;509:371 Nature Publishing Group.

[9] Tirosh I, Venteicher AS, Hebert C, Escalante LE, Patel AP, Yizhak K, et al. Single-cell RNA-seq supports a developmental hierarchy in human oligodendroglioma. Nature. 2016;539:309 Nature Publishing Group.

[10] Shalek AK, Satija R, Adiconis X, Gertner RS, Gaublomme JT, Raychowdhury R, et al. Single-cell transcriptomics reveals bimodality in expression and splicing in immune cells. Nature. 2013;498:236 Nature Publishing Group.

[11] Tang F, Barbacioru C, Bao S, Lee C, Nordman E, Wang X, et al. Tracing the derivation of embryonic stem cells from the inner cell mass by single-cell RNA- Cell Stem Cell. 2010;6:468-78 Elsevier.

[12] van Dijk D, Sharma R, Nainys J, Yim K, Kathail P, Carr AJ, Burdziak C, Moon KR, Chaffer CL, Pattabiraman D, Bierie B, Mazutis L, Wolf G, Krishnaswamy S, Pe 'er D. Recovering Gene Interactions from Single-Cell Data Using Data Diffusion. cell. 2018 Jul 26;174(3):716-729.e27.

doi: 10.1016/j.cell.2018. 05.061. epub 2018 Jun 28. PMID: 29961576; PMCID: PMC6771278.

[13] Wagner, F., Yan, Y., & Yanai, I. (2017). K-nearest neighbor smoothing for high-through put single-cell RNA-Seq data. bioRxiv.

[14] Li, W.V., Li, J.J. An accurate and robust imputation method scImpute for single-cell RNA-seq data. Nat Commun 9, 997 (2018).

[15] Huang M, Wang J, Torre E, Dueck H, Shaffer S, Bonasio R, Murray JI, Raj A, Li M, Zhang NR.SAVER: gene expression recovery for single-cell RNA sequencing . Nat Methods. 2018 Jul;15(7):539-542.

[16] Eraslan, G., Simon, L.M., Mircea, M. et al. Single-cell RNA-seq denoising using a deep count autoencoder. Nat Commun 10, 390 (2019).

[17] Arisdakessian, C., Poirion, O., Yunits, B. et al. DeepImpute: an accurate, fast, and scalable deep neural network method to impute single-cell RNA- seq data. Genome Biol 20, 211 (2019). https://doi.org/10.1186/s13059-019-1837-6

[18] Yungang Xu, Zhigang Zhang, Lei You, Jiajia Liu, Zhiwei Fan, Xiaobo Zhou, scIGANs: single-cell RNA-seq imputation using generative adversarial networks, Nucleic Acids Research, Volume 48, Issue 15, 04 September 2020, Page e85, https://doi.org/10.1093/nar/gkaa506.

[19] Zhao, Y., Cai, H., Zhang, Z. et al. Learning interpretable cellular and gene signature embeddings from single-cell transcriptomic data. Nat Commun 12, 5261 (2021). https://doi.org/10.1038/s41467-021-25534-2.

[20] Darmanis S, Sloan SA, Zhang Y, Enge M, Caneda C, Shuer LM, Hayden Gephart MG, Barres BA, Quake SR. A survey of human brain transcriptome diversity at the single cell level. Proc Natl Acad Sci U S A. 2015 Jun 9;112(23):7285-90. doi: 10.1073/pnas.1507125112. epub 2015

May 18. pmid: 26060301; pmcid: PMC4466750.

[21] Chung W, Eum HH, Lee HO, Lee KM, Lee HB, Kim KT, Ryu HS, Kim S, Lee JE, Park YH, Kan Z, Han W, Park WY. Single-cell RNA-seq enables comprehensive tumour and immune cell profiling in primary breast cancer. Nat Commun. 2017 May 5;8:15081. doi: 10.1038/ncomms15081. PMID: 28474673; PMCID: PMC5424158.

[22] Peng T, Zhu Q, Yin P, Tan K. SCRABBLE: single-cell RNA-seq imputation constrained by bulk RNA-seq data. genome Biol. 2019 May 6;20(1):88. doi: 10.1186/s13059-019-1681-8. PMID: 31060596; PMCID: PMC6501316.

[23] David, van, Dijk., Juozas, Nainys., Roshan, Sharma., Pooja, Kathail., Ambrose, J., Carr., Kevin, R., Moon., Linas, Mazutis., Guy, Wolf., Smita, Krishnaswamy., Dana, Pe'er.(2017). MAGIC: A diffusion-based imputation method reveals gene-gene interactions in single-cell RNA-sequencing data. bioRxiv, 111591-. doi: 10.1101/111591

[24] Talwar, D., Mongia, A., Sengupta, D. et al. AutoImpute: Autoencoder based imputation of single-cell RNA-seq data. Sci Rep 8, 16329 (2018). https://doi.org/10.1038/s41598-018-34688-x.

[25] Gong W, Kwak IY, Pota P, Koyano-Nakagawa N, Garry DJ. DrImpute: imputing dropout events in single cell RNA sequencing data. bmc Bioinformatics. 2018 Jun 8;19(1):220. doi: 10.1186/s12859-018-2226-y. PMID: 29884114; PMCID: PMC5994079.

[26] Wagner, Florian, Dalia Barkley and Itai Yanai. "Accurate denoising of single-cell RNA-Seq data using unbiased principal component analysis." *bioRxiv* (2019): n. pag.

[27] Huang M, Wang J, Torre E, Dueck H, Shaffer S, Bonasio R, Murray JI, Raj A, Li M, Zhang NR. SAVER: gene expression recovery for single-cell RNA sequencing . Nat Methods. 2018

Jul;15(7):539-542. doi: 10.1038/s41592-018-0033-z. Epub 2018 Jun 25. PMID: 29941873; PMCID: PMC6030502.

[28] Gunady M.K., Kancherla J., Bravo H.C., Feizi S. scGAIN: single cell RNA-seq data imputation using generative adversarial networks. 2019; bioRxiv doi:12 November 2019, preprint: not peer reviewedhttps://doi.org/10.1101/837302.

[29] Chen, M., Zhou, X. VIPER: variability-preserving imputation for accurate gene expression recovery in single-cell RNA sequencing studies. Genome Biol 19, 196 (2018). https://doi.org/10.1186/s13059-018-1575-1

[30] Usoskin D, Furlan A, Islam S, Abdo H, Lönnerberg P, Lou D, Hjerling-Leffler J, Haeggström J, Kharchenko O, Kharchenko PV, Linnarsson S, Ernfors P. Unbiased classification of sensory neuron types by large-scale single-cell RNA sequencing. Nat Neurosci. 2015 Jan;18(1):145-53. doi: 10.1038/ nn.3881. epub 2014 Nov 24. pmid: 25420068.

[31] Žurauskienė J, Yau C. pcaReduce: hierarchical clustering of single cell transcriptional profiles. bmc Bioinformatics. 2016 Mar 22;17:140. doi: 10.1186/s12859-016-0984-y. PMID: 27005807; PMCID: PMC4802652.

[32] Kiselev VY, Kirschner K, Schaub MT, Andrews T, Yiu A, Chandra T, Natarajan KN, Reik W, Barahona M, Green AR, Hemberg M. SC3: consensus clustering of single-cell RNA-seq data. Nat Methods. 2017 May;14(5):483-486. doi: 10.1038/nmeth.4236. epub 2017 Mar 27. PMID: 28346451; PMCID: PMC5410170.

[33] Maaten, L.V., & Hinton, G.E. (2008). Visualizing Data using t-SNE. *journal of Machine Learning Research, 9*, 2579-2605.

[34] Gong, W., Kwak, IY., Pota, P. et al. DrImpute: imputing dropout events in single cell RNA

sequencing data. BMC Bioinformatics 19, 220 (2018). https://doi.org/10.1186/s12859-018-2226-y

[35] Lin, P., Troup, M. & Ho, J.W. CIDR: Ultrafast and accurate clustering through imputation for single-cell RNA-seq data. Genome Biol 18, 59 (2017). https://doi.org/10.1186/s13059-017-1188-0

[36] David M. Blei, Andrew Y. Ng, and Michael I. Jordan. 2003. Latent dirichlet allocation. J. Mach. Learn. Res. 3, null (3/1/2003), 993- 1022.

[37] Adji B. Dieng, Francisco J. R. Ruiz, David M. Blei; Topic Modeling in Embedding Spaces. transactions of the Association for Computational Linguistics 2020; 8 439-453. doi: https://doi.org/10.1162/tacl_a_00325

[38] Traag, V., Waltman, L. & Eck, N. From Louvain to Leiden: guaranteeing well-connected communities. Scientifific Reports. 9 pp. 5233 (2019,3)

[39] Stuart, T. et al. Comprehensive integration of single-cell data. cell 177, 1888-1902 (2019).

[40] Baron, M. et al. A single-cell transcriptomic map of the human and mouse pancreas reveals inter-and intra-cell population structure. Cell Systems 3, 346–360 (2016).

[41] Wei Vivian Li, Yanzeng Li, scLink: Inferring Sparse Gene Co-expression Networks from Single-cell Expression Data, Genomics, Proteomics & Bioinformatics, Volume 19, Issue 3, 2021, Pages 475-492, ISSN 1672-0229, https://doi.org/10.1016/j.gpb.2020.11.006.

[42] Wang, J., Ma, A., Chang, Y. et al. scGNN is a novel graph neural network framework for single-cell RNA-Seq analyses. Nat Commun 12, 1882 (2021). https://doi.org/10.1038/s41467-021-22197-x.

[43] Kharchenko, P., Silberstein, L. & Scadden, D. Bayesian approach to single-cell differential

expression analysis. Nat Methods 11, 740–742 (2014). https://doi.org/10.1038/nmeth.2967

[44] Kharchenko, P., Silberstein, L. & Scadden, D. Bayesian approach to single-cell differential expression analysis. Nat Methods 11, 740–742 (2014). https://doi.org/10.1038/nmeth.2967.

[45] Li WV, Li JJ. A statistical simulator scDesign for rational scRNA-seq experimental design. Bioinformatics. 2019 Jul 15;35(14):i41-i50. doi: 10.1093/bioinformatics/btz321. PMID: 31510652; PMCID: PMC6612870.

[46] Vu TN, Wills QF, Kalari KR, Niu N, Wang L, Rantalainen M, Pawitan Y. Beta-Poisson model for single-cell RNA-seq data analyses. Bioinformatics. 2016 Jul 15;32(14):2128-35. doi: 10.1093/bioinformatics/btw202. Epub 2016 Apr 19. PMID: 27153638.

[47] Backenroth, D. et al. FUN-LDA: a latent dirichlet allocation model for predicting tissue-specific functional effects of noncoding variation: methods and applications. Am. J. Human Genet. 102, 920–942 (2018).

[48] Zhang L, Zhang S. Comparison of computational methods for imputing single-cell RNA-sequencing data. IEEE/ACM transactions on computational biology and bioinformatics.2018.

[49] Wu X, Zhou Y. GE-Impute: graph embedding-based imputation for single-cell RNA-seq data. Brief Bioinform. 2022 Sep 20;23(5):bbac313. doi: 10.1093/bib/bbac313. PMID: 35901457.

[50] Huang Z, Wang J, Lu X, Mohd Zain A, Yu G. scGGAN: single-cell RNA-seq imputation by graph-based generative adversarial network. Brief Bioinform. 2023 Feb 2:bbad040. doi: 10.1093/bib/bbad040. Epub ahead of print. PMID: 36733262.

[51] Huang M, Ye X, Li H, Sakurai T. Missing Value Imputation With Low-Rank Matrix Completion in Single-Cell RNA-Seq Data by Considering Cell Heterogeneity. Front Genet. 2022

Jul 14;13:952649. doi: 10.3389/fgene.2022.952649. PMID: 35910201; PMCID: PMC9329700.

[52] Zhang L, Zhang S. Imputing single-cell RNA-seq data by considering cell heterogeneity and prior expression of dropouts. J Mol Cell Biol. 2021 Apr 10;13(1):29-40. doi: 10.1093/jmcb/mjaa052. PMID: 33002136; PMCID: PMC8035992.

[53] Tian T, Min MR, Wei Z. Model-based autoencoders for imputing discrete single-cell RNA-seq data. Methods. 2021 Aug;192:112-119. doi: 10.1016/j.ymeth.2020.09.010. Epub 2020 Sep 22. PMID: 32971193; PMCID: PMC8592282.

[54] Lu F, Lin Y, Yuan C, Zhang XF, Ou-Yang L. EnTSSR: A Weighted Ensemble Learning Method to Impute Single-Cell RNA Sequencing Data. IEEE/ACM Trans Comput Biol Bioinform. 2021 Nov-Dec;18(6):2781-2787. doi: 10.1109/TCBB.2021.3110850. Epub 2021 Dec 8. PMID: 34495837.

[55] Zhu M, Lai Y. Improvements Achieved by Multiple Imputation for Single-Cell RNA-Seq Data in Clustering Analysis and Differential Expression Analysis. J Comput Biol. 2022 Jul;29(7):634-649. doi: 10.1089/cmb.2021.0597. Epub 2022 May 16. PMID: 35575729.

Figure Click here to access/download;Figure;Figure .pdf

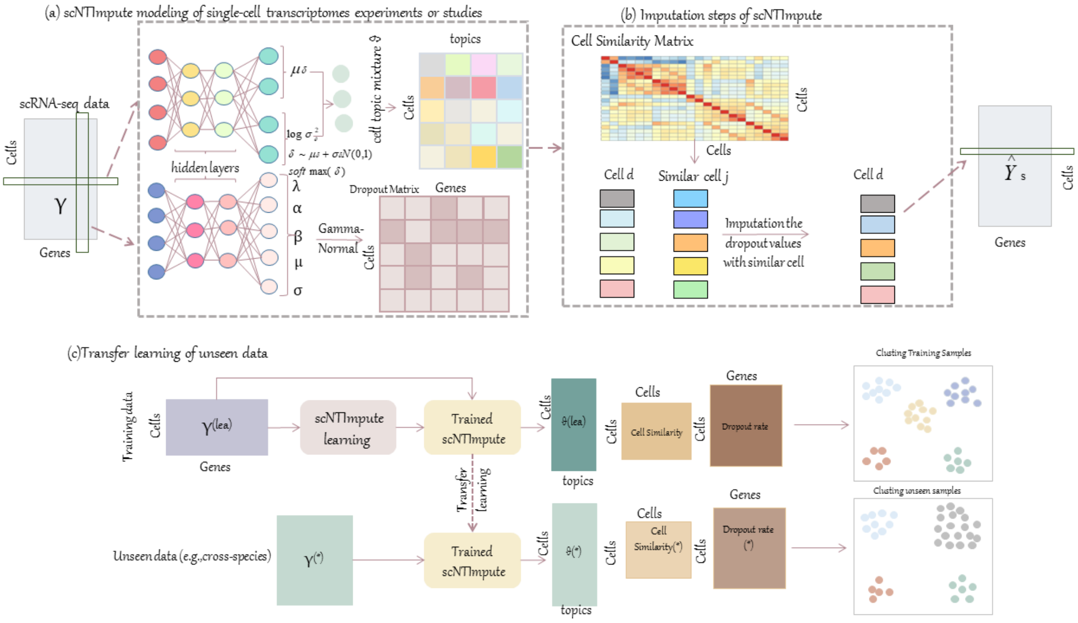

Figure 1

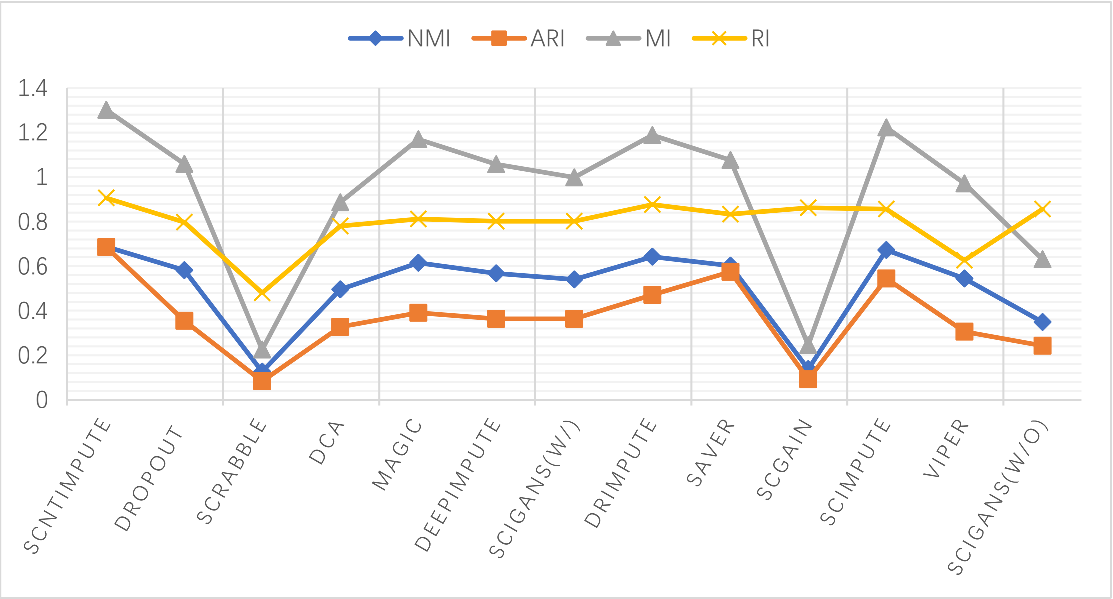

Figure 2

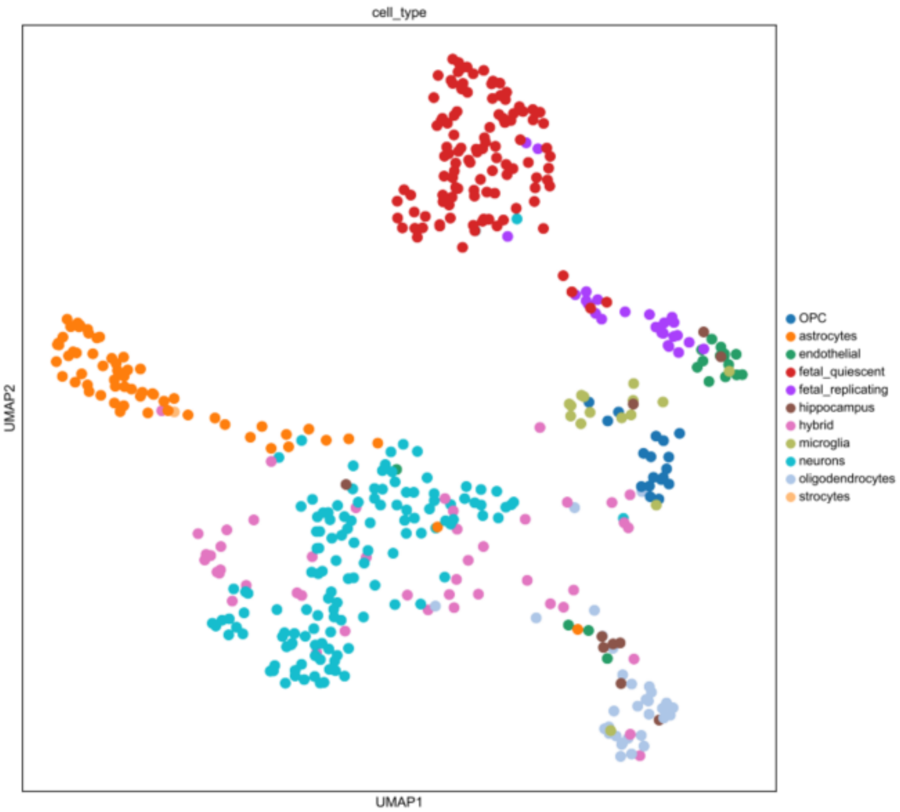

Figure 3

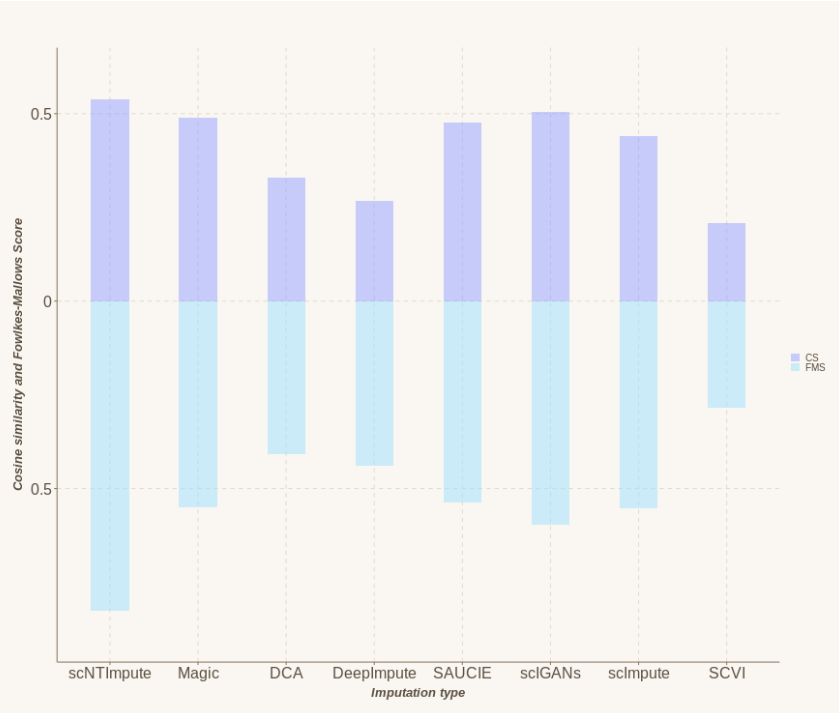

Figure 4

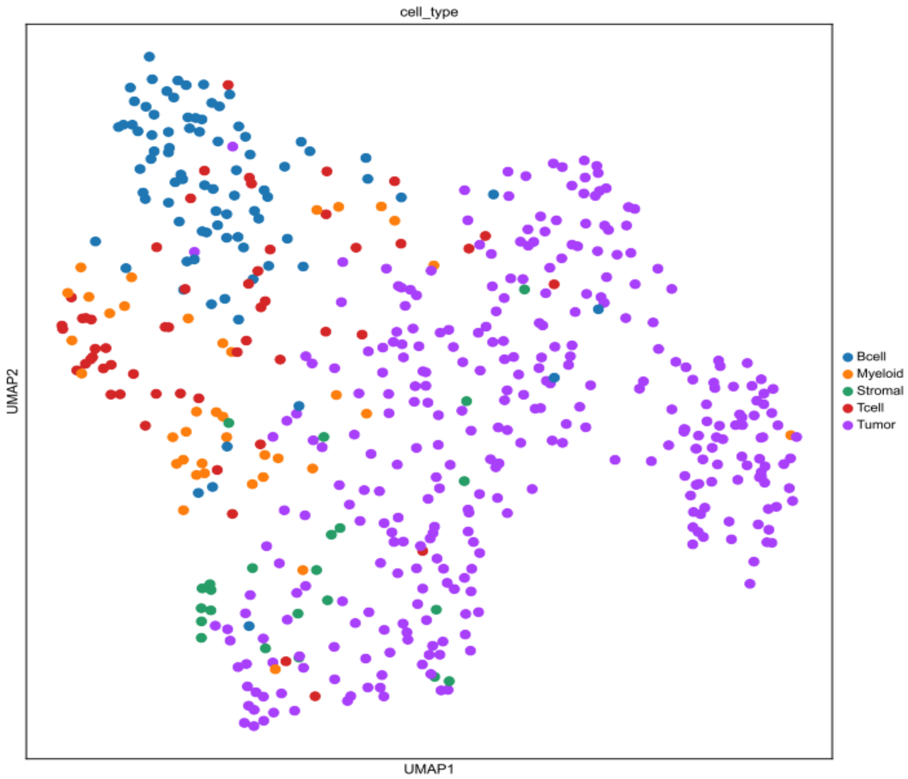

Figure 5

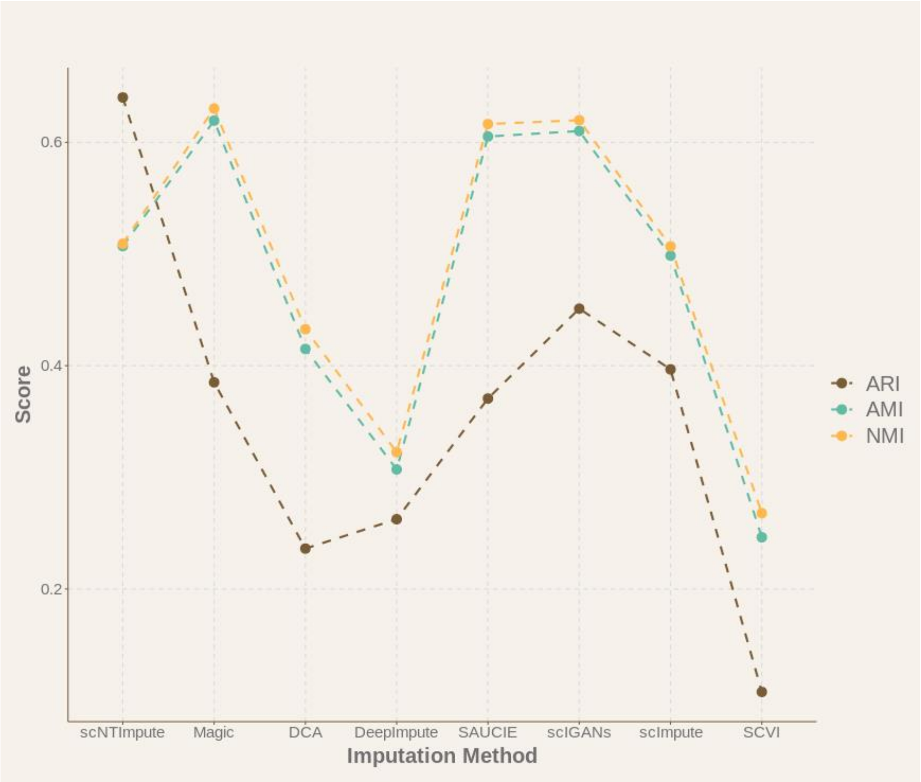

Figure 6

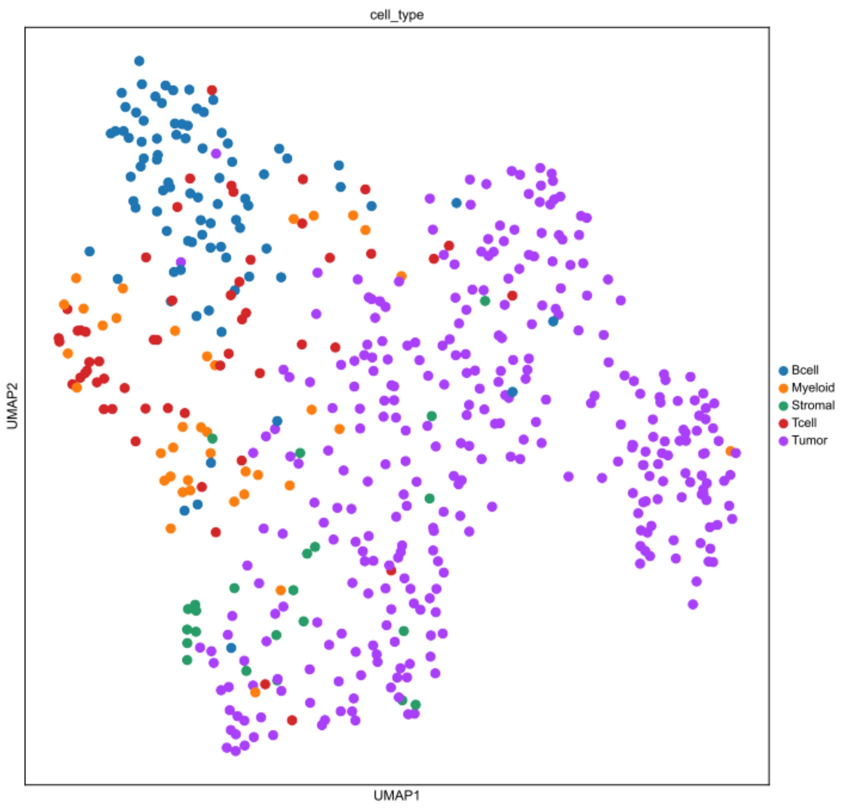

Figure 7

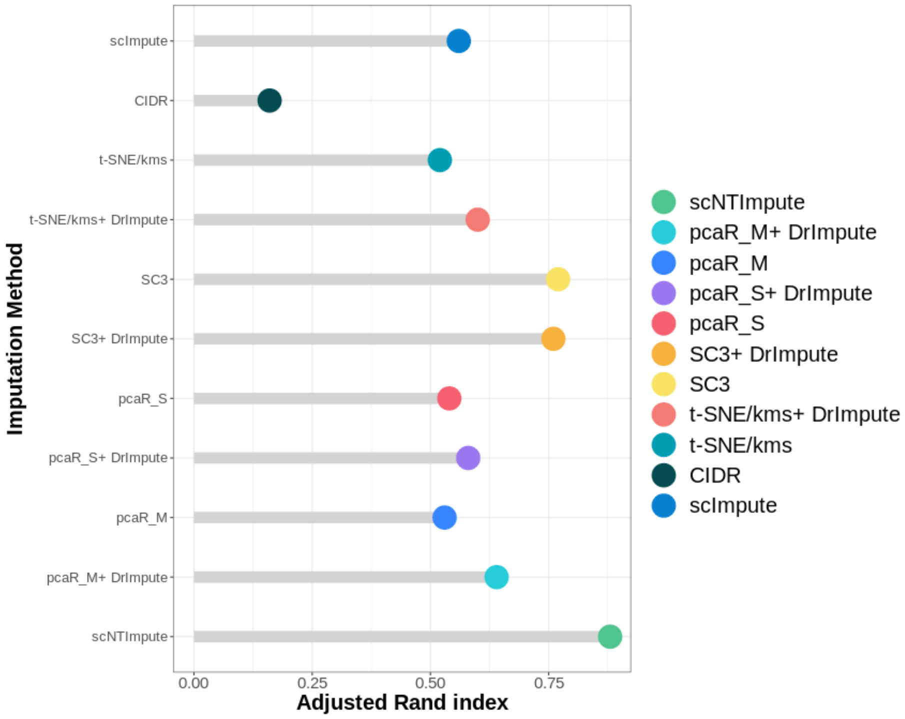

Figure 8

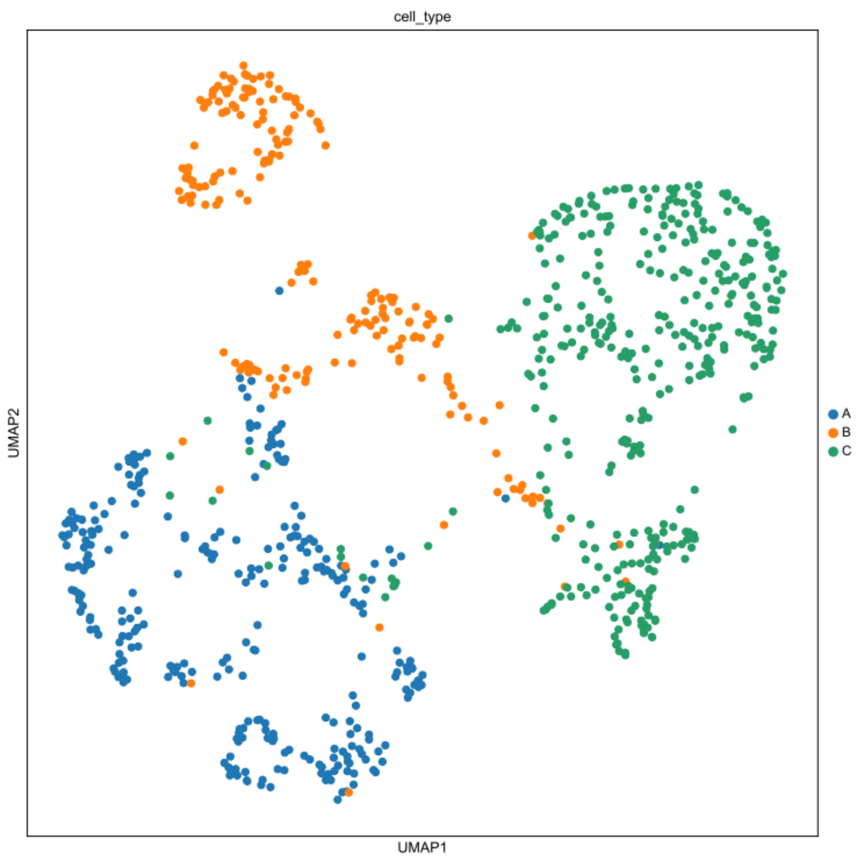

Figure 9

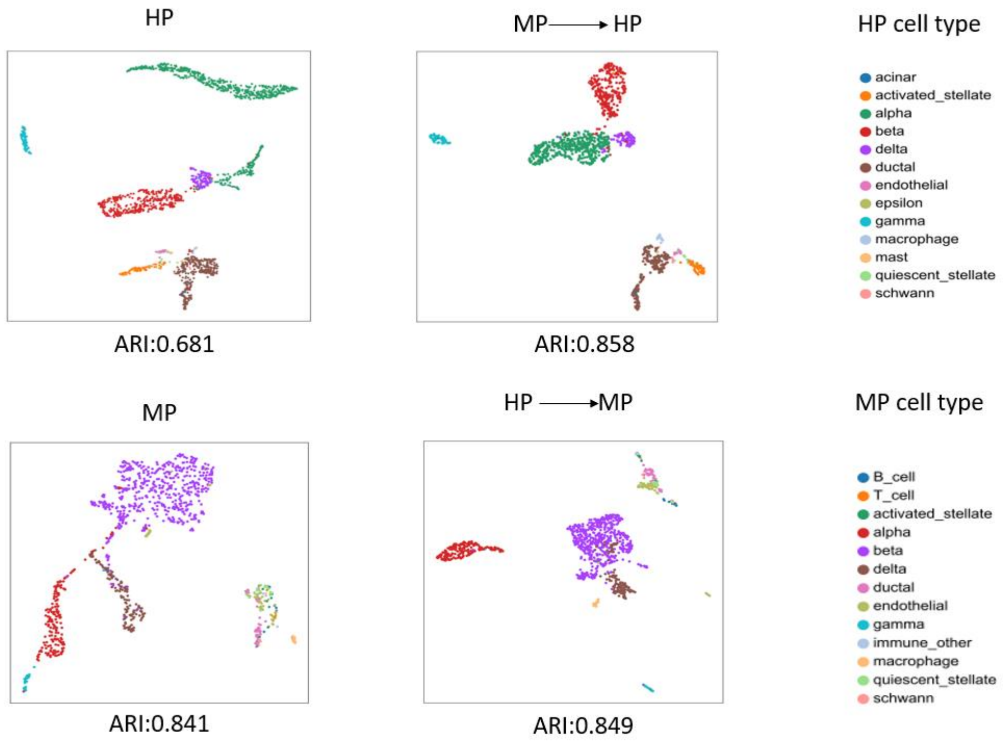

Figure 10
