# Supplementary material for: Imputation method for single-cell RNA-seq data using neural topic model
Source: Gigascience. 2023 Nov 24;12:giad098. doi: 10.1093/gigascience/giad098 (PMC10673642; doi:10.1093/gigascience/giad098)
Supplement: giad098_GIGA-D-23-00090_Revision_1 [file giad098_giga-d-23-00090_revision_1.pdf]

GigaScience

Imputation Methods for Single-Cell RNA-seq Data Using Neural Topic Models

--Manuscript Draft--

**Manuscript Number:**	GIGA-D-23-00090R1
**Full Title:**	Imputation Methods for Single-Cell RNA-seq Data Using Neural Topic Models
**Article Type:**	Technical Note
**Funding Information:**	
**Abstract:**	Single-cell RNA sequencing (scRNA-seq) technology studies transcriptome and cell-to-cell differences from higher single-cell resolution and different perspectives. Despite the advantage of high capture efficiency, downstream functional analysis of scRNA-seq data is made difficult by the excess of zero values, i.e., the dropout phenomenon. To effectively address this problem, we introduced scNTImpute, an imputation framework based on a neural topic model. A neural network encoder is used to extract underlying topic features of single-cell transcriptome data to infer high-quality cell similarity. At the same time, we determine which transcriptome data are affected by the dropout phenomenon according to the learning of the mixture model by the neural network. On the basis of stable cell similarity, the same gene information in other similar cells is borrowed to impute only the missing expression values. By evaluating the performance of real data, scNTImpute can accurately and efficiently identify the dropout values and imputes them accurately. In the meantime, the clustering of cell subsets is improved and the original biological information in cell clustering is solved which is covered by technical noise. The source code for the scNTImpute module is available as open source at https://github.com/qiyueyang-7/scNTImpute.git.
**Corresponding Author:**	Lin Liu<br>Yunnan Normal University<br>Kunming, CHINA
**Corresponding Author Secondary Information:**	
**Corresponding Author's Institution:**	Yunnan Normal University
**Corresponding Author's Secondary Institution:**	
**First Author:**	Yueyang Qi
**First Author Secondary Information:**	
**Order of Authors:**	Yueyang Qi
	Shuangkai Han
	Ling Tang
	Lin Liu
**Order of Authors Secondary Information:**	
**Response to Reviewers:**	Dear Editor:<br>On behalf of my co-authors, we thank you and the reviewer very much for giving us an opportunity to revise our manuscript entitled “Imputation Methods for Single-Cell RNA-seq Data Using Neural Topic Models” (Original paper ID: GigaScience - GIGA-D-23-00090). We also thank the reviewers of your journal for their comments on our manuscript. Those comments are all valuable and very helpful for revising and improving our paper, as well as the important guiding significance to our researches. We have studied comments carefully and tried our best to make corrections and add contents according to the comments. Enclosed please find the revised manuscript, together with a detailed list of the responses (in blue) regarding the specific points raised by the two reviewers and the editor as below.<br>We believe that both the manuscript’s quality and clarity have improved due to these revisions, and we hope that the paper is now in a form suitable for publication in GigaScience as a research article.

Powered by Editorial Manager® and ProduXion Manager® from Aries Systems Corporation

Sincerely,
Yueyang Qi

In addition, please register scNTImpute in the bio.tools and SciCrunch.org databases to receive RRID (Research Resource Identification Initiative ID) and biotoolsID identifiers, and include these in your manuscript. Computational workflows should be registered in workflowhub.eu and the DOIs cited in the relevant places in the manuscript. These will facilitate tracking, reproducibility and re-use of your tool.
Reply: Following the editor's suggestion, we have successfully registered scNTImpute on bio.tools and SciCrunch.org databases. Furthermore, we have updated the "Availability of Source Code and Requirements" section of the manuscript to include our RRID and biotoolsID identifiers. We attempted to register scNTImpute on workflowhub.eu to obtain DOIs, but it requires creating or joining a team to register content. Unfortunately, our application to create a team was unsuccessful, which means we are currently unable to obtain DOIs.

Reviewer 1:
General Comments:
The authors have addressed an important topic in scRNA-seq data imputation due to dropout events. The proposed methodology looks suitable for GigaScience in 2023. The experimental results have been properly obtained. The utility of the proposed method has also been demonstrated with software availability. I have few comments:

1. There are already many data imputation methods for scRNA-seq in 2023. The authors may wish to discuss or compare scNTImpute with others.
Reply: We are sorry for not being able to adequately present the results of the model's comparative experiments. Following the reviewer’s suggestion, we have added several published imputation methods from 2022 to 2023 to compare with our model, including, scGGAN with AE-TPGG published in 2023, and scISR published in 2022. We performed comparison experiments with scGGAN on the human brain (GSE67835) dataset (specific results in Figure 2 and Table 1); with AE-TPGG on the Deng dataset (GSE45719) (on pages 13 and 14) (specific results in Figure 6 and Table 3); with scISR, scScope (2019), and scGNN (2021) on the Human Pancreatic islet and Romanov (GSE74672) datasets (on page 15) (results are shown in Figure 8, Figure 9).

2. The actual molecular insights can also be enriched to demonstrate the practical values of scNTImpute.
Reply: We believe that the core strength of the scNTImpute model lies in its learnt topics, biological significance of the topic matter from a molecular perspective, as demonstrated by experiments with real scRNA-seq data. In the section "Path enrichment analysis and statistical significance test of scNTImpute topics" (Chapter 3, Section 5), we added new content that explores the biological relevance between the topics learned in scNTImpute and known gene pathways in humans. In this part of the research, we detected many enriched pathways based on topics learned by scNTImpute from the Human Pancreatic islet dataset, which contains many pathways related to pancreatic function, including the insulin receptor recycling, and cardiac myocyte insulin receptor signaling pathway, etc (Figure 17, Figure 18). Additionally, the topics showed varying levels of significant enrichment in these pathways.

3. Computational complexity analysis or running time should be added for clear demonstration.
Reply: Following the reviewer’s suggestion, we have added a section on “Scalability and Efficiency” in which we conducted a time complexity analysis of scNTImpute (on pages 23 and 24). We explore the relationship between runtime and the number of genes. (Figure 20).

4. Source code links can also be added at the end of abstract for easy reading.
Reply: Thanks to the reviewers for their careful reminders. Following the reviewer’s suggestion, we have added a link to our source code at the end of our abstract for the convenience of our readers.

5. Statistical significance testing should be properly performed.

Powered by Editorial Manager® and ProduXion Manager® from Aries Systems Corporation

	Reply: We are so sorry for neglecting proper statistical significance testing. So, we also added the differential analysis of topics in the "Path enrichment analysis and statistical significance test of scNTImpute topics" section, using the topics learnt from the Human Pancreatic islet dataset (on pages 22 and 23). Based on the differences in the enrichment levels of topics within pathway, we found significant variations between topics (Figure 19).<br><br>Reviewer 2:<br>General Comments:<br>This paper proposed an imputation framework based on a neural network topic model, scNTImpute, for scRNA-seq data. It extracts underlying topic features of scRNA-seq data to help with the inference of cell similarity. Several experiments were conducted to testify the performance of the proposed method. The method is reasonable and the paper is generally well-written. My specific comments are shown as below:<br><br>1. I suggest to show the loss function of the model and explain how the loss function make the model achieve the goal of extracting topic features of data.<br>Reply: We are so sorry for not providing an explanation of the loss function. In the "Topic generation process" section of the original manuscript, the process of acquiring our topics is mainly introduced. So, we have added the model's loss function here and explained how the topic features of single-cell sequencing data are extracted based on this loss function (on pages 26, 27).<br><br>2. There is a presumption for transfer learning that the data distribution of source domain should be similar to that of target domain. Therefore, the authors should demonstrate the similarity of the data sets used in the transfer learning section.<br>Reply: Following the reviewer's suggestion, we have newly added a demonstration of data similarity in the "Transfer learning across single-cell datasets" section. We demonstrate this from two main perspectives. Firstly, mean, variance and standard deviation are commonly used statistical metrics to analyse the features and similarity of the datasets. By examining at these three metrics (Table 6, Figure 14), we find that the degree of dispersion, as well as the central tendency of the two datasets (Human Pancreatic islet and Mouse Pancreatic islet) are very close to each other (on page 19). Secondly, the Probability Distribution Function (PDF) is also helpful for understanding and analyzing the similarity between datasets (on page 20). By visualizing the PDFs, it is evident that the distributions of the two datasets are highly similar (Figure 15).<br><br>3. The overall performance of the proposed method has been demonstrated in the paper. It would be helpful if one or two examples, which show the contribution of the extracted topic features, could be included.<br>Reply: Following the reviewer's suggestion, we have included a new section titled "Path enrichment analysis and statistical significance test of scNTImpute topics" (on pages 22, 23). In this section, we first investigated whether the topics learnt by scNTImpute are biologically relevant to known genetic pathways in humans. Using the Human Pancreatic Islet dataset, we identify many enriched pathways associated with pancreatic function. Many of these pathways are relevant to pancreatic function (Figure 17, Figure 18). In addition, the difference in the degree of enrichment of topics in the pathway also indicates the significant differences between the topics found in our model. (Figure 19). Of course, the ultimate goal of the scNTImpute model is to use the topics to compute cellular similarities for accurate imputation, which is also described in the "Imputation" section of the paper.
**Additional Information:**	
**Question**	**Response**
Are you submitting this manuscript to a special series or article collection?	No
**Experimental design and statistics**<br><br>Full details of the experimental design and statistical methods used should be given	Yes

Powered by Editorial Manager® and ProduXion Manager® from Aries Systems Corporation

in the Methods section, as detailed in our Minimum Standards Reporting Checklist. Information essential to interpreting the data presented should be made available in the figure legends.<br><br>Have you included all the information requested in your manuscript?	
**Resources**<br><br>A description of all resources used, including antibodies, cell lines, animals and software tools, with enough information to allow them to be uniquely identified, should be included in the Methods section. Authors are strongly encouraged to cite Research Resource Identifiers (RRIDs) for antibodies, model organisms and tools, where possible.<br><br>Have you included the information requested as detailed in our Minimum Standards Reporting Checklist?	Yes
**Availability of data and materials**<br><br>All datasets and code on which the conclusions of the paper rely must be either included in your submission or deposited in publicly available repositories (where available and ethically appropriate), referencing such data using a unique identifier in the references and in the "Availability of Data and Materials" section of your manuscript.<br><br>Have you have met the above requirement as detailed in our Minimum Standards Reporting Checklist?	Yes

Manuscript

Click here to access/download;Manuscript;(Revised)original manuscript Oct 18 clean.docx

Imputation Methods for Single-Cell RNA-seq Data Using Neural Topic Models

Yueyang Qi[1], Shuangkai Han[2], Ling Tang[3], Lin Liu[*]
[1]Yunnan Normal University, Kunming, 650500, China, E-mail: qyy18848869532@163.com.
[2]Yunnan Normal University, Kunming, 650500, China , E-mail: han_skai@163.com.
[3]Yunnan Normal University, Kunming, 650500, China , E-mail: maitanweng2@163.com.
[*]Correspondence address. Lin Liu is with the Yunnan Normal University, Kunming 650500. E-mail: liulinrachel@163.com.

Yueyang Qi [0009-0006-3210-6055];
Shuangkai Han [0009-0002-9337-5890];
Lin Liu [0000-0002-8773-6162].

Abstract

Single-cell RNA sequencing (scRNA-seq) technology studies transcriptome and cell-to-cell differences from higher single-cell resolution and different perspectives. Despite the advantage of high capture efficiency, downstream functional analysis of scRNA-seq data is made difficult by the excess of zero values, i.e., the dropout phenomenon. To effectively address this problem, we introduced scNTImpute, an imputation framework based on a neural topic model. A neural network encoder is used to extract underlying topic features of single-cell transcriptome data to infer high-quality cell similarity. At the same time, we determine which transcriptome data are affected by the dropout phenomenon according to the learning of the mixture

model by the neural network. On the basis of stable cell similarity, the same gene information in other similar cells is borrowed to impute only the missing expression values. By evaluating the performance of real data, scNTImpute can accurately and efficiently identify the dropout values and imputes them accurately. In the meantime, the clustering of cell subsets is improved and the original biological information in cell clustering is solved which is covered by technical noise. The source code for the scNTImpute module is available as open source at https://github.com/qiyueyang-7/scNTImpute.git.

Introduction

Bulk-cell RNA-seq techniques have been widely used for transcriptome analysis to study transcriptional structure, splicing patterns, and expression levels of genes and transcriptomes [1]. To address biological issues such as cell heterogeneity and gene expression randomness, it is particularly important to interpret cell-specific transcriptome landscapes [2]. Although the bulk-cell RNA-seq technique is popular, it measures the average expression level of genes in batch cells, and the expression of variable genes will be pulled to average. Therefore, it is not possible to study cell specificity based on transcriptomics. Fortunately, by studying gene expression status in single cells, scRNA-seq technology overcomes the shortcomings of

traditional batch cell sequencing technology and is becoming a powerful tool to capture the inter-cell variability of the transcriptome. It has dramatically changed the study of transcriptomics, helping us to decode life from a higher resolution and spatiotemporal structure, accurately reflecting the heterogeneity between cells. The study of scRNA-seq data has become a hot subject today.

Currently, we use multiple scRNA-seq platforms, the two most popular being Fluidigm and Drop-Seq. The Drop-Seq processes thousands of cells in a single run, which not just saves time and cost, but also is simple to operate. Fluidigm, while it usually processes fewer cells, has higher coverage rates. So， an increasing number of studies are using these techniques to discover new cell types [3,4], new markers for specific cell types [3,5,6], and cell heterogeneity [6,7,8,9,10,11].

However, scRNA-seq technology has its corresponding drawbacks. ScRNA-seq data have a relatively higher noise level than batch cell RNA-seq data, resulting in a major problem that is the sparsity of the gene expression matrix, i.e., the data often exhibits a large number of zero values [12]. Most of these zeros are artificially caused by defects in sequencing techniques, including, but not limited to, inadequate gene expression, low capture rates and sequencing depth, or other technical factors [13,14]. As a result, the observed zero value does not reflect the underlying true expression level [15,16]. This

gene expression bias may be further increased during subsequent amplification steps. Thus, dropout events can significantly affect downstream bioinformatics analysis. At present, researchers have proposed a variety of imputation models through different principles and methods [17,18]. These research results have a great guiding role in scRNA-seq data integration, enrichment analysis, etc. According to the design characteristics of the imputation algorithm, the data feature learning and processing methods, we roughly divide the RNA-seq data imputation methods into two categories: deep learning-based imputation method and non-deep learning imputation method [19].

In the traditional non-deep learning imputation algorithm, because of its simple idea, it is able to usually fit the corresponding statistical probability model or use the expression matrix for smoothing and diffusion. So, there are certain advantages in some specific types of samples. Florian Wagner et al. used the KNN-smoothing method by finding k-nearest neighbors between cells and aggregating gene-specific UMI counts to impute the gene expression matrix. In finding the number of nearest neighbors k, instead of using a way to fit a certain model, the data's imputation is achieved stepwise by constructing a partially smoothed profile with a variance-stabilizing transformation [20]. Li et al. introduced a statistical method, scImpute [21], which uses a mixture model to learn the loss probability

of each gene in each cell. By setting a loss probability threshold, the input data is divided into two parts: the set of genes severely affected by "dropout" A_j and the set of unaffected genes B_j. Eventually, the information on similar cells is learned from B_j for imputation. ScImpute automatically identifies possible dropout values and performs imputation only on these values without introducing new biases to the rest of the data. Nancy R. Zhang et al. proposed SVAER algorithm, which is a method that uses information across genes and cells to impute zero values so as to optimize the expression of all genes. By looking for potential relationships between genes, the true expression level of each gene in each cell can be restored, eliminating technical differences. Nevertheless, SVAER alters all gene expression levels, including those not affected by dropout events, which could introduce new biases into the data and potentially eliminate biologically significant variation [22]. For scRNA-seq data that are large, often high-dimensional, sparse, and complex, analysis using traditional computational methods becomes difficult and infeasible [23,24].

As deep neural network algorithms have gained great application in biomedical fields in recent years, they mine complex relationships within single-cell data through a series of basic hierarchical operations. The typical deep learning algorithms applied to scRNA-seq data are Autoencoders (AE), Variational Auto-Encoders (VAE),

Generative Adversarial Networks (GANs), and other models. Fabian J.Theis et al. proposed the Deep Count Autoencoder Network (DCA) model by improving the conventional autoencoder. The reconstruction error is defined as the probability of the noise model distribution rather than the reconstruction of the input data itself. Gene specific distribution parameters are learned by minimizing reconstruction errors in an unsupervised manner. The noise model is eventually applied to sparse count data, giving it a loss function specifically for scRNA-seq data. Meanwhile, its deep learning framework is capable of capturing the complexity and nonlinearity of scRNA-seq data and is highly scalable [25]. Lana X. Garmire et al. proposed a deep neural network based-imputation algorithm (DeepImputes) by constructing multiple sub neural-networks, which imputes genes in a divide-and-conquer manner, not only achieving the highest overall accuracy but also providing faster computing time and requiring less memory [26]. Xu et al. proposed a scRNA-seq data imputation method (scIGANs) founded on generative adversarial network. The method uses networks to generate cells rather than cells observed in the original matrix to balance the performance between dominant and rare cell populations. Enabling it to learn nonlinear gene-to-gene dependencies from complex samples of multicellular types and train generative models to generate realistic expression profiles of defined cell types. After training, K-Nearest

Neighbors (KNN) is used to impute the same type of cells, thereby eliminating technical variations without damaging inter-cell biological variability. This method is robust to small data with low expression or inter cell differences [27,28,29]

Because most downstream analyses of scRNA-seq, such as differential gene expression analysis, cell-type specific gene identification, and new cell type definition, rely on the accuracy of gene expression measurements. Therefore, it is particularly important to correct the expression of "false zero values" caused by dropout events in scRNA-seq data through accurate and robust imputation methods. [21]. These imputation methods identify the dropout values in scRNA-seq data from different perspectives and impute them. However, for non-deep learning, it is impossible to effectively learn the feature relationship of some complex nonlinear data, and it does not have good flexibility and expansibility. The architecture of deep learning itself is a 'black box', with many learning layers and thousands of nodes, making the underlying features learned and the full rich potential of the single-cell dataset unleashed uninterpretable [30].

Despite the study of RNA-Seq data is an active area of research, accurate recovery of single-cell gene expression data remains a great challenge. Inspired by neural topic, we design an accurate and stable imputation method, called scNTImpute, that can more precisely impute

gene expression affected by dropout. Specifically, scNTImpute performs deep feature extraction and the construction of networks of encoders through the coding learning mechanism of transferable neural networks. Learning network parameters and highly interpretable mixtures of cell-topic from scRNA-seq data. Topic features can be used to learn the similarity of cells, and researchers are capable of performing topic pathway enrichment analysis on them at a later stage. This is used to explore whether they have relevance to currently known gene pathways, as well to uncover topics that may be condition-specific or cell type-specific to improve the interpretability of deep feature from a biological perspective. Concurrently, we will get underlying connections such as cell-to-cell, cell-to-gene, or gene-to-gene in single-cell data. The flexibility of the neural topic model makes it excellent for processing scRNA-seq data. Besides, scNTImport uses neural networks to learn the mixture model parameters of gene expression distribution, solving the dropout probability of each gene in each cell. This allows us to more directly understand the true state of the expression data of the scRNA-seq transcriptome and distinguish which gene transcripts are affected by dropout. Using information about the same gene in other similar cells to impute the dropout value in a cell through underlying cell-gene connections. Prior to this, make sure that the borrowed information is selected for genes that are as free

as possible from dropout events.

Results

scNTImpute model overview

We propose a new scRNA-seq data imputation method on account of a neural topic model. Adapted from the single-cell embedded topic model (scETM), which inherits the advantages of topic modelling and is very effective in dealing with heavy-tailed and large distributions of word frequencies [31,32]. For the analysis of the scRNA-seq data study, we pass the sampled cell and transcriptome expressions separately as vectors of normalized counts to two fully connected neural networks (i.e., two-layer fully connected encoders). Firstly, using a fully connected neural network encoder, we infer the topic mixing ratio of cells, namely, the cell-topic mixture (Figure 1a). Secondly, we use the second neural network to infer the mixed distribution parameters of the transcriptome and obtain probability estimates of whether the gene expression value in each cell is dropout value by using the mixed distribution model. Finally, the cell-topic mixture infers similar cells of the cell in which the dropout gene is located and use the same genetic information from similar cells for the imputation of dropout values (Figure 1b).

Figure 1: Overview of the scNTImpute workflow. **a** scNTImpute uses a

neural-topic network architecture to model the single-cell transcriptome. Normalized counts of the gene expression data matrix and its transpose matrix for each single-cell dataset are used as input to the encoder. The encoder network generates random samples of potential cell-topic mixtures (θ_d, cells d=1, ..., N) that can be used to compute inter-cell similarity. Neural networks learn the parameters of a mixture model of gene expression data and can be used to identify dropout values. **b**, Imputation works using similar cell information. A cell similarity matrix is generated by calculating the intercellular similarity from the resulting mixture of cell-topic. In view of the learned parameters of the mixture model, the dropout value is identified and imputed with the information of similar cells (cell j) of the cell where the dropout value is located (cell d). **c**, Transfer learning workflow. The scNTImpute model trained on the reference scRNA-seq dataset can infer the mixture of cell-topic θ and the mixture model distributions from the unseen scRNA-seq dataset and perform accurate imputation on the unseen dataset. The scRNA-seq dataset is visualized by UMAP and evaluates by standard unsupervised clustering metrics using real cell types.

scNTImpute can efficiently impute scRNA-seq data

Recovery of biologically significant gene expression from dropout

events is the primary goal of scRNA-seq imputation, which can further reduce the impact on downstream analysis. In order to accurately evaluate the imputation performance of different models, we use published real data sets for experiments (including human brain single-cell datasets [33], Chung [34]). scNTImpute stably provides competitive results. To intuitively see the imputation performance between models, four indexes are adopted as the benchmark (i.e., ARI-Adjusted Rand Index, RI-Rand Index, NMI-Normalized Mutual Information, and MI-Mutual Information). To be specific, we used scNTImpute and several other advanced imputation methods to evaluate real human brain scRNA-seq datasets (i.e., scGGAN (scGGAN-fc, scGGAN-ng) [35], SCRABBLE [36], DCA [25], MAGIC [37], DeepImpute [26], scIGANs(w/) [27], AutoImpute [38], DrImpute [39], ENHANCE [40], SAVER [22], scGAIN [41], scImpute [21], VIPER [42], scIGANs(w/o) [27]). By visualizing the evaluation results (Figure 2), we can intuitively see that the values of the four imputation evaluation indicators of scNTImpute are relatively high (specific imputation comparison results are shown in Table 1). After imputation, we used Leiden [43] clustering and UMAP visualization for the complete scNA-Seq data (Figure 3). The results show that scNTImpute accurately and effectively recovers biologically significant gene expression from single-cell datasets.

	NMI	ARI	MI	RI

scNTImpute	0.6873	0.6857	1.3015	0.906
scGGAN	0.395	0.301	NA	NA
scGGAN-fc	0.386	0.286	NA	NA
scGGAN-ng	0.389	0.292	NA	NA
SCRABBLE	0.126	0.083	0.225	0.48
DCA	0.496	0.328	0.886	0.78
MAGIC	0.615	0.39	1.169	0.812
DeepImpute	0.568	0.364	1.057	0.802
scIGANs(w/)	0.54	0.364	0.999	0.802
DrImpute	0.642	0.471	1.189	0.876
SAVER	0.602	0.575	1.076	0.833
scGAIN	0.138	0.092	0.246	0.862
scImpute	0.672	0.545	1.223	0.856
VIPER	0.544	0.306	0.972	0.626
scIGANs(w/o)	0.349	0.243	0.631	0.631

Table 1

We perform imputation experiments on another published real dataset, Chung [34]. The above imputation indexes are not the only criteria for evaluating the imputation of RNA-SEQ data. Different from the above, the other two imputation indexes are used for evaluation (Cosine Similarity (CS), Fowlkes-Mallows Score (FMS)). Similarly, we compare it with several other existing excellent imputation models. The

evaluation results are visualized (Figure 4), from which we can see that our model performs the best in both cosine similarity and fowlkes-mallows scores. Especially in FMS, a large gap is drawn with other imputation methods. (The specific imputation comparison results are shown in Table 2, and Figure 5 shows the clustering effect on the complete Chung data set).

	CS	FMS
scNTImpute	0.5394	0.8264
Magic	0.4890	0.5493
DCA	0.3280	0.4080
DeepImpute	0.2668	0.4392
SAUCIE	0.4762	0.5372
scIGANs	0.5048	0.5961
scImpute	0.4413	0.5531
SCVI	0.2071	0.2833

Table 2

To more effectively validate the robustness and stability of scNTImpute, and highlight the strengths of our model. We apply scNTImpute to more diverse real scRNA-seq datasets. In addition to the existing comparison methods mentioned above, we include several more advanced imputation methods for comparison (i.e., AE-TPGG [44], scGNN [45], scISR [46], scScope [47]). Besides, since the cell-cell distance matrix in MAGIC

is based on Euclidean distances, the added MAGIC-C method is based on counting the data to understand how the form of the data affects the imputation. For the convenience of comparison and differentiation, the original MAGIC method based on normalized data is referred to as MAGIC-N [44]. Firstly, we applied the model to a temporal scRNA-seq dataset, mouse preimplantation embryonic development data (Deng [48]). The Deng dataset includes single cells from 10 early mouse developmental stages, ranging from zygote, 2 - /4 - /8 - /16-cell stages to blastocysts [39]. We compared scNTImpute with a new imputation method called AE-TPGG on this dataset, using the aforementioned evaluation metrics (ARI, NMI). We visualized the imputation results (Figure 6), from which we can observed that scNTImpute achieved the highest scores in both metrics, followed by our newly added AE-TPGG imputation method (Figure 7 shows the clustering of the Deng dataset after imputation using scNTImpute). Specific imputation result data can be found in Table 3.

	ARI	NMI
scNTImpute	0.552	0.750
AE-TPGG	0.448	0.643
DCA	0.291	0.513
SAVER	0.424	0.623
scImpute	0.309	0.528
MAGIC-C	0.247	0.491

MAGIC-N	0.366	0.619

Table 3

On two additional real datasets (Human Pancreatic islet (HP) [49], Romanov [50]), we compared three new imputation methods. scScope is a scalable deep-learning-based approach. The scGNN employs a graph neural network, which provides a hypothesis-free deep learning framework for scRNA-Seq analysis. In contrast, scISR is a single-cell imputation method that utilizes subspace regression. We applied these three new methods alongside our model to these two real datasets. The evaluation was performed using the same metric, ARI. To visualize the results more intuitively, we plotted the imputation results of these methods (Figures 8 and 9 represent the imputation results for the Human Pancreatic islet dataset and Romanov dataset respectively) . From the figures, it is evident that scNTImpute achieves ARI scores close to 0.7 on both datasets, outperforming the other imputation methods (achieving the highest ARI). This further confirms scNTImpute effectiveness in recovering true biological information from sparse single-cell data.

scNTImpute improves the clustering of cell subpopulations

To test the ability of scNTImpute to improve cell type or cell subgroup clustering, we applied scNTImpute to real scRNA-seq datasets, i.e.,

also on the Chung [34] dataset. In addition to reusing the above ARI and NMI evaluation indexes, we also adopt another commonly used clustering index AMI (Adjusted Mutual Information). We impute scRNA-seq data with scNTImpute and other different imputation models, and compare cell clustering with complete imputation data. Through the comparison of evaluation data (refer to Table 4 for specific data, Figure 10: visualization of comparative data), our imputation method is the highest in ARI index and has relatively significant and stable performance in AMI and NMI clustering index (Figure 11 shows the clustering effect after imputation).

	ARI	AMI	NMI
scNTImpute	0.6403	0.5071	0.5093
Magic	0.3851	0.6195	0.6304
DCA	0.2362	0.4150	0.4327
DeepImpute	0.2625	0.3071	0.3225
SAUCIE	0.3706	0.6053	0.6165
scIGANs	0.4511	0.6102	0.6199
scImpute	0.3967	0.4986	0.5069
SCVI	0.1078	0.2463	0.2679

Table 4

Moreover, we evaluated the clustering effect of scRNA-seq data after imputation on another Hrvatin real data set [3]. We use the cell type stated in the original publication as the basic fact and ARI as a performance indicator. Unlike the previous comparison, here we combine the scNTImpute with other developed imputation models and clustering methods to evaluate; that is, before using the clustering algorithm, use other imputation models to process and compare the results with our model. Several excellent clustering algorithms, such as pcaReduce [51], SC3 [52], and t-SNE [53] followed by k-means (t-SNE/kms), are used to cluster scRNA-seq data. These methods do not explicitly address the dropout events in scRNA-seq data. Therefore, in model comparison, there are two assumptions: (1) Preprocessing of dropout event RNA-Seq data by other imputation algorithms will improve the accuracy of these clustering methods, and (2) Comparison between scNTImpute and existing splendid imputation algorithms. Existing scRNA-seq imputation tools such as DrImpute [39], CIDR [54], scImpute [21], and MAGIC [37]. ScNTImpute performs better in handling dropout events to improve clustering performance (Figure 12 shows the visualization of evaluation data, see Table 5 for specific data). We can clearly see the experimental comparison of five imputation methods and individual imputation methods combined with clustering algorithms. We found that the effect of scNTImpute was significantly better than the clustering

enhancement performance of CIDR, followed by the SC3+ DrImpute (Figure 13. Clustering after Hrvatin [3] imputation using scNTImpute).

	ARI
scNTImpute	0.88
pcaR_M+ DrImpute	0.64
pcaR_M	0.53
pcaR_S+ DrImpute	0.58
pcaR_S	0.54
SC3+ DrImpute	0.76
SC3	0.77
t-SNE/kms+ DrImpute	0.60
t-SNE/kms	0.52
CIDR	0.16
scImpute	0.56
MAGIC	0.45

Table 5

Transfer learning across single-cell datasets

A prominent feature of scNTImpute is its parameters, so the knowledge of modeling scRNA-seq data can be transferred across datasets. As part of scNTImpute, the model trained on the reference scRNA-seq dataset can be applied to infer the cell-topic mixture and the parameters of

the mixture model for the target scRNA-seq dataset, without ensuring that the two datasets share the same cell type. To illustrate, we employ two real RNA-Seq data sets: Human Pancreatic islet data sets and Mouse Pancreatic islet data sets (MP) [32] were used to conduct cross-species transfer learning of scNTImpute models. Both datasets were obtained using the inDrop method (a droplet-based single cell RNA-Seq sequencing technique). The assumption of transfer learning is that the distribution of data in the source domain should be similar to the distribution of data in the target domain. Therefore, we primarily demonstrate the similarity of the datasets used in the transfer learning section from two perspectives. Firstly, mean, variance, and standard deviation are commonly used statistical measures to analyse the characteristics and similarities of datasets. If their mean is close and their variance and standard deviation are similar, then their similarity is higher. Conversely, if these measures differ significantly, their similarity is lower. By analyzing the calculations, the results of the evaluation metrics for the two datasets are shown in Table 6. We also visualize the data from Table 6 (Figure 14), which provides a more intuitive way of observation. We find that their values for all three statistical indicators are very close. Especially in terms of standard deviation, the two datasets have almost the same level of dispersion. Additionally, by comparing the mean, we can see

that the central tendencies of the two datasets are also very similar.

Evaluation index	Variance	Mean	Standard Deviation
HP	55.61	0.38	7.46
MP	55.69	0.35	7.46

Table 6

Secondly, Probability Distribution Function (PDF) is a function used to describe the probability of the possible values of a random variable. It can also help us understand and analyse similarities between datasets. For discrete data such as scRNA-seq datasets, we can calculate the frequency of occurrence for each value and divide these frequencies by the total size of the dataset to obtain the probability of each value. To facilitate the intuitive analysis of the probability distribution functions of the two datasets, we visualize the two probability distributions (Figure 15). Due to the sparsity of the original scRNA-seq datasets, the frequencies of zero values are relatively high in both datasets. By visualizing the probability distributions, we can easily observe that the distributions of the two datasets are very similar.

Next we conduct research on transfer learning. Firstly, if the HP dataset is directly trained on the model, the four imputation indicators are ARI: 0.681, NMI: 0.751, RI: 0.884, and MI: 1.429. Secondly, we train a scNTImpute model on the MP dataset and use the trained model to impute and evaluate HP data. Ultimately, an exciting transfer learning effect was produced (ARI reached 0.858 in the HP dataset, refer to Table 7 for other specific results). In order to verify the stability of the model transfer learning, we conducted the transfer learning from the HP dataset to the MP dataset, and the results were also surprising. The results of direct imputation and transfer learning imputation of the HP data set were visualized by UMAP (Figure 16. Clustering of four imputation results: direct imputation on HP, scNTImpute trained on MP to impute HP, direct imputation on MP, and scNTImpute trained on HP to impute MP). After transfer, scNTImpute improved many indicators and learned cell type-specific (Table 7, Figure 16). To compare with other methods, we use scNTImpute, scVI-LD, and scVI to evaluate clustering performance in transfer learning tasks. Clustering performance is mainly measured by the Adjusted Rand index (ARI) between real cell types and Leiden [43] clusters. Overall, scNTImpute obtains the best learning results in cross-species transfer learning between HP and MP (Table 8).

	HP	MP→HP	MP	HP→MP

ARI	0.681	0.858	0.841	0.849
NMI	0.751	0.821	0.758	0.769
MI	1.429	1.345	1.163	1.232
RI	0.884	0.946	0.931	0.933
CS	0.930	0.8378	0.901	0.863
FMS	0.757	0.894	0.892	0.901

Table 7

Source dataset	MP	HP
Target dataset	HP	MP
scNTImpute	0.858	0.849
scVI-LD	0.690	0.478
scVI	0.524	0.425

Table 8

Path enrichment analysis and statistical significance test of scNTImpute topics

We next investigated separately whether the topics of scNTImpute were biologically relevant to known human genetic pathways and whether there were differences between topics. Firstly, we employed Gene Set Enrichment Analysis (GSEA) [55] for exploration. We trained a scNTImpute with 50 topics using the HP dataset. For the obtained topics,

we detected a number of significantly enriched pathways. Several of these are related to pancreatic function, including the insulin receptor recycling, cardiac myocyte insulin receptor signaling pathway, insulin signaling pathways, pancreatic cancer, etc (Figure 17, Figure 18). The set of contained genes between the black bar in the middle and the highest point (ES value) is called the leader subset, which contribute to the upregulation of the entire pathway. Furthermore, based on the differences in the enrichment levels of topics in the pathway, there are also significant differences between topics. We calculate p-value and fold change(FC), and convert p-value as negative logarithm to $-\log_{10}(\mathrm{p}-\mathrm{value})$, while fold change is logarithmically converted to $\log(FC)$ (The red line in Figure 19 is the threshold line for $\mathrm{p} < 0.01$. In general, by taking the negative logarithm of the p-values, and majority of our topics are smaller than the set threshold, which shows that there are significant differences in our topics (Figure 19).

Scalability and efficiency

To validate the scalability and efficiency of the proposed scNTImpute for transferable learning, we tested it on datasets with different number of genes and recorded the runtime. Specifically, we take the trained model directly for imputation on additional datasets. We

performed imputation on datasets containing 1k, 2k, 5k, 10k, and 15k genes, and investigated the relationship between running time and the number of genes (Figure 20). The running time of imputation exhibited a linear increase relative to the number of genes. As scNTImpute is a neural topic-based imputation method, its runtime increases with the number of genes. In practical applications, the number of genes and cells is limited. So, scNTImpute is more suitable for scRNA-seq datasets than other imputation methods.

Methods

Workflow

We adopt an imputation workflow based on a neural topic model, implemented using the PyTorch (RRID:SCR_018536) dynamic framework on the backend. Our work is divided into the following steps.

Data preprocessing

We take as input the scRNA-seq gene count expression matrix X, where the rows represent cells and the columns represent genes. Data filtering and quality control are performed as a previous step of data preprocessing, and we eventually want to get an imputation matrix with the same dimensionality as the original count matrix. To facilitate the subsequent work, we first normalize each sample (cell) and each

gene in the matrix separately to obtain two normalized matrices, Y^C (normalized by cell) and Y^G (normalized by gene). Then log10 transforms Y^G and add pseudo-count 1.01 to generate the Y matrix [56]:

$$Y_{ij} = log_{10}(Y_{ij}^G + 1.01); \ i = 1,2,\ldots,I; j = 1,2,\ldots,J$$

I denotes the number of cells and J denotes the number of genes. To avoid infinite values of the parameters in later model training, we added pseudo-counts to it. The advantage of logarithmic transformation is that it can prevent some large observations from having a significant impact, eliminate heteroscedasticity issues, and transform the values into continuity, providing greater flexibility for modeling.

Topic generation process

Inspired by scETM's research on single-cell transcriptomics [31]. We adopted a neural topic model to model the scRNA-seq data distribution [57]. We treat each cell as a document, and each scRNA-seq read (or UMI) serves as a marker in the document. The gene that generates the read count (or UMI) is thought of as a word in a vocabulary. We assume that each cell can be represented as a mixture of underlying cell types, and they are often referred to as potential topics. The original LDA model [57] a fixed set of N-independent Dirichlet distributions β is defined, distributed over a vocabulary of size M. Formally, the cell-topic mixture generation process is as follows.

Obtaining the potential topic proportion of cell C from a logical normal distribution:

$$\delta_C \sim \boldsymbol{N}(0, I), \theta_C = softmax(\delta_C) = \frac{\exp(\delta_{c,N})}{\sum_{N=1}^{N} \exp(\delta_{c,N})}$$

$$\theta_C \sim LN(0, I)$$

Where θ_C is the $1 \times N$ cell-topic mix of cell C. To simulate the sparsity of gene expression in each cell, the softmax function is used to regulate the expression of all genes. For this purpose, we obtain a mixture of all the cell topics θ [31].

To enable the extraction of latent topics from the data, scNTImpute consider the cell-specific latent topic mixture θ_C as the unique latent variable for each cell C. We denote the posterior distribution of latent variables as $p(\delta|Y^C)$ (Y^C: normalised gene expression matrix above). However, directly solving for the true posterior distribution in high-dimensional space is computationally challenging. Therefore, a variational inference method is employed to approximate the true posterior distribution by minimizing the difference between $q(\delta_c)$ and $p(\delta|Y^C)$, and variational posterior is easier to compute compared to the true posterior. Specifically, we define the following distribution: $q(\delta|y)=\prod_c q(\delta_c|y_c)$, where $q(\delta_c|y_c) = \mu_c + dia(\sigma_c)\boldsymbol{N}(0, I)$. The parameters $[\mu_c, \log\sigma_c^2]$ are estimated by a two-layer feed-forward neural network $\text{NNET}(Y_c^C; \text{W}_\theta)$. This network approximates the complete statistics of the

cellular topic mixture δ_c. To learn variational parameter W_θ mentioned above, we optimize the evidence lower bound (ELBO) for the logarithmic likelihood [31]. This involves minimizing the Kullback-Leibler (KL) divergence, which measures the difference between the approximate posterior distribution and the true posterior distribution. The goal is to reduce this divergence and bring the approximate posterior closer to the true posterior:

$$ELBO = E[\log \mathrm{p}(\mathrm{Y}|\theta)] - D_{KL}[\mathrm{q}(\theta|\mathrm{Y})||\mathrm{p}(\theta)]$$

The first term represents the likelihood function, which is measured using Negative Log-Likelihood (NLL). The second term, the reconstruction likelihood, is a regularization term involving the KL divergence between the approximate distribution ($q(\delta_c|y_c) = N(\mu_c, diag(\sigma_c))$) and the prior distribution ($\mathrm{p}(\delta_c) = N(0, I)$) , which encourages the variational posterior distribution to approximate the prior distribution. We sample a few latent variable samples from the reparameterized Gaussian distribution $q(\delta_c|y_c)$, where their mean and variance are determined by the aforementioned NNET. These samples serve as noise estimates for ELBO [31]. Ultimately, the gradients are backpropagated to optimize the weights of the encoder to achieve the goal of extracting topic features.

Study dropout values

After acquiring the transformed gene expression matrix Y, we can infer which genes in the cell are affected by the dropout event. Instead of considering all zero values as dropout values, we use a neural network to systematically determine whether zero values are dropout values. Firstly, the normal distribution describes continuous data, while the reads count (gene expression) data is discrete. Secondly, the reads count data can only take values that are non-negative integers, and for scRNA-seq data, the most commonly used normal distribution is not reasonable. Certainly, the zero-inflated negative binomial (ZINB) distribution has proven to be a good model for describing scRNA-seq data, and serves as the basis for some outstanding models. With the presence of dropout events, most genes have bimodal expression patterns in similar cells. We adapted the mixture model used in scImpute [21]. The similar mixture models have been shown to effectively capture the bimodal features of single-cell gene expression data [32, 56, 45]. Where the Gamma distribution represents the dropout phenomenon, and the Normal distribution is used for indicating actual gene expression. It is important to note that the transformed gene expression levels are no longer integers, so the widely used read counts obeying a negative binomial distribution is not an appropriate choice. For each

gene, the proportions and parameters of the two components may distinctive in different cell types. As a result, we assume that the expression level of each gene j is a random variable Y_j following a Gamma-Normal mixed distribution, with a density function of [21]:

$$f_{Y_j}(y) = \lambda_j \cdot \text{Gamma}(y; \alpha_j, \beta_j) + (1 - \lambda_j) \cdot \text{Normal}(y; \mu_j, \sigma_j) \quad (1)$$

Where λ_j is the dropout rate of genes, the α_j and β_j are the shape and rate parameters in the gamma distribution, and μ_j and σ_j are the mean and standard deviation in the normal distribution, respectively. When a sequencing experiment fails to accurately capture the transcriptional expression of genes, the gamma distribution models the observed gene expression, while the normal distribution simulates the actual gene expression level. The intuition behind this mixture model is that if a gene has high expression and low variation in multiple cells, the ″zero″ count expression is more likely to be affected by dropout events; on the other hand, if a gene has consistently low or moderate expression and high variation, then the zero counts may reflect the true biological significance.

After a given distribution of the mixture model, the log-likelihood of each gene at all cell expression levels can be calculated as $l(\lambda_j, \alpha_j, \beta_j, \mu_j, \sigma_j) = \sum_{i=1}^{n} \log f_{Y_j}(y_{ij}; \lambda_j, \alpha_j, \beta_j, \mu_j, \sigma_j)$ [56]. The parameters in the model shown in Equation (1) are calculated by a neural network and these estimates are denoted as $\widetilde{\lambda_j}, \widetilde{\alpha_j}, \widetilde{\beta_j}, \widetilde{\mu_j}, \widetilde{\sigma_j}$. We can filter the gene

expression values based on the undetected probability of the gene in the cell [56], and the dropout rate of gene j in cell i can be computed as:

$$d_{ij} = \frac{\widetilde{\lambda_j}\text{Gamma}(Y_{ij}; \widetilde{\alpha_j}, \widetilde{\beta_j})}{\widetilde{\lambda_j} \cdot \text{Gamma}(Y_{ij}; \widetilde{\alpha_j}, \widetilde{\beta_j}) + (1 - \widetilde{\lambda_j}) \cdot \text{Normal}(Y_{ij}; \widetilde{\mu_j}, \widetilde{\sigma_j})} \quad (2)$$

Because $d_{ij} \in$ （0，1）， a smaller d_{ij} indicates that the observed gene expression Y_{ij} has higher confidence. We set the threshold t by which the dropout rate $d_{ij} < t$ is considered to be an accurate measure with high confidence, and when the dropout rate $d_{ij} \geq t$, then gene expression Y_{ij} is considered a dropout value.

Imputation

To impute the dropout values accurately, we need to borrow expression data of gene j in other similar cells that are not affected by dropout to fill in. Specifically, on the basic of the above obtained cell-topic mixture θ, the essence of which is also the dimensionality reduction of scRNA-seq data while effectively reducing the impact of most dropouts in the data. We calculate the similarity matrix of cells, where each element means how similar the cell is to other cells. The degree of similarity of cell i and other cells is calculated as follows:

$$Z_{ii'} = min\sqrt{\sum_{i'=1,i=1}^{I,I} (\theta_i - \theta_{i'})^2} \quad (i', i = 1,2,3 \ldots I)$$

Where Z indicates the degree of similarity between cell i and cell i'. A larger value indicates that two cells are less likely to belong to the same type of cell and less similar, and a smaller value indicates greater similarity (excluding the degree of similarity with itself, i.e., 0 value). In compliance with the similarity between cells, we can borrow the same non-dropout gene expression data from similar cell i' of cell i for the imputation of dropout genes in cell i.

$$\check{X}_{ij} = X_{i'j}, (d_{i'j} < t)$$

Imputation evaluation

To benchmark the imputation performance, we compared several scRNA-seq data imputation tools that are identical to scNTImpute. We utilized the original data set for the evaluation experiments. After the imputation of the original data was completed, four leading evaluation indicators, ARI (Adjusted Rand Index), RI (Rand index), NMI (Normalized Mutual Information), and MI (Mutual Information), were utilized for the evaluation. Where ARI is interpreted as.

$$ARI = \frac{RI - E[RI]}{MAX(RI) - E[RI]}$$

The RI is interpreted as.

$$RI = \frac{a + b}{C_n^2}$$

Where, a indicates the correct number of markers that cells should have been of the identical type and after clustering are also in the

same type; b represents the correct number of markers that cells are not of the same type and do not cluster to the identical type after clustering. C_n^2 represents the total number of possible pairs. And $E[RI]$ is the expected RI of the random markers [45].

NMI is explained as

$$NMI(Q,R) = \frac{2MI(Q,R)}{H(Q) - H(R)}$$

MI is interpreted as.

$$MI(Q,R) = \sum_{i=1}^{|Q|}\sum_{j=1}^{|R|} P(i,j)\log(\frac{P(i,j)}{P(i)P(j)})$$

In the above equation, Q means the original category of each cell, while R indicates the category to which the cells belong after clustering. $H(Q)$ expresses the entropy of Q.

Availability of Source Code and Requirements

Project name: scNTImpute

Project homepage: https://github.com/qiyueyang-7/scNTImpute.git

Research Resource Identifier (#RRID): SCR_024395

BiotoolsID: scNTImpute

Operating system(s): Platform independent

Programming language: Python

Other requirements: conda, Python 3.7, numpy 1.21, pandas 1.3

License: MIT License

Data Availability

The scRNA-seq data used in this manuscript are all publicly available. All data are available at GEO, with human brain data [33] access number: GSE67835; Chung dataset [34] accession code: GSE75688; Hrvatin dataset [3] GEO accession code: GSE59739; Romanov dataset [50] GEO accession code: GSE74672; Deng dataset [48] GEO accession code: GSE45719; Mouse Pancreatic Islet data [32] GEO accession codes are GSE84133. Human Pancreatic Islet data [49] are available at GEO, or EMBL-EBI database with accession codes GSE81076, GSE85241, GSE86469, E-MTAB-5061 and GSE84133. All additional supporting data are available in the *GigaScience* repository, GigaDB [58].

Abbreviations

scRNA-seq: Single cell RNA sequencing; ARI: Adjusted Rand Index; RI: Rand Index; NMI: Normalized Mutual Information; MI; Mutual Information; CS: Cosine Similarity; FMS: Fowlkes-Mallows Score; AMI: Adjusted Mutual Information;

Competing Interests

The authors declare that there no competitive interests.

Authors' Contributions

Qi and Liu conceived and developed the study. Qi completed the scNTImput workflow and the writing of the main manuscript text. Han and Tang reviewed and contributed to all versions of the manuscript text. All authors read and approved the final manuscript.

Acknowledgement

This work was supported by the Applied Basic Research Project in Yunnan Province (No.202201AT070042), the National Natural Science Foundation of China (No.61862067) and the NSFC- Yunnan Union Key Grant (No.U1902201)

References

[1] Wang Z, Gerstein M, Snyder M. RNA-Seq: a revolutionary tool for transcriptomics. Nat Rev Genet . 2009;10(1):57-63. https://doi.org/10.1038/nrg2484.

[2] McDavid A, Finak G, Chattopadyay PK, et al. Data exploration, quality control and testing in single-cell qPCR-based gene expression experiments. Bioinformatics. 2013,29(4):461-467, https://doi.org/10.1093/bioinformatics/bts714.

[3] Usoskin D, Furlan A, Islam S, et al. Unbiased classification of sensory neuron types by large-scale single-cell RNA sequencing. Nat Neurosci. 2015;18(1):145-53.. https://doi.org/10.1038/nn.3881.

[4] Villani AC, Satija R, Reynolds G, et al. Single-cell RNA-seq reveals new types of human blood

dendritic cells, monocytes, and progenitors. Science. 2017;356(6335):eaah4573. doi: 10.1126/science. aah 4573.

[5] Zeisel A, Muñoz-Manchado AB, Codeluppi S, et al. Cell types in the mouse cortex and hippocampus revealed by single-cell RNA-seq. Science. 2015;347(6226):1138-42. doi: 10.1126/science.aaa1934.

[6] Jaitin DA, Kenigsberg E, Keren-Shaul H, et al. Massively parallel single-cell RNA- seq for marker-free decomposition of tissues into cell types. Science. 2014;343(6172):776-9. doi: 10.1126/science.1247651.

[7] Pollen AA, Nowakowski TJ, Shuga J, et al. Low- coverage single-cell mRNA sequencing reveals cellular heterogeneity and activated signaling pathways in developing cerebral cortex. Nat Biotechnol. 2014;32(10):1053-8. https://doi.org/10.1038/nbt.2967.

[8] Treutlein B, Brownfield DG, Wu AR, et al. Reconstructing lineage hierarchies of the distal lung epithelium using single-cell RNA-seq. Nature. 2014;509(7500):371-5 https://doi.org/10.1038/nature13173.

[9] Tirosh I, Venteicher AS, Hebert C, et al. Single-cell RNA-seq supports a developmental hierarchy in human oligodendroglioma. Nature. 2016;539(7628):309-313. https://doi.org/10.1038/nature20123.

[10] Shalek AK, Satija R, Adiconis X, et al. Single-cell transcriptomics reveals bimodality in expression and splicing in immune cells. Nature. 2013;498(7453):236-40. https://doi.org/10.1038/nature12172.

[11] Tang F, Barbacioru C, Bao S, et al. Tracing the derivation of embryonic stem cells from the inner cell mass by single-cell RNA-Seq analysis. Cell Stem Cell. 2010;6(5):468-78. doi:

10.1016/j.stem.2010.03.015.

[12] Petropoulos S, Edsgärd D, Reinius B, et al. Single-Cell RNA-Seq Reveals Lineage and X Chromosome Dynamics in Human Preimplantation Embryos. Cell. 2016;167(1):285. doi: 10.1016/j.cell.2016.08.009.

[13] Kharchenko P, Silberstein L, Scadden D. Bayesian approach to single-cell differential expressionsis. Nat Methods. 2014;11(7):740-742. https://doi.org/10.1038/nmeth.2967.

[14] Li WV, Li JJ. A statistical simulator scDesign for rational scRNA-seq experimental design, Bioinformatics. 2019;35(14):i41-i50. https://doi.org/10.1093/bioinformatics/btz321.

[15] Dijk DV, Sharma R, Nainys J, et al. Recovering Gene Interactions from Single-Cell Data Using Data Diffusion. Cell. 2018;174(3):716-729.e27. doi: 10.1016/j.cell.2018. 05.061.

[16] Vu TN, Wills QF, Kalari KR, et al. Beta-Poisson model for single-cell RNA-seq data analyses, Bioinformatics. 2016;32(14)2128–2135. https://doi.org/10.1093/bioinformatics/btw202.

[17] Backenroth D, He Z, Kiryluk K, et al. FUN-LDA: A Latent Dirichlet Allocation Model for Predicting Tissue-Specific Functional Effects of Noncoding Variation: Methods and Applications. The American Journal of Human Genetics. 2018; 102(5):920-942, https://doi.org/10.1016/j.ajhg.2018.03.026.

[18] Zhang L, Zhang S. Comparison of computational methods for imputing single-cell RNA-sequencing data. IEEE/ACM transactions on computational biology and bioinformatics. 2020. doi: 10.1109/TCBB.2018.2848633.

[19] Wu X, Zhou Y. GE-Impute: graph embedding-based imputation for single-cell RNA-seq

data. Briefings in Bioinformatics. 2022. https://doi.org/10.1093/bib/bbac313.

[20] Wagner F, Yan Y, Yanai I. K-nearest neighbor smoothing for high-through put single-cell RNA-Seq data. bioRxiv. 2017. doi: https://doi.org/10.1101/217737.

[21] Li WV, Li JJ. An accurate and robust imputation method scImpute for single-cell RNA-seq data. Nat Commun. 2018. https://doi.org/10.1038/s41467-018-03405-7.

[22] Huang M, Wang J, Torre E, et al. SAVER: gene expression recovery for single-cell RNA sequencing. Nat Methods. 2018. https://doi.org/10.1038/s41592-018-0033-z.

[23] Huang M, Ye X, Li H, et al. Missing Value Imputation With Low-Rank Matrix Completion in Single-Cell RNA-Seq Data by Considering Cell Heterogeneity. Front Genet. 2022; 13:952649. doi: 10.3389/fgene.2022.952649.

[24] Zhang L, Zhang S. Imputing single-cell RNA-seq data by considering cell heterogeneity and prior expression of dropouts. Journal of Molecular Cell Biology. 2021. https://doi.org/10.1093/jmcb/mjaa052.

[25] Eraslan G, Simon LM, Mircea M, et al. Single-cell RNA-seq denoising using a deep count autoencoder. Nat Commun. 2019. https://doi.org/10.1038/s41467-018-07931-2.

[26] Arisdakessian C, Poirion O, Yunits B, et al. DeepImpute: an accurate, fast, and scalable deep neural network method to impute single-cell RNA- seq data. Genome Biol. 2019. https://doi.org/10.1186/s13059-019-1837-6.

[27] Xu Y, Zhang Z, You L, et al. scIGANs: single-cell RNA-seq imputation using generative adversarial networks, Nucleic Acids Research. 2020. https://doi.org/10.1093/nar/gkaa506.

[28] Tian T, Min MR, Wei Z. Model-based autoencoders for imputing discrete single-cell RNA-seq data. Methods. 2021. doi: 10.1016/j.ymeth.2020.09.010.

[29] Lu F, Lin Y, Yuan C, et al. EnTSSR: A Weighted Ensemble Learning Method to Impute Single-Cell RNA Sequencing Data. IEEE/ACM Trans Comput Biol Bioinform. 2021;18(6):2781-2787. doi: 10.1109/TCBB.2021.3110850.

[30] Zhu M, Lai Y. Improvements Achieved by Multiple Imputation for Single-Cell RNA-Seq Data in Clustering Analysis and Differential Expression Analysis. J Comput Biol. 2022;29(7):634-649. doi: 10.1089/cmb.2021.0597.

[31] Zhao Y, Cai H, Zhang Z, et al. Learning interpretable cellular and gene signature embeddings from single-cell transcriptomic data. Nat Commun. 2021. https://doi.org/10.1038/s41467-021-25534-2.

[32] Baron M, Veres A, Wolock S, et al. A single-cell transcriptomic map of the human and mouse pancreas reveals inter-and intra-cell population structure. Cell Syst. 2016;26;3(4):346-360.e4. doi: 10.1016/j.cels.2016.08.011.

[33] Darmanis S, Sloan SA, Zhang Y, et al. A survey of human brain transcriptome diversity at the single cell level. Proc Natl Acad Sci. 2015;112(23):7285-90. doi: 10.1073/pnas.1507125112.

[34] Chung W, Eum HH, Lee HO, et al. Single-cell RNA-seq enables comprehensive tumour and immune cell profiling in primary breast cancer. Nat Commun. 2017. https://doi.org/10.1038/ncomms15081.

[35] Huang Z, Wang J, Lu X, et al. scGGAN: single-cell RNA-seq imputation by graph-based generative adversarial network. Briefings in Bioinformatics. 2023. https://doi.org/10.1093/bib/bbad040.

[36] Peng T, Zhu Q, Yin P, et al. SCRABBLE: single-cell RNA-seq imputation constrained by bulk

RNA-seq data. Genome Biol. 2019. https://doi.org/10.1186/s13059-019-1681-8.

[37] Dijk DV, Nainys J, Sharma R, et al. Recovering Gene Interactions from Single-Cell Data Using Data Diffusion. Cell. 2018;174(3):716-729.e27. doi: 10.1016/j.cell.2018.05.061.

[38] Talwar D, Mongia A, Sengupta D, et al. AutoImpute: Autoencoder based imputation of single-cell RNA-seq data. Sci Rep.2018. https://doi.org/10.1038/s41598-018-34688-x.

[39] Gong W, Kwak IY, Pota P, et al. DrImpute: imputing dropout events in single cell RNA sequencing data. BMC Bioinformatics. 2018. https://doi.org/10.1186/s12859-018-2226-y.

[40] Wagner F, Barkley D, Yanai I. Accurate denoising of single-cell RNA-Seq data using unbiased principal component analysis. bioRxiv. 2019. doi: https://doi.org/10.1101/655365.

[41] Gunady MK, Kancherla J, Bravo HC, et al. scGAIN: single cell RNA-seq data imputation using generative adversarial networks. bioRxiv. 2019. doi: https://doi.org/10.1101/837302.

[42] Chen M, Zhou X. VIPER: variability-preserving imputation for accurate gene expression recovery in single-cell RNA sequencing studies. Genome Biol. 2018;19(1):196. doi: 10.1186/s13059-018-1575-1.

[43] Traag VA, Waltman L, Eck NJ. From Louvain to Leiden: guaranteeing well-connected communities. Sci Rep. 2019. https://doi.org/10.1038/s41598-019-41695-z.

[44] Zhao S, Zhang L, Liu X. AE-TPGG: a novel autoencoder-based approach for single-cell RNA-seq data imputation and dimensionality reduction. Front Comput Sci. 2023;17(3):173902. doi: 10.1007/s11704-022-2011-y.

[45] Wang J, Ma A, Chang Y, et al. scGNN is a novel graph neural network framework for single-cell RNA-Seq analyses. Nat Commun. 2021. https://doi.org/10.1038/s41467-021-22197-x.

[46] Tran D, Tran B, Nguyen H, et al. A novel method for single-cell data imputation using

subspace regression. Sci Rep. 2022. https://doi.org/10.1038/s41598-022-06500-4.

[47] Deng Y, Bao F, Dai Q, et al. Scalable analysis of cell-type composition from single-cell transcriptomics using deep recurrent learning. Nat Methods. 2019. https://doi.org/10.1038/s41592-019-0353-7.

[48] Deng Q, Ramsköld D, Reinius B, et al. Single-cell RNA-seq reveals dynamic, random monoallelic gene expression in mammalian cells. Science. 2014;343(6167):193-6. doi: 10.1126/science.1245316.

[49] Stuart T, Butler A, Hoffman P, et al. Comprehensive Integration of Single-Cell Data. Cell. 2019;177(7):1888-1902.e21. doi: 10.1016/j.cell.2019.05.031.

[50] Romanov R, Zeisel A, Bakker J, et al. Molecular interrogation of hypothalamic organization reveals distinct dopamine neuronal subtypes. Nat Neurosci. 2017. https://doi.org/10.1038/nn.4462.

[51] Žurauskienė J, Yau C. pcaReduce: hierarchical clustering of single cell transcriptional profiles. BMC Bioinformatics. 2016. https://doi.org/10.1186/s12859-016-0984-y.

[52] Kiselev VY, Kirschner K, Schaub MT, et al. SC3: consensus clustering of single-cell RNA-seq data. Nat Methods. 2017. https://doi.org/10.1038/nmeth.4236.

[53] Maaten LV, Hinton GE. Visualizing Data using t-SNE. journal of Machine Learning Research. Nature Reviews Genetics. 2009; 10(57–63). https://doi.org/10.1038/nrg2484.

[54] Lin P, Troup M, Ho JW. CIDR: Ultrafast and accurate clustering through imputation for single-cell RNA-seq data. Genome Biol. 2017;18(1):59. https://doi.org/10.1186/s13059-017-1188-0.

[55] Subramanian A, Tamayo P, Mootha VK, et al. Gene set enrichment analysis: a knowledge-

based approach for interpreting genome-wide expression profiles. Proc Natl Acad Sci. 2005;102(43):15545-50. doi: 10.1073/pnas.0506580102.

[56] Li WV, Li Y. scLink: Inferring Sparse Gene Co-expression Networks from Single-cell Expression Data. Genomics, Proteomics & Bioinformatics. 2021;19(3):475-492, https://doi.org/10.1016/j.gpb.2020.11.006.

[57] Blei DM, Ng AY, Jordan MI. Latent dirichlet allocation. J. Mach. Learn. Res. 2003; 993- 1022.

[58] Qi Y, Han S, Tang L, Liu L. Supporting data for "Imputation Methods for Single-Cell RNA-seq Data Using Neural Topic Models". GigaScience Database. 2023. http://dx.doi.org/10.5524/102454.

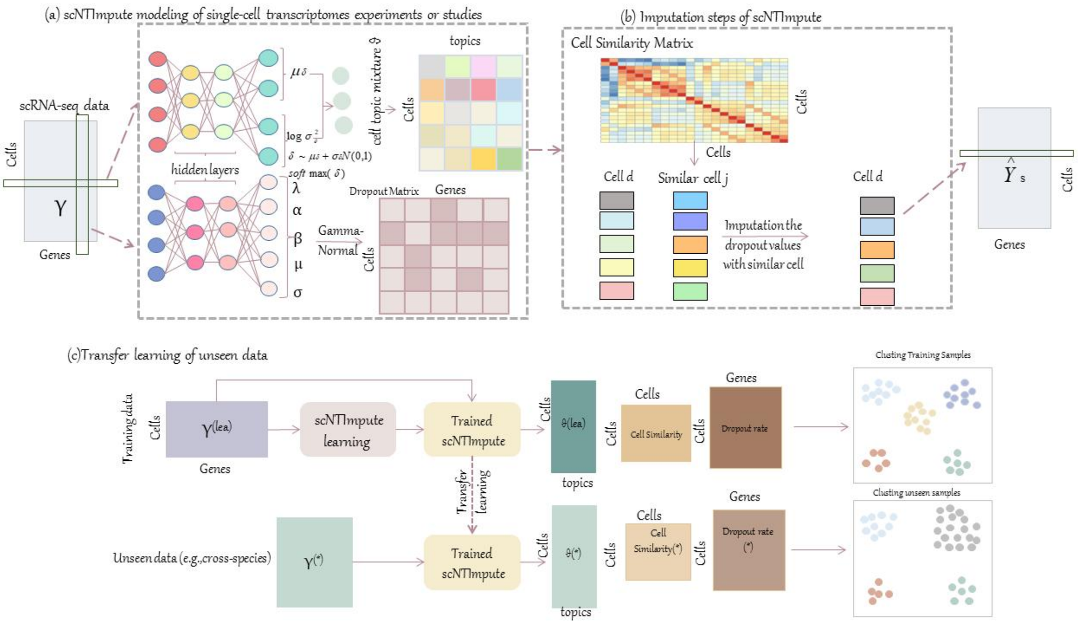

Figure 1

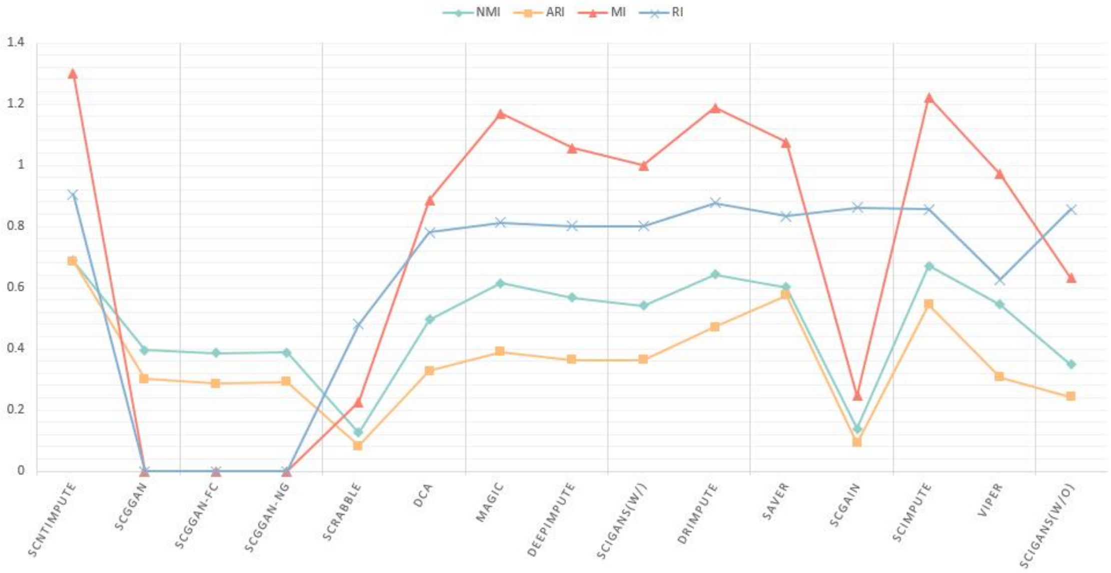

Figure 2

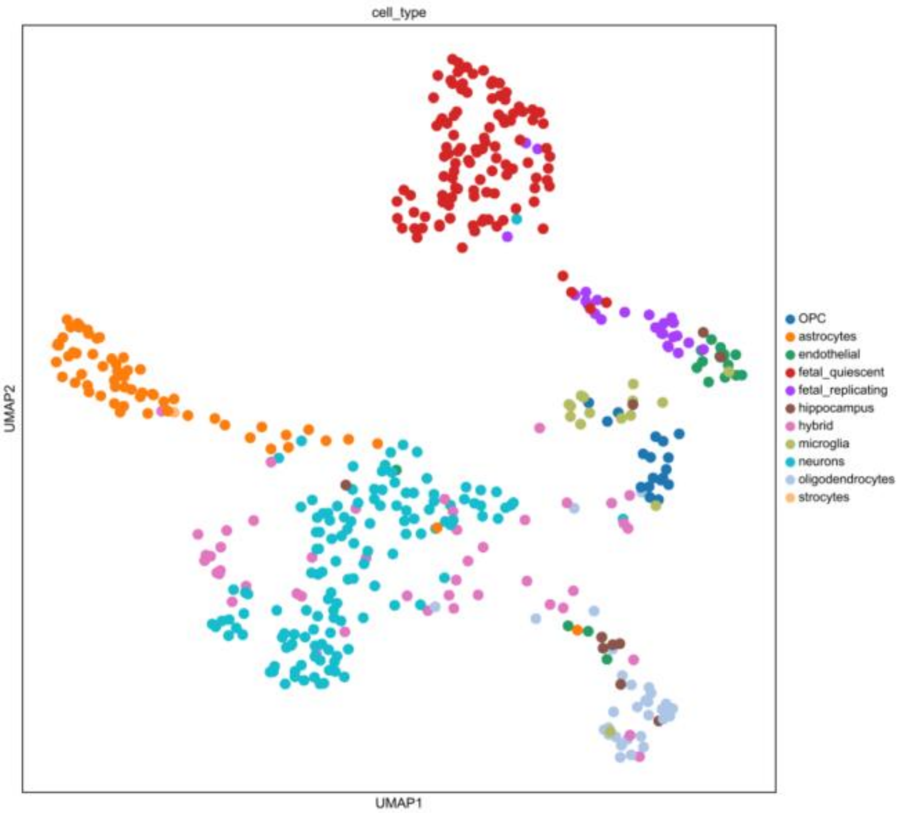

Figure 3

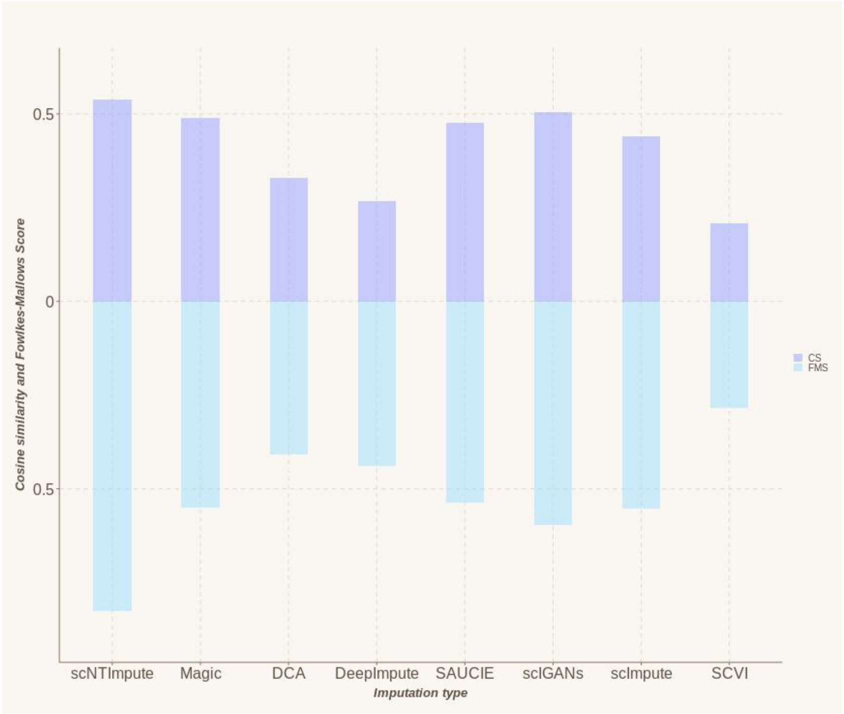

Figure 4

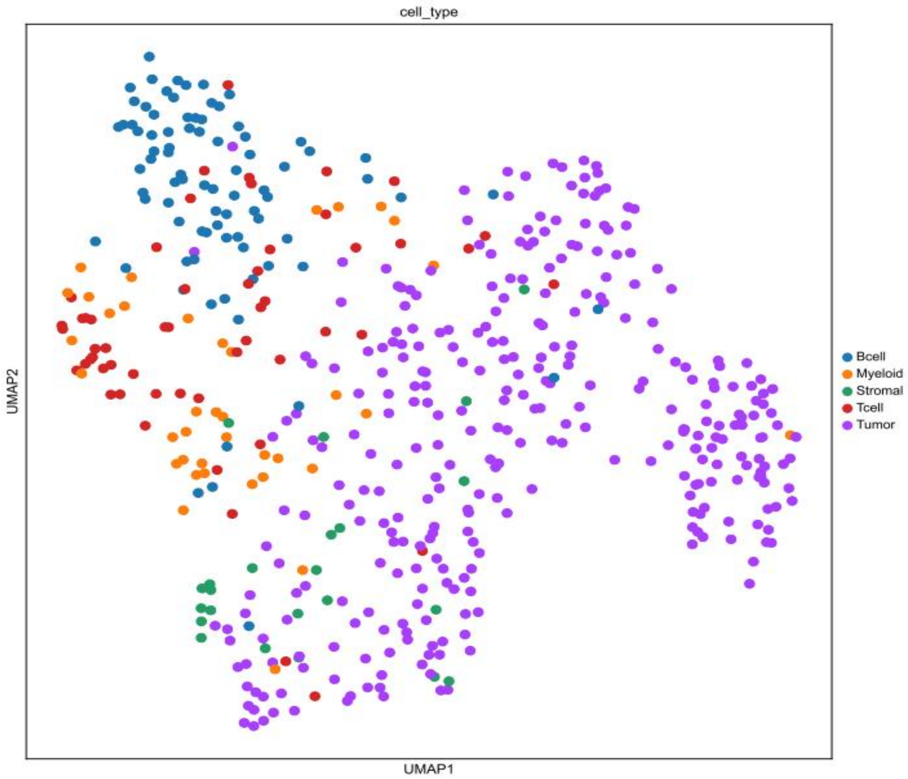

Figure 5

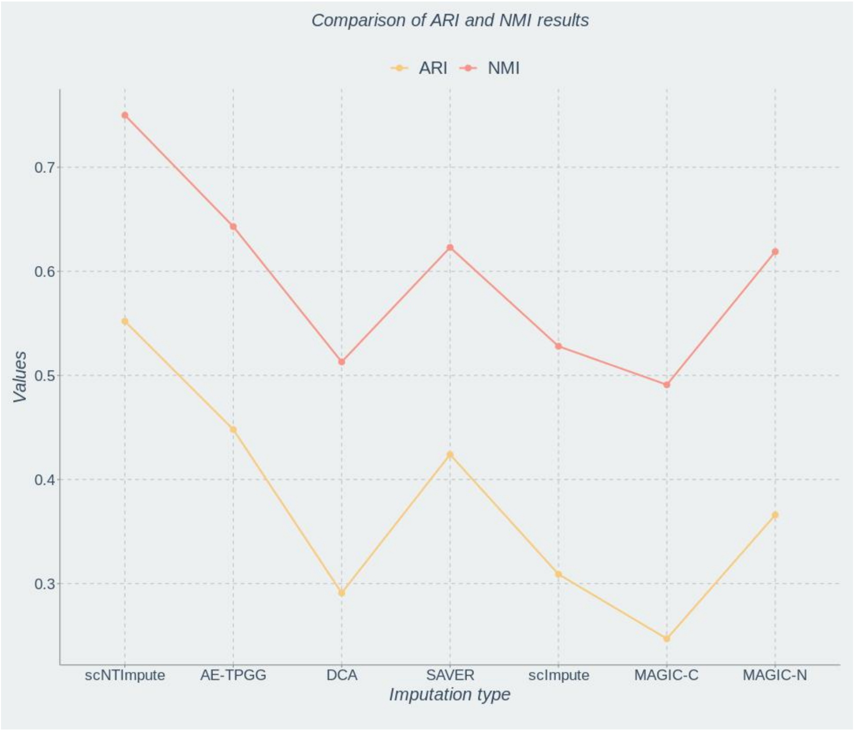

Figure 6

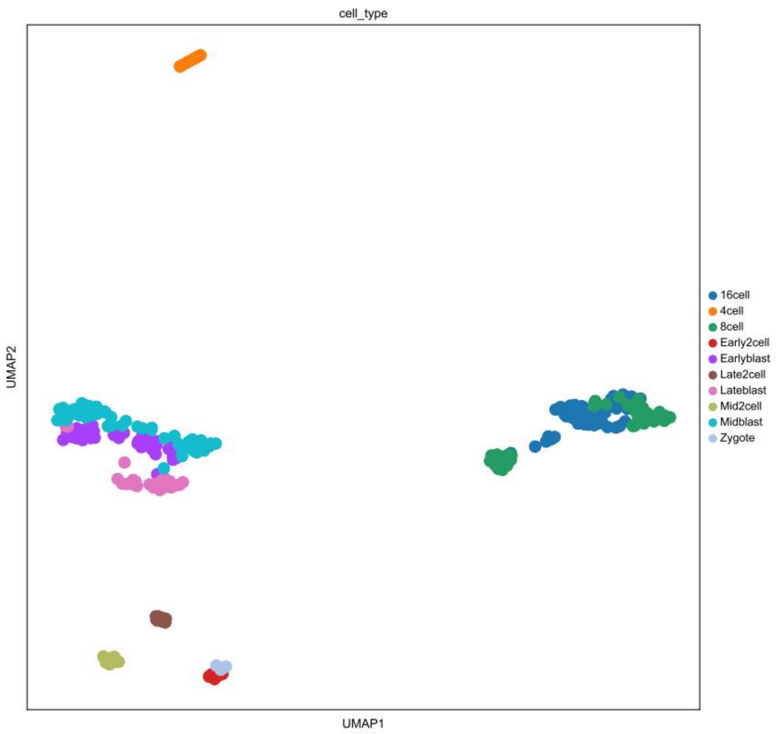

Figure 7

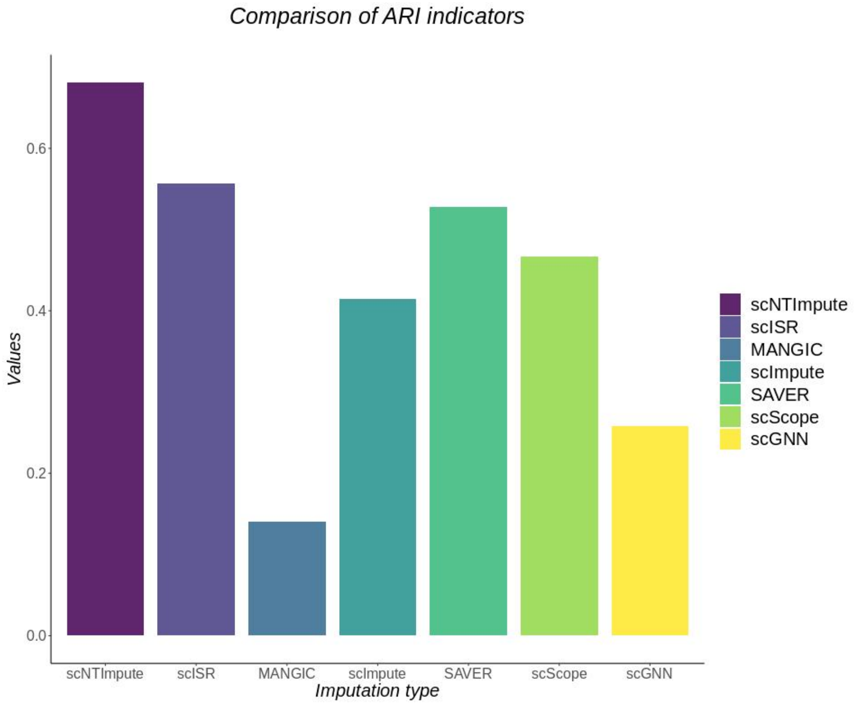

Figure 8

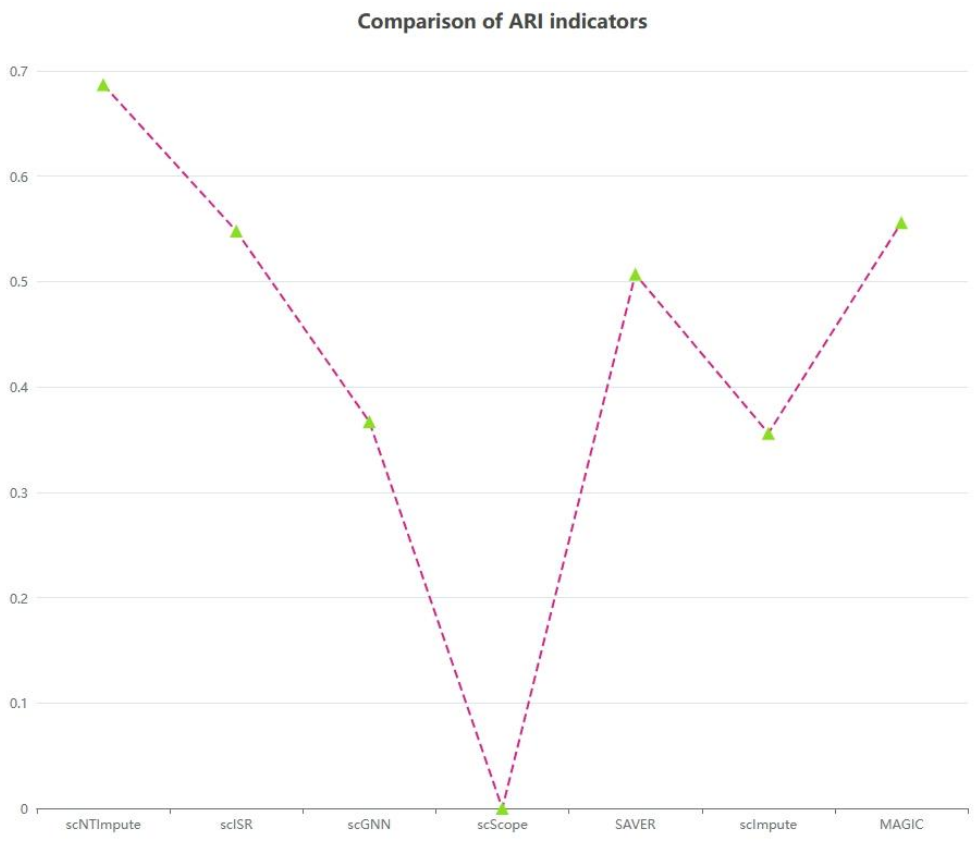

Figure 9

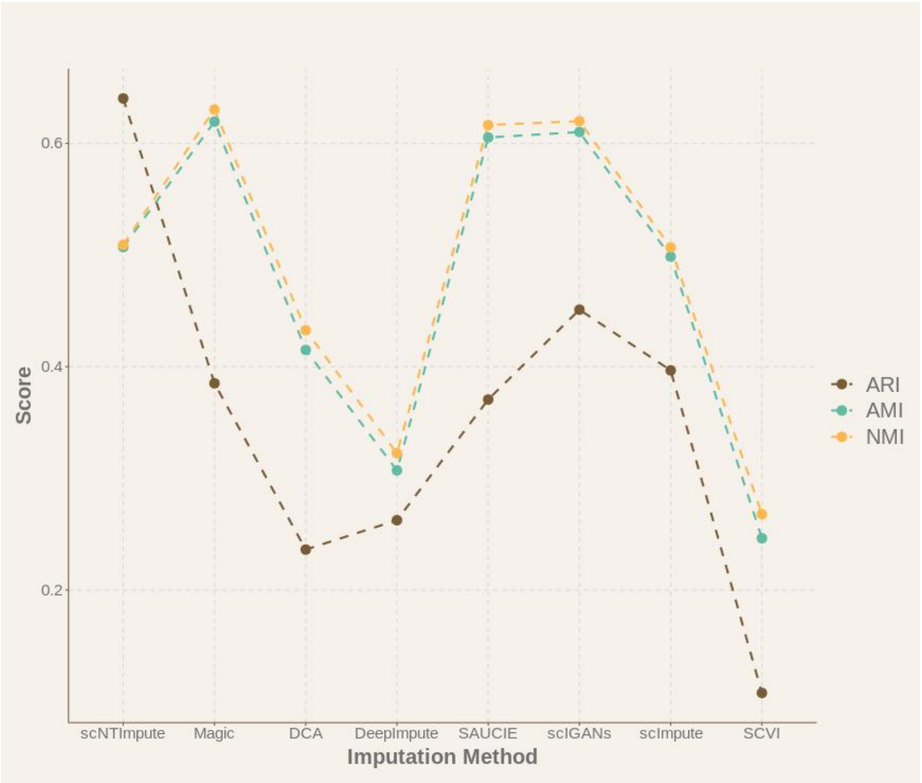

Figure 10

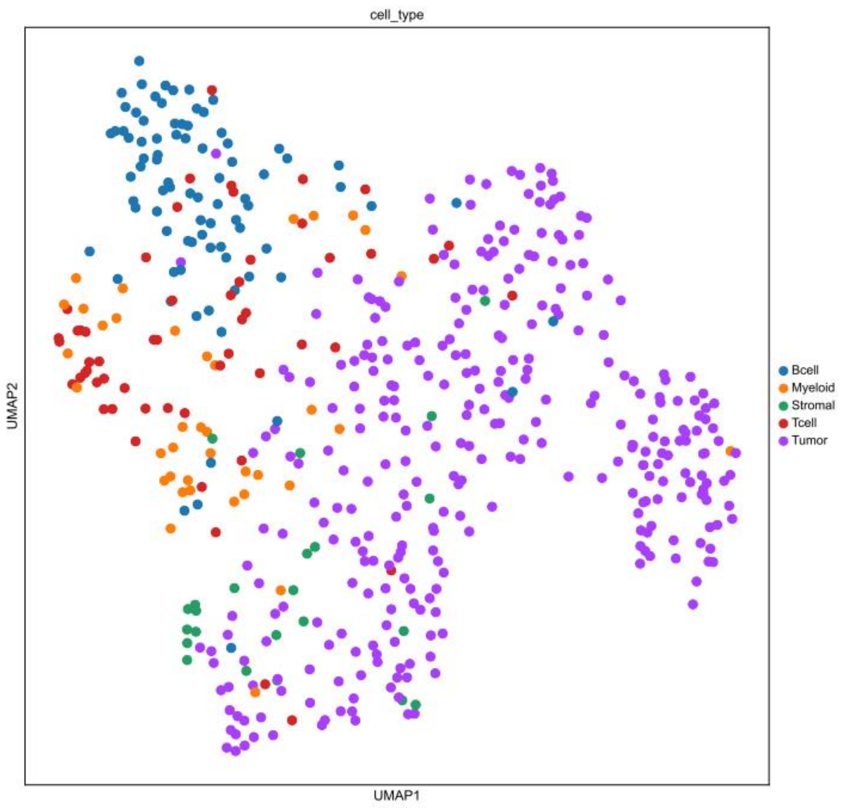

Figure 11

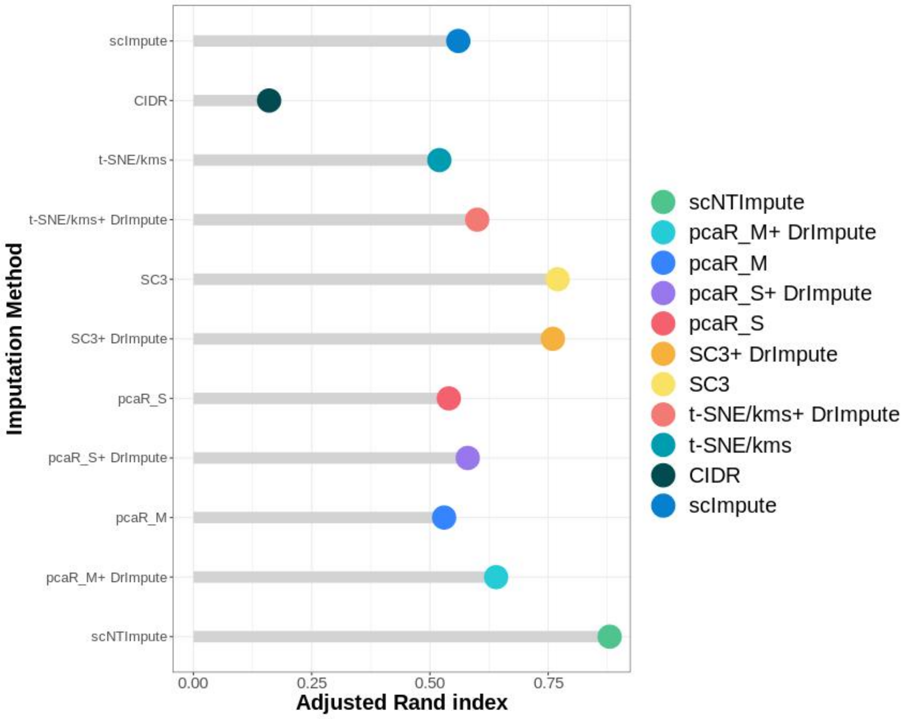

Figure 12

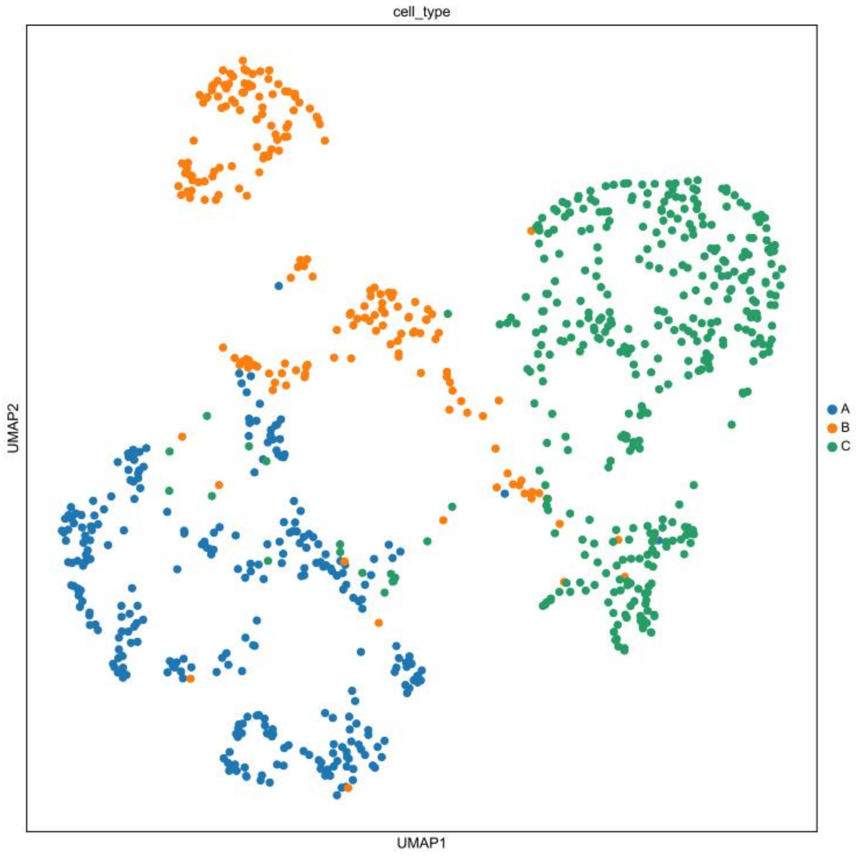

Figure 13

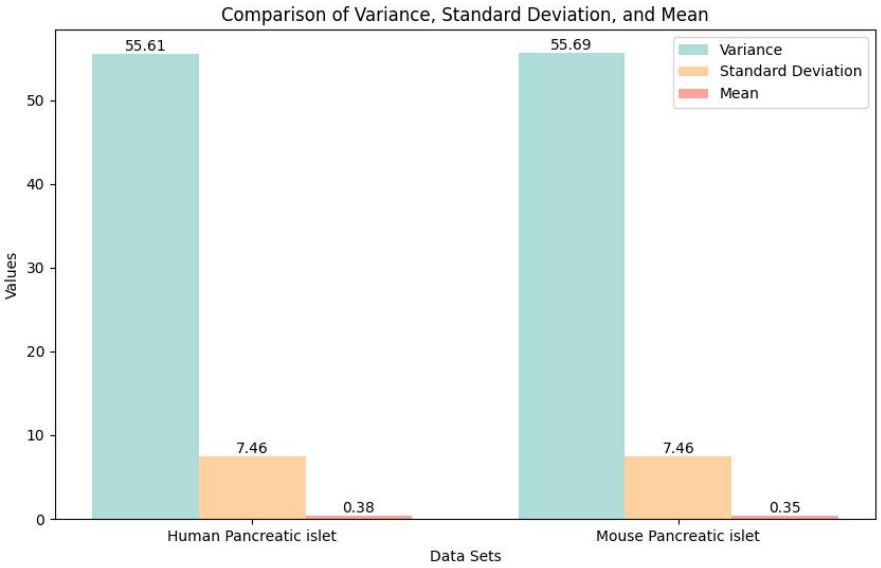

Figure 14

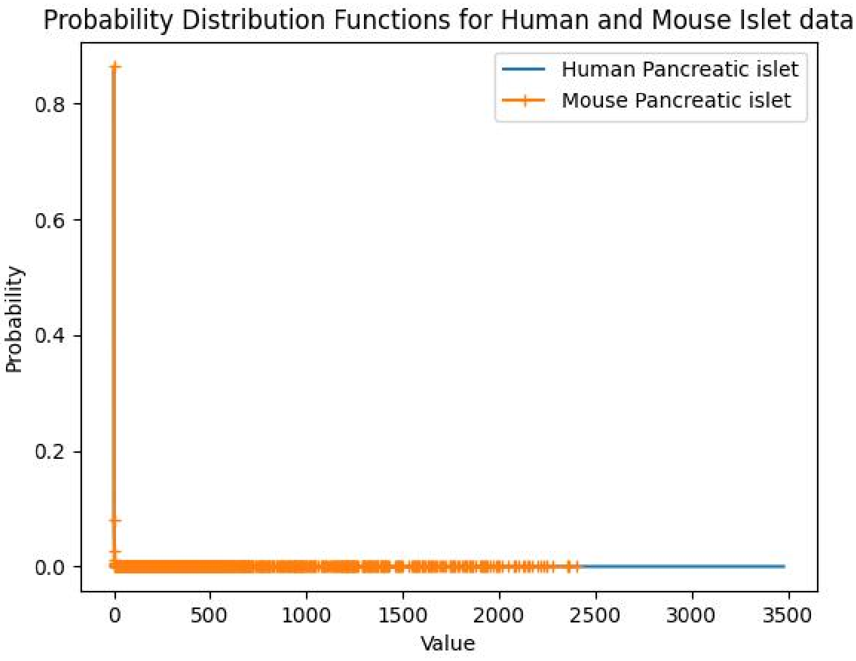

Figure 15

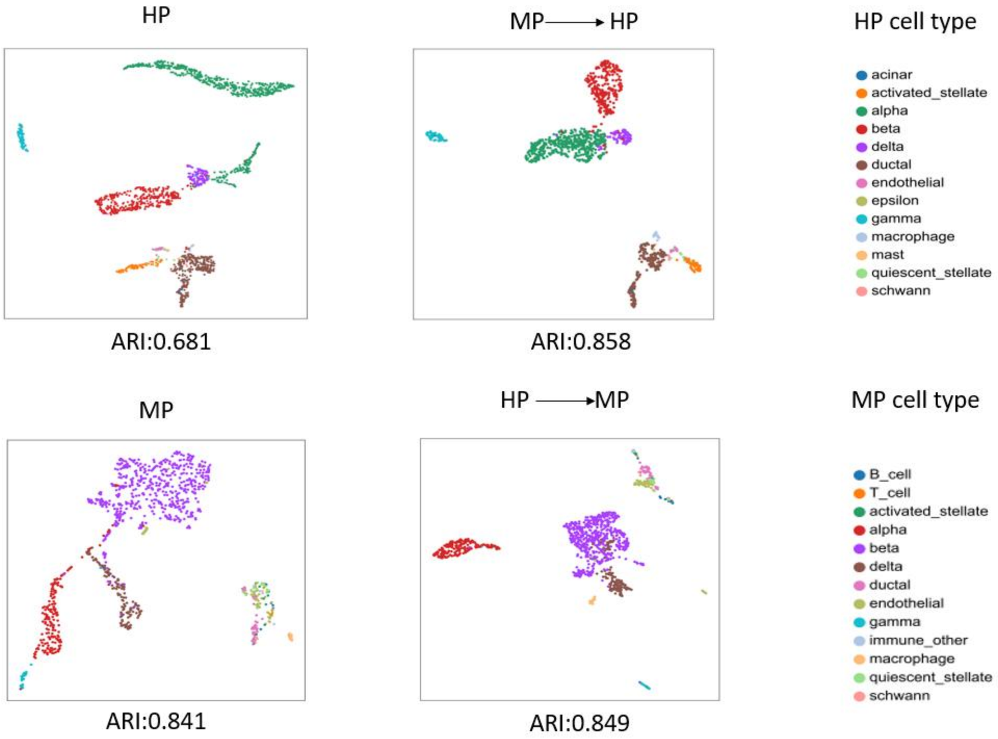

Figure 16

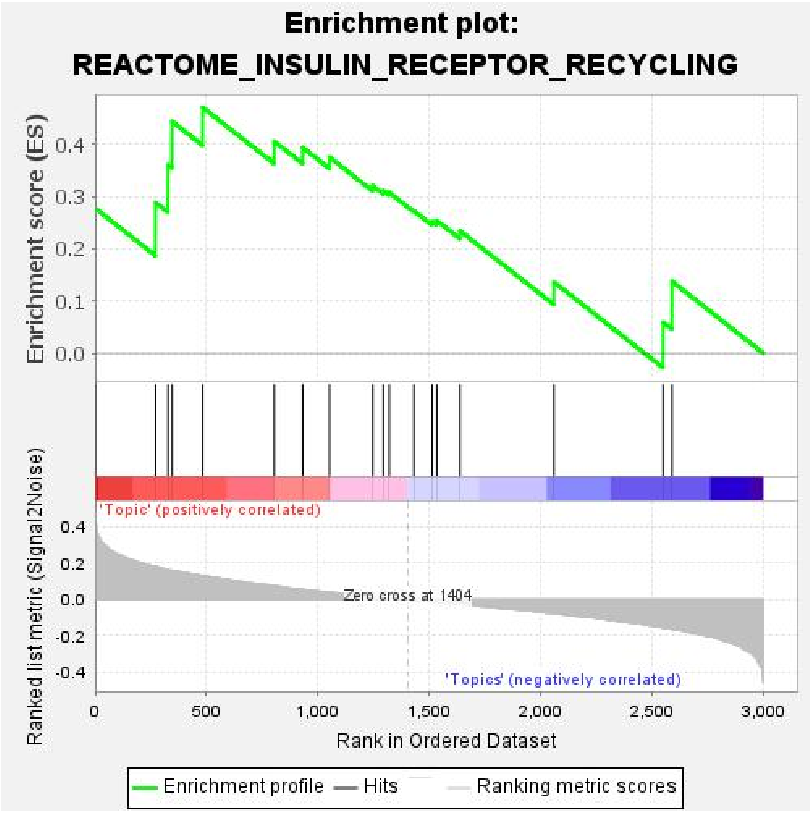

Figure 17

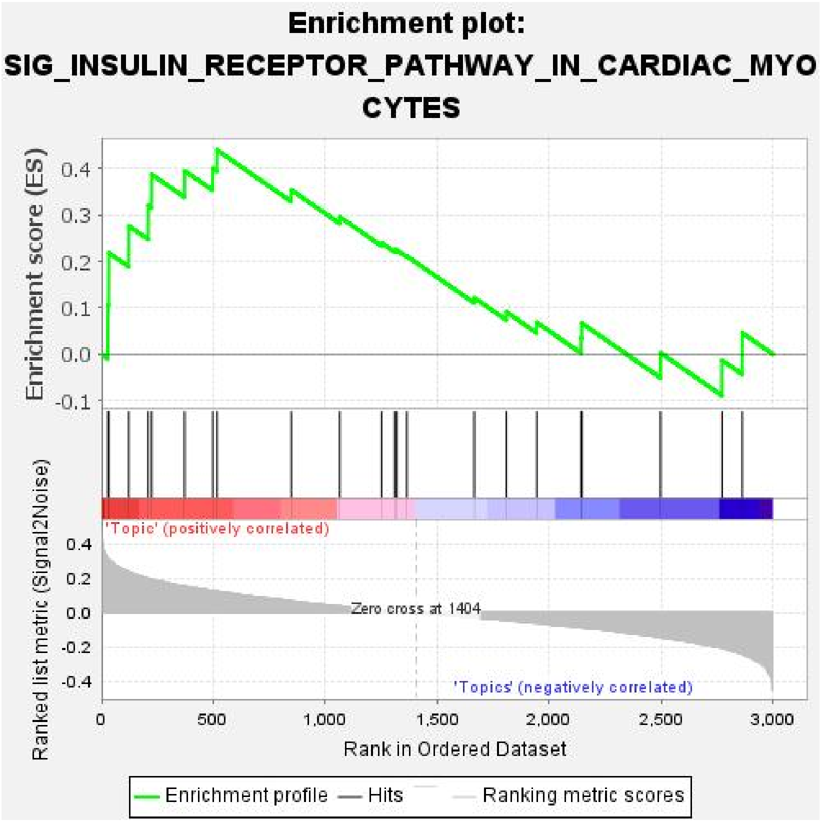

Figure 18

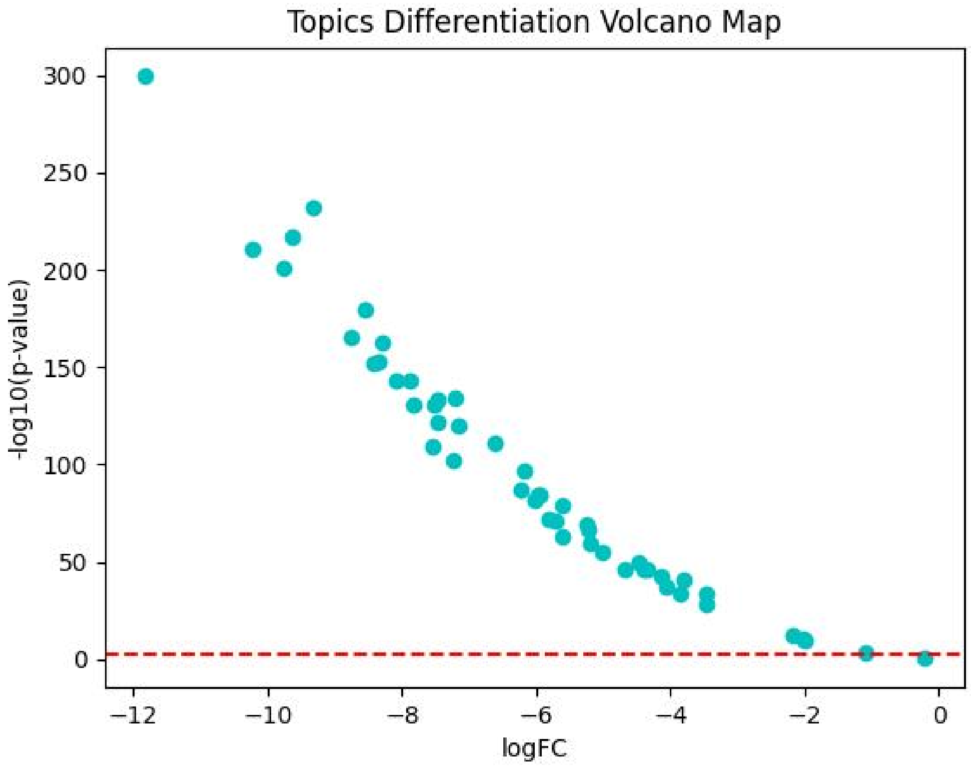

Figure 19

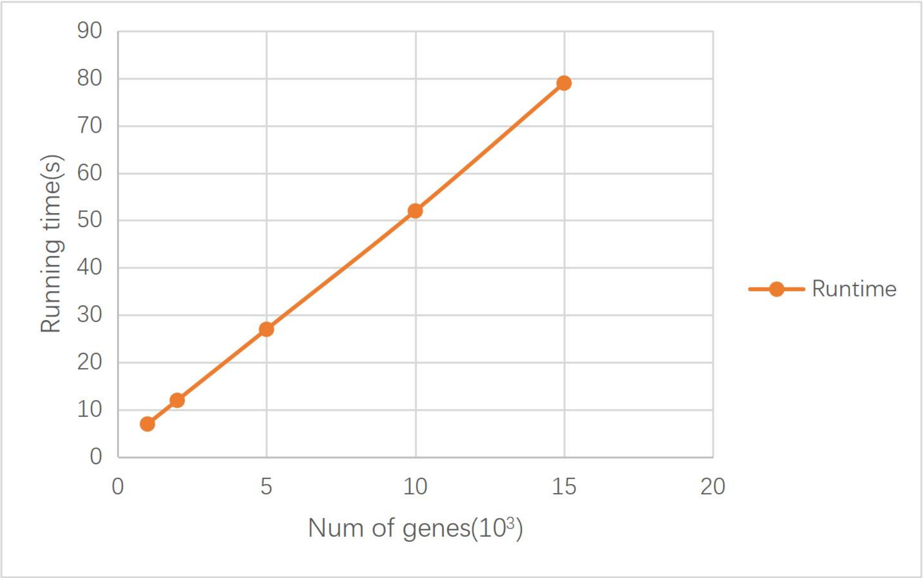

Figure 20

Personal Cover
Click here to access/download;Personal Cover;Response to Reviewers .pdf

Dear Editor:

On behalf of my co-authors, we thank you and the reviewer very much for giving us an opportunity to revise our manuscript entitled "Imputation Methods for Single-Cell RNA-seq Data Using Neural Topic Models" (Original paper ID: GigaScience - GIGA-D-23-00090). We also thank the reviewers of your journal for their comments on our manuscript. Those comments are all valuable and very helpful for revising and improving our paper, as well as the important guiding significance to our researches. We have studied comments carefully and tried our best to make corrections and add contents according to the comments. Enclosed please find the revised manuscript, together with a detailed list of the responses (in blue) regarding the specific points raised by the two reviewers and the editor as below.

We believe that both the manuscript's quality and clarity have improved due to these revisions, and we hope that the paper is now in a form suitable for publication in **GigaScience** as a research article.

Sincerely,

Yueyang Qi

In addition, please register scNTImpute in the bio.tools and SciCrunch.org databases to receive RRID (Research Resource Identification Initiative ID) and biotoolsID identifiers, and include these in your manuscript. Computational workflows should be registered in workflowhub.eu and the DOIs cited in the relevant places in the manuscript. These will facilitate tracking, reproducibility and re-use of your tool.

Reply: Following the editor's suggestion, we have successfully registered scNTImpute on bio.tools and SciCrunch.org databases. Furthermore, we have updated the "Availability of Source Code and Requirements" section of the manuscript to include our RRID and biotoolsID identifiers. We attempted to register scNTImpute on workflowhub.eu to obtain DOIs, but it requires creating or joining a team to register content. Unfortunately, our application to create a team was unsuccessful, which means we are currently unable to obtain DOIs.

Reviewer 1:

General Comments:

The authors have addressed an important topic in scRNA-seq data imputation due to dropout events. The proposed methodology looks suitable for GigaScience in 2023. The experimental results have been properly obtained. The utility of the proposed method has also been demonstrated with software availability. I have few comments:

1. There are already many data imputation methods for scRNA-seq in 2023. The authors may wish to discuss or compare scNTImpute with others.

Reply: We are sorry for not being able to adequately present the results of the model's comparative experiments. Following the reviewer's suggestion, we have added several published imputation methods from 2022 to 2023 to compare with our model, including, scGGAN with AE-TPGG published in 2023, and scISR published in 2022. We performed comparison experiments with scGGAN on the human brain (GSE67835) dataset (specific results in Figure 2 and Table 1); with AE-TPGG on the Deng dataset (GSE45719) (on pages 13 and 14) (specific results in Figure 6 and Table 3); with scISR, scScope (2019), and scGNN (2021) on the Human Pancreatic islet and Romanov (GSE74672) datasets (on page 15) (results are shown in Figure 8, Figure 9).

2. The actual molecular insights can also be enriched to demonstrate the practical values of scNTImpute.

Reply: We believe that the core strength of the scNTImpute model lies in its learnt topics, biological significance of the topic matter from a molecular perspective, as demonstrated by experiments with real scRNA-seq data. In the section "Path enrichment analysis and statistical significance test of scNTImpute topics" (Chapter 3, Section 5), we added new content that explores the biological relevance between the

topics learned in scNTImpute and known gene pathways in humans. In this part of the research, we detected many enriched pathways based on topics learned by scNTImpute from the Human Pancreatic islet dataset, which contains many pathways related to pancreatic function, including the insulin receptor recycling, and cardiac myocyte insulin receptor signaling pathway, etc (Figure 17, Figure 18). Additionally, the topics showed varying levels of significant enrichment in these pathways.

3. Computational complexity analysis or running time should be added for clear demonstration.

Reply: Following the reviewer's suggestion, we have added a section on "Scalability and Efficiency" in which we conducted a time complexity analysis of scNTImpute (on pages 23 and 24). We explore the relationship between runtime and the number of genes. (Figure 20).

4. Source code links can also be added at the end of abstract for easy reading.

Reply: Thanks to the reviewers for their careful reminders. Following the reviewer's suggestion, we have added a link to our source code at the end of our abstract for the convenience of our readers.

5. Statistical significance testing should be properly performed.

Reply: We are so sorry for neglecting proper statistical significance testing. So, we also added the differential analysis of topics in the "Path enrichment analysis and statistical significance test of scNTImpute topics" section, using the topics learnt from the Human Pancreatic islet dataset (on pages 22 and 23). Based on the differences in the enrichment levels of topics within pathway, we found significant variations between topics (Figure 19).

Reviewer 2:

General Comments:

This paper proposed an imputation framework based on a neural network topic model, scNTImpute, for scRNA-seq data. It extracts underlying topic features of scRNA-seq data to help with the inference of cell similarity. Several experiments were conducted to testify the performance of the proposed method. The method is reasonable and the paper is generally well-written. My specific comments are shown as below:

1. I suggest to show the loss function of the model and explain how the loss function make the model achieve the goal of extracting topic features of data.

Reply: We are so sorry for not providing an explanation of the loss function. In the "Topic generation process" section of the original manuscript, the process of acquiring our topics is mainly introduced. So, we have added the model's loss function here and explained how the topic features of single-cell sequencing data are extracted based on this loss function (on pages 26, 27).

2. There is a presumption for transfer learning that the data distribution of source domain should be similar to that of target domain. Therefore, the authors should demonstrate the similarity of the data sets used in the transfer learning section.

Reply: Following the reviewer's suggestion, we have newly added a demonstration of data similarity in the "Transfer learning across single-cell datasets" section. We demonstrate this from two main perspectives. Firstly, mean, variance and standard deviation are commonly used statistical metrics to analyse the features and similarity of the datasets. By examining at these three metrics (Table 6, Figure 14), we find that the degree of dispersion, as well as the central tendency of the two datasets (Human Pancreatic islet and Mouse Pancreatic islet) are very close to each other (on page 19). Secondly, the Probability Distribution Function (PDF) is also helpful for

understanding and analyzing the similarity between datasets (on page 20). By visualizing the PDFs, it is evident that the distributions of the two datasets are highly similar (Figure 15).

3. The overall performance of the proposed method has been demonstrated in the paper. It would be helpful if one or two examples, which show the contribution of the extracted topic features, could be included.

Reply: Following the reviewer's suggestion, we have included a new section titled "Path enrichment analysis and statistical significance test of scNTImpute topics" (on pages 22, 23). In this section, we first investigated whether the topics learnt by scNTImpute are biologically relevant to known genetic pathways in humans. Using the Human Pancreatic Islet dataset, we identify many enriched pathways associated with pancreatic function. Many of these pathways are relevant to pancreatic function (Figure 17, Figure 18). In addition, the difference in the degree of enrichment of topics in the pathway also indicates the significant differences between the topics found in our model. (Figure 19). Of course, the ultimate goal of the scNTImpute model is to use the topics to compute cellular similarities for accurate imputation, which is also described in the "Imputation" section of the paper.
