# Supplementary material for: Imputation method for single-cell RNA-seq data using neural topic model
Source: Gigascience. 2023 Nov 24;12:giad098. doi: 10.1093/gigascience/giad098 (PMC10673642; doi:10.1093/gigascience/giad098)
Supplement: giad098_Response_to_Reviewer_Comments_Original_Submission [file giad098_response_to_reviewer_comments_original_submission.pdf]

Author's Response To Reviewer Comments

Dear Editor:
On behalf of my co-authors, we thank you and the reviewer very much for giving us an opportunity to revise our manuscript entitled “Imputation Methods for Single-Cell RNA-seq Data Using Neural Topic Models” (Original paper ID: GigaScience - GIGA-D-23-00090). We also thank the reviewers of your journal for their comments on our manuscript. Those comments are all valuable and very helpful for revising and improving our paper, as well as the important guiding significance to our researches. We have studied comments carefully and tried our best to make corrections and add contents according to the comments. Enclosed please find the revised manuscript, together with a detailed list of the responses (in blue) regarding the specific points raised by the two reviewers and the editor as below.
We believe that both the manuscript’s quality and clarity have improved due to these revisions, and we hope that the paper is now in a form suitable for publication in GigaScience as a research article.
Sincerely,
Yueyang Qi

In addition, please register scNTImpute in the bio.tools and SciCrunch.org databases to receive RRID (Research Resource Identification Initiative ID) and biotoolsID identifiers, and include these in your manuscript. Computational workflows should be registered in workflowhub.eu and the DOIs cited in the relevant places in the manuscript. These will facilitate tracking, reproducibility and re-use of your tool.
Reply: Following the editor's suggestion, we have successfully registered scNTImpute on bio.tools and SciCrunch.org databases. Furthermore, we have updated the "Availability of Source Code and Requirements" section of the manuscript to include our RRID and biotoolsID identifiers. We attempted to register scNTImpute on workflowhub.eu to obtain DOIs, but it requires creating or joining a team to register content. Unfortunately, our application to create a team was unsuccessful, which means we are currently unable to obtain DOIs.

Reviewer 1:
General Comments:
The authors have addressed an important topic in scRNA-seq data imputation due to dropout events. The proposed methodology looks suitable for GigaScience in 2023. The experimental results have been properly obtained. The utility of the proposed method has also been demonstrated with software availability. I have few comments:

1. There are already many data imputation methods for scRNA-seq in 2023. The authors may wish to discuss or compare scNTImpute with others.
Reply: We are sorry for not being able to adequately present the results of the model's comparative experiments. Following the reviewer’s suggestion, we have added several published imputation methods from 2022 to 2023 to compare with our model, including, scGGAN with AE-TPGG published in 2023, and scISR published in 2022. We performed comparison experiments with scGGAN on the human brain (GSE67835) dataset (specific results in Figure 2 and Table 1); with AE-TPGG on the Deng dataset (GSE45719) (on pages 13 and 14) (specific results in Figure 6 and Table 3); with scISR, scScope (2019), and scGNN (2021) on the Human Pancreatic islet and Romanov (GSE74672) datasets (on page 15) (results are shown in Figure 8, Figure 9).

2. The actual molecular insights can also be enriched to demonstrate the practical values of scNTImpute.
Reply: We believe that the core strength of the scNTImpute model lies in its learnt topics, biological significance of the topic matter from a molecular perspective, as demonstrated by experiments with real scRNA-seq data. In the section "Path enrichment analysis and statistical significance test of scNTImpute topics" (Chapter 3, Section 5), we added new content that explores the biological relevance between the topics learned in scNTImpute and known gene pathways in humans. In this part of the research, we detected many enriched pathways based on topics learned by scNTImpute from the Human Pancreatic islet dataset, which contains many pathways related to pancreatic function, including the insulin receptor

recycling, and cardiac myocyte insulin receptor signaling pathway, etc (Figure 17, Figure 18). Additionally, the topics showed varying levels of significant enrichment in these pathways.

3. Computational complexity analysis or running time should be added for clear demonstration.
Reply: Following the reviewer's suggestion, we have added a section on "Scalability and Efficiency" in which we conducted a time complexity analysis of scNTImpute (on pages 23 and 24). We explore the relationship between runtime and the number of genes. (Figure 20).

4. Source code links can also be added at the end of abstract for easy reading.
Reply: Thanks to the reviewers for their careful reminders. Following the reviewer's suggestion, we have added a link to our source code at the end of our abstract for the convenience of our readers.

5. Statistical significance testing should be properly performed.
Reply: We are so sorry for neglecting proper statistical significance testing. So, we also added the differential analysis of topics in the "Path enrichment analysis and statistical significance test of scNTImpute topics" section, using the topics learnt from the Human Pancreatic islet dataset (on pages 22 and 23). Based on the differences in the enrichment levels of topics within pathway, we found significant variations between topics (Figure 19).

Reviewer 2:
General Comments:
This paper proposed an imputation framework based on a neural network topic model, scNTImpute, for scRNA-seq data. It extracts underlying topic features of scRNA-seq data to help with the inference of cell similarity. Several experiments were conducted to testify the performance of the proposed method. The method is reasonable and the paper is generally well-written. My specific comments are shown as below:

1. I suggest to show the loss function of the model and explain how the loss function make the model achieve the goal of extracting topic features of data.
Reply: We are so sorry for not providing an explanation of the loss function. In the "Topic generation process" section of the original manuscript, the process of acquiring our topics is mainly introduced. So, we have added the model's loss function here and explained how the topic features of single-cell sequencing data are extracted based on this loss function (on pages 26, 27).

2. There is a presumption for transfer learning that the data distribution of source domain should be similar to that of target domain. Therefore, the authors should demonstrate the similarity of the data sets used in the transfer learning section.
Reply: Following the reviewer's suggestion, we have newly added a demonstration of data similarity in the "Transfer learning across single-cell datasets" section. We demonstrate this from two main perspectives. Firstly, mean, variance and standard deviation are commonly used statistical metrics to analyse the features and similarity of the datasets. By examining at these three metrics (Table 6, Figure 14), we find that the degree of dispersion, as well as the central tendency of the two datasets (Human Pancreatic islet and Mouse Pancreatic islet) are very close to each other (on page 19). Secondly, the Probability Distribution Function (PDF) is also helpful for understanding and analyzing the similarity between datasets (on page 20). By visualizing the PDFs, it is evident that the distributions of the two datasets are highly similar (Figure 15).

3. The overall performance of the proposed method has been demonstrated in the paper. It would be helpful if one or two examples, which show the contribution of the extracted topic features, could be included.
Reply: Following the reviewer's suggestion, we have included a new section titled "Path enrichment analysis and statistical significance test of scNTImpute topics" (on pages 22, 23). In this section, we first investigated whether the topics learnt by scNTImpute are biologically relevant to known genetic pathways in humans. Using the Human Pancreatic Islet dataset, we identify many enriched pathways associated with pancreatic function. Many of these pathways are relevant to pancreatic function (Figure 17, Figure 18). In addition, the difference in the degree of enrichment of topics in the pathway also indicates the significant differences between the topics found in our model. (Figure 19). Of course, the ultimate goal of the

scNTImpute model is to use the topics to compute cellular similarities for accurate imputation, which is also described in the "Imputation" section of the paper.
